# The Plastidial Protein Acetyltransferase GNAT1 Forms a Complex With GNAT2, yet Their Interaction Is Dispensable for State Transitions

**DOI:** 10.1016/j.mcpro.2024.100850

**Published:** 2024-09-28

**Authors:** Annika Brünje, Magdalena Füßl, Jürgen Eirich, Jean-Baptiste Boyer, Paulina Heinkow, Ulla Neumann, Minna Konert, Aiste Ivanauskaite, Julian Seidel, Shin-Ichiro Ozawa, Wataru Sakamoto, Thierry Meinnel, Dirk Schwarzer, Paula Mulo, Carmela Giglione, Iris Finkemeier

**Affiliations:** 1 Plant Physiology, Institute of Plant Biology and Biotechnology (IBBP), University of Münster, Münster, Germany; 2 Université Paris-Saclay, CEA, CNRS, Institute for Integrative Biology of the Cell (I2BC), Gif-sur-Yvette, France; 3 Central Microscopy, Max Planck Institute for Plant Breeding Research, Köln, Germany; 4 Department of Life Technologies, Molecular Plant Biology, University of Turku, Turku, Finland; 5 Interfaculty Institute of Biochemistry, University of Tübingen, Tübingen, Germany; 6 Institute of Plant Science and Resources (IPSR) Okayama University, Kurashiki, Okayama, Japan

**Keywords:** acetyltransferase, GNAT, chloroplasts, *N*-acetylation, protein interaction, cross-linking, mass spectrometry, Arabidopsis

## Abstract

Protein *N*-acetylation is one of the most abundant co- and post-translational modifications in eukaryotes, extending its occurrence to chloroplasts within vascular plants. Recently, a novel plastidial enzyme family comprising eight acetyltransferases that exhibit dual lysine and N-terminus acetylation activities was unveiled in Arabidopsis. Among these, GNAT1, GNAT2, and GNAT3 reveal notable phylogenetic proximity, forming a subgroup termed NAA90. Our study focused on characterizing GNAT1, closely related to the state transition acetyltransferase GNAT2. In contrast to GNAT2, GNAT1 did not prove essential for state transitions and displayed no discernible phenotypic difference compared to the wild type under high light conditions, while *gnat2* mutants were severely affected. However, *gnat1* mutants exhibited a tighter packing of the thylakoid membranes akin to *gnat2* mutants. *In vitro* studies with recombinant GNAT1 demonstrated robust N-terminus acetylation activity on synthetic substrate peptides. This activity was confirmed *in vivo* through N-terminal acetylome profiling in two independent *gnat1* knockout lines. This attributed several acetylation sites on plastidial proteins to GNAT1, reflecting a subset of GNAT2’s substrate spectrum. Moreover, co-immunoprecipitation coupled with mass spectrometry revealed a robust interaction between GNAT1 and GNAT2, as well as a significant association of GNAT2 with GNAT3 - the third acetyltransferase within the NAA90 subfamily. This study unveils the existence of at least two acetyltransferase complexes within chloroplasts, whereby complex formation might have a critical effect on the fine-tuning of the overall acetyltransferase activities. These findings introduce a novel layer of regulation in acetylation-dependent adjustments in plastidial metabolism.

## Highlights

• GNAT1 is a plastidial acetyltransferase with dual activity in Arabidopsis.
• The N-terminal substrates of GNAT1 form a subset of the substrate set of GNAT2.
• Predicted structure models of GNAT1 and GNAT2 show a high architectural similarity.
• Absence of GNAT1 neither impacts state transitions nor acclimation to high light.
• Stable interactions were observed between GNAT1 & GNAT2 and GNAT2 & GNAT3.

## Graphical Abstract

**Figure:**
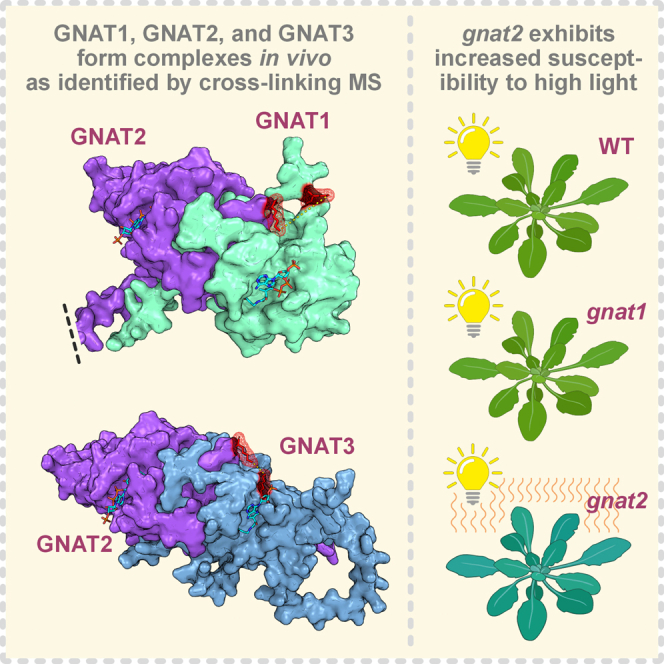

## In Brief

Protein acetylation is a common cotranslational and posttranslational modification in chloroplasts. Here, we characterize GNAT1, an acetyltransferase that adds acetyl groups to N-termini and lysine side chains. GNAT1 is closely related to GNAT2, which regulates state transitions of the photosynthetic antenna proteins. Our findings show shared N-terminal substrate sites between GNAT1 and GNAT2 in vivo. Both enzymes form a complex, but this interaction is not essential for state transitions.

The acetylation of amino groups within proteins is one of the major post-translational modifications (PTMs) in all organisms. N-terminal acetyltransferases (NATs) or lysine acetyltransferases (KATs) catalyze the transfer of acetyl groups from the donor substrate acetyl coenzyme A (acetyl-CoA) to the targeted amino groups, which are either the α-amino group of protein N-termini or the ε-amino group of internal lysine residues (1). In contrast to N-terminal acetylation (NTA), the acetylation of lysine residues (KA) is reversible, whereby lysine deacetylases (KDACs) are responsible for the removal of acetyl groups from proteins. Furthermore, KA occurs post-translationally, whereas NTA is described mainly as a co-translational modification with the exception of N-terminally processed proteins, such as those imported to chloroplasts and mitochondria (2, 3, 4, 5).

With the help of state-of-the-art mass spectrometry (MS), the regulation and abundance of NTA as well as KA in proteomes of diverse organisms was investigated in detail in recent years. Since KA was discovered in the mid-1960s on histones (6), research on this modification was for several decades predominantly centered on histone acetylation and its effects on chromatin remodeling and gene regulation. Around 40 years later, significant technical progress and newly introduced immunoprecipitation-based enrichment protocols allowed an immense increase in the number of KA-sites reported to date (7, 8, 9, 10). Recent studies revealed, for instance, about 7000 KA-sites on more than 2500 proteins of human HeLa cells or, in the case of *Arabidopsis thaliana*, more than 5000 sites identified on about 2600 seedling or plant leaf proteins (11, 12, 13, 14). Acetylation of terminal amino acids in eukaryotes is known as one of the most widespread protein modifications. For example, 85% of the human proteome and about 81% of the Arabidopsis proteome undergo NTA (15, 16, 17).

Since plants have a sessile lifestyle, they depend on fast and effective acclimation mechanisms to regulate their cellular metabolism. A variety of specific and tightly regulated signaling processes allow plants to react within seconds when environmental conditions are rapidly changing, for instance, in the case of fluctuating light intensities or qualities as well as changing temperatures and nutrient or water availabilities (18, 19). Furthermore, as photosynthesizing organisms, plants harbor chloroplasts in their green tissues. These organelles are able to convert light energy into chemical energy to produce organic compounds, which feed into a multitude of metabolic pathways (20, 21). Several studies demonstrated that KA as well as NTA are widespread modifications in chloroplasts, whereby the fraction of plastidial proteins undergoing post-translational NTA is rather high in comparison to its proportion in the cytosol (13, 22, 23, 24, 25).

Eight plastid-localized acetyltransferases were recently identified as exhibiting dual KA and NTA activities (23, 26, 27). These enzymes form a novel family of chloroplast acetyltransferases, which is subordinated to the superfamily of general control non-repressible 5 (GCN5)-related *N*-acetyltransferases (GNATs). Recent studies revealed the dual specificity of these plastidial GNATs, a property that has been debated and rather rejected for the cytosolic NAT enzymes for some time (28, 29, 30). However, the investigation of their physiological roles is still in its beginnings, and a significant role in photosynthesis was recently discovered for GNAT2 (27). Arabidopsis mutant lines deficient in GNAT2 lost their ability to perform state transitions and did not form the state transition complex consisting of photosystem I and light-harvesting complex II proteins (PSI-LHCII) (27). Furthermore, *gnat2* lines exhibited downregulated KA of specific photosynthetic proteins including subunits of PSI, PSII, and LHCII as well as a retarded growth phenotype under fluctuating light conditions (27, 31). On the level of structural thylakoid membrane organization, *gnat2* mutant plants resemble the phenotype of plants deficient in the kinase STN7 concerning grana packing and defective response to the shift from darkness to light (27, 31, 32). In the state transition process, a pool of LHCII trimers (L-LHCII) can change its association from either PSII to PSI or vice versa, thereby serving as an additional antenna. Depending on the prevalent light qualities and quantities, the excitation energy harvested by LHCII proteins can be dynamically distributed between PSII and PSI to enable a balanced flow of electrons within the photosynthetic electron transport chain (33, 34, 35). For more than 20 years, it is well known that changes in the L-LHCII association are regulated by reversible phosphorylation of the LHCII subunits LHCB1 and LHCB2 catalyzed by the kinase STN7 and the phosphatase PPH1/TAP38 (36, 37). PSII-favoring light conditions trigger the phosphorylation of LHCB1 and 2, whereas PSI-favoring illumination leads to dephosphorylation of both subunits (38, 39). Intriguingly, *gnat2* plants, just like STN7 deficient mutants, are not able to adapt to PSII-favoring light by directing the LHCII trimers to associate with PSI, even if the phosphorylation state of LHCB1 and 2 is not impaired in *gnat2* (27, 32). These findings clearly highlight lysine acetylation, next to phosphorylation, as an additional layer of post-translational regulation in the network of chloroplast light responses. Nonetheless, it also raises numerous questions, such as about the precise mechanism that mediates the acetylation-dependent regulation of state transitions and which other metabolic pathways within the chloroplast require KA or NTA of proteins.

In this study, we investigated the function of GNAT1, a yet uncharacterized member of the new chloroplast-localized GNAT family. The acetyltransferase domain of GNAT1 reveals an especially high degree of similarity to the corresponding domain of GNAT2 (26), leading to speculations regarding a potentially similar function *in planta*. Furthermore, this study reports on an acetyltransferase complex within the chloroplast, whereby complex formation might have a critical effect on the fine-tuning of the overall acetyltransferase activities and substrate specificities. These findings open an additional promising perspective in the study of KA as well as of NTA within chloroplasts.

## Experimental Procedures

### Heterologous Expression and Purification of Recombinant GNAT1 and GNAT2 Proteins

The plasmid for heterologous expression of His_6_-GNAT1 was cloned as described previously for GNAT2/NSI (27), using the vector system pQE-30 (Qiagen). The open reading frame of GNAT1 (AT1G26220.1) was amplified from Arabidopsis cDNA with the Phusion High-Fidelity DNA Polymerase (Thermo), whereby the GNAT1 coding sequence was trimmed by the region coding for the predicted transit peptide (36 N-terminal amino acids). The following primers were used: GNAT1_pQE-30_for, 5′-CTAGGATCCGCTGCAATGCAAC and GNAT1_pQE-30_rev, 5′-CAACCCGGGTTATTTCTTGTTTCTCTGT. Recombinant His_6_-GNAT2 protein was overexpressed and purified as reported by Koskela and coworkers, while the procedure was slightly modified in the case of GNAT1 (27). In brief, recombinant His_6_-GNAT1 was expressed in *the E. coli* strain SoluBL21 (Genlantis) induced with 0.5 mM IPTG (Roth) for 15 h at 21 °C. Cells were harvested, resuspended in cold buffer (100 mM K-phosphate, pH 8.0, 300 mM NaCl, 10% [v/v] glycerol, protease inhibitor cocktail [Sigma-Aldrich]), and disrupted with a French Press. Afterward, 5 mM DTT and 10 units of lysozyme were added and His_6_-GNAT1 was purified from the soluble phase by Ni-NTA affinity chromatography (HisTrap FF, Cytiva) using the FPLC-system ÄKTA go (Cytiva).

### HPLC-Based N_ε_-Lys- and N_α_-N-terminus Acetylation Assay

His_6_-GNAT1 and His_6_-GNAT2 were analyzed concerning their N_ε_-Lys- and N_α_-N-terminus acetylating activities by using artificial peptides, which function as KAT or NAT substrates, respectively (26, 40). The sequences of the substrate probes generally comprise a modified lysine residue at position nine that carries a dinitroaniline group to allow the specific detection of the peptides by recording their absorbance at 360 nm. A lysine side chain at position five is either free to allow KA by a KAT or is blocked by an acetyl group (NTA peptide substrate). In the case of the NTA peptide probes, substrates with varying N-terminal amino acids were analyzed (Ala, Gly, Ser, Thr, Val, Met, or Leu), whereas the KA-specific probe generally provides an N-terminal Ala residue with a blocked N_α_-amino group. In analogy to the substrates reported by Seidel and coworkers, the KA peptide substrate contains an additional modified lysine residue at position three that is linked to an amino aminobenzoic acid moiety via its N_ε_-amino group (40). In the NTA substrate probe, position three corresponds to an unmodified Gln residue. In summary, the KA substrate peptide sequence is, here in one-letter code: ac-A-A-K(oAba)-G-A-K-A-A-K(Dnp)-Ahx-*r*-*r*-*r* and the sequence of the NTA substrate X-A-Q-G-A-K(ac)-A-A-K(Dnp)-Ahx-*r*-*r*-*r*, whereby oAba refers to ortho-aminobenzoic acid, Dnp to dinitroaniline, Ahx to 6-aminohexanoic acid, and *r* to D-arginine. For measuring catalytic activities of the recombinant His_6_-GNAT proteins, His_6_-GNAT1, His_6_-GNAT2, or both together, were incubated with the substrate peptide (100 μM) in reaction buffer (150 mM K-phosphate, pH 8.0, 50 mM NaCl, protease inhibitor cocktail [Sigma-Aldrich]) and the reaction was started by addition of acetyl-CoA (200 μM). From time points between 0 and 4 h, 11 μl-aliquots were collected and the reaction was stopped with 99 μl trifluoroacetic acid (TFA; final concentration 2% [v/v]). Reaction products were analyzed by using a reversed-phase HPLC chromatograph (Shimadzu), as previously described (27). Reaction rates were calculated from the peak areas of the chromatogram corresponding to the respective peptide probe.

### Computational Analysis of AlphaFold 2-Predicted GNAT Structure Models

The GNAT structure models presented here were obtained from the AlphaFill database (www.alphafill.eu; (41)), which integrates missing ligands, cofactors, and ions into the protein structures provided by the AlphaFold 2 protein structure database (42). AlphaFold 2 Multimer was used to model structures of protein complexes (version 2.3.1; (43)). Further processing steps, such as computational removal of transit peptide sequences and superimpositions of GNAT structures, were performed by using the software PyMOL (The PyMOL Molecular Graphics System, Version 4.5 Schrödinger, LLC). The protein sequence alignment of GNAT1 and GNAT2 (without transit peptide) was derived by using the PRALINE Multiple Sequence Alignment tool (44, 45).

### Plant Material

*A. thaliana* plant lines were cultivated in a 8 h light/16 h darkness regime at a PPFD of 120 μmol · m^-2^ · s^-1^. All lines presented in this study are based on the ecotype Col-0. The mutant lines overexpressing GNAT1-, GNAT2-, or GNAT3-GFP, respectively, were described in (26), while the control line overexpressing chloroplast-targeted GFP was provided by Markus Schwarzländer, University of Münster, Germany (46). Seeds of the T-DNA insertion mutants SALK_062388 (*gnat1-1*) and SALK_150736 (*gnat1-2*) were ordered from NASC and the T-DNA line SALK_033944 (*gnat2-1*, *nsi-1*) was characterized previously (27).

High light and darkness treatment were applied on seedlings, which were grown on sterile agar plates containing ½ MS medium and cultivated for 8 days under long-day conditions (16 h light/8 h darkness, PPFD of 120 μmol · m^−2^ · s^−1^). Photosynthetic performance was determined by detecting PSII-specific chlorophyll fluorescence signals via the IMAGING-PAM fluorometer before the treatment and after the indicated number of days under darkness or highlight (PPFD = 800 μmol ∙ m^−2^ ∙ s^−1^) regime. In total, three plates were analyzed per condition, each with 15 individual seedlings per plant line that were selected for fluorescence detection.

### Genotyping of T-DNA Lines

The two Arabidopsis lines *gnat1-1* and *gnat1-2* were screened by PCR to amplify a DNA fragment of the intact gene locus as well as of a region covering the locus of the T-DNA insertion. In the first case, the primer pairs *gnat1-1*_LP, 5′-GCAAGAAAGAATGCAGCAAAC and *gnat1-1*_RP, 5′-AACCATTCCTTTGATCCCATC, as well as *gnat1-2*_LP, 5′- CATTCCTGAATTGCAGGAGAG and *gnat1-2*_RP, 5′-TGTAAATCCTCAATCAACCGC were used. To confirm the insertion of T-DNA, each RP-primer was combined with the T-DNA specific oligonucleotide SALK_LB, 5′-GATTTAGTGCTTTACGGCACCTC to examine the T-DNA orientation suggested by the Salk Institute Genomic Analysis Laboratory. To reveal “inverse” T-DNA insertion, the primers *gnat1-1*_LP and *gnat1-2*_LP were used in combination with SALK_LB, respectively. The absence of mRNA coding for GNAT1 was verified by endpoint RT-PCRs proving both, the absence/presence of the full-length transcript and of an mRNA fragment, which covers the region downstream of the T-DNA insertion site (gnat1_P1_for, 5′-CATGTTTCTCGGAGGCACAATCTC; gnat1_P2_for, 5′-GATCTCGAATCCAGAGGCTTTC; gnat1_P3_rev, 5′- CAAACTTTTTATTTCTTGTTTCTCTGTTTG). As a reference, additional RT-PCRs were performed allowing the amplification of a fragment of the ubc21-transcript (Ubiquitin-conjugating enzyme 21; ubc21_for, 5′-CTTTGCAACCTCCTCAAGTTC; ubc21_rev, 5′-CATCATCATCCTTTCTTAGGCATAG). For RT-PCR, RNA was isolated from leaf material by phenol-chloroform-isoamyl alcohol (25:24:1) extraction, and subsequent precipitation of RNA was achieved by using 50% isopropanol. Afterward, the RNA preparation was further purified by precipitation with 2 M LiCl and washing with 70% ethanol. For cDNA synthesis, genomic DNA was digested with the RNase-free DNase RQ1 (Promega), and cDNA was synthesized with the help of the FastGene Scriptase II cDNA kit (NIPPON Genetics).

### Cloning of GNAT1, 2, and 3 for Bimolecular Fluorescence Complementation (BiFC) Assays

The open reading frames without STOP codon of GNAT1 (AT1G26220.1), GNAT2 (AT1G32070.1), and GNAT3 (AT4G19985.1) were recombined from entry clones into the Gateway vectors pBatTL-B-sYFPc and pBatTL-B-sYFPn (47, 48) by performing LR reactions. These vector systems allow C-terminal fusions of either the C-terminal or the N-terminal domain of a split-YFP to the protein of interest. In the case of GNAT1 and 2, coding DNA sequences amplified from *Arabidopsis* (Col-0) cDNA were first cloned into the pGWR8 vector (49) and then transferred to the pENTR/D-TOPO (Thermo Fisher) gateway entry vector system by type II endonuclease restriction and subsequent DNA ligation. The entry vector containing GNAT3 was generated by using the pENTR/D-TOPO kit directly in combination with the PCR product amplified from *Arabidopsis* (Col-0) cDNA. The following primers were used to obtain GNAT1, 2, and 3 coding sequences, respectively: gnat1_pENTR_for, 5′-TATACCCGGGATGTTTCTCGGAGG; gnat1_pENTR_rev, 5′-TATAGGATCCTTTCTTGTTTCTCTGTTTGC; gnat2_pENTR_for, 5′-TATACCCGGGATGCTACTAATCCCA; gnat2_pENTR_rev, 5′-TATAGGATCCCTTTGGGTACCAAAACATG; gnat3_pENTR_for, 5′-CACCCTTAAGGAAATGGGTTTG; gnat3_pENTR_rev, 5′-TGCCTCCAAGCTCTTTGTGA. Vector constructs were confirmed by sequencing and used for the transient transformation of *Arabidopsis* (Col-0) protoplasts.

### Protoplast Isolation and Transformation Followed by Bimolecular Fluorescence Complementation (BiFC) Assays *via* confocal Laser Scanning Microscopy (CLSM)

Protoplasts of wild-type *Arabidopsis* Col-0 plants grown for 6 weeks in 8 h light/16 h darkness conditions were isolated with the tape-sandwich method (50). Suspensions of extracted protoplasts were then processed according to the polyethylene glycol method (51, 52). Transfected protoplasts were resuspended in buffer W1 (4 mM MES-KOH pH 5.7, 0.5 M mannitol, 20 mM KCl) and incubated for 8 to 24 h under constant agitation and application of low light intensity (PPFD of 25 μmol · m^−2^ · s^−1^). The YFP signal was recorded by using a Leica SP5 imaging system (Leica Microsystems) in combination with the water immersion objective lens HCX PL APO lambda blue 63.0 × 1.20 WATER UV. YFP emission was measured at 520 to 550 nm by applying an excitation wavelength of 514 nm. As a reference, chlorophyll auto-fluorescence was recorded above 700 nm.

### Extraction of Thylakoid Proteins

Fresh Arabidopsis leaves were harvested and ground in cold buffer (300 mM sucrose, 50 mM HEPES-KOH, pH 7.6, 5 mM MgCl_2_, 1 mM Na-EDTA, 1.25% [w/v] BSA, 22 mM ascorbate, 10 mM NaF, plant-specific protease inhibitor P9599 [Sigma]) by using a ball mill (Retsch) for 20 s at the highest frequency. The homogenate was filtered through one layer of miracloth (Millipore) and the collected filtrate was centrifuged for 4 min, 4000*g*, 4 °C to sediment thylakoid membranes and chloroplasts. Chloroplasts were disrupted by resuspending the pellet in hypotonic buffer (5 mM sucrose, 10 mM HEPES-KOH, pH 7.6, 5 mM MgCl_2_, 10 mM NaF, plant-specific protease inhibitor P9599). The remaining thylakoid membranes were pelleted by centrifugation for 5 min at 18000*g*, 4 °C, resuspended in storage buffer (100 mM sucrose, 10 mM HEPES-KOH, pH 7.6, 10 mM MgCl_2_, 10 mM NaF) and flash-frozen in liquid N_2_.

### Solubilization of Thylakoid Membrane Proteins and Native Gel Electrophoresis

Membrane complexes of the extracted thylakoids were solubilized by using either 1% (w/v) digitonin or 1% (w/v) dodecyl maltoside (53). Further steps of sample preparation as well as casting of large-pore blue native gels followed by gel electrophoresis under non-denaturing conditions were performed as previously described (53).

### 77 K Chlorophyll Emission Spectra Measurements

Freshly harvested Arabidopsis leaves were ground in cold buffer (300 mM sorbitol, 20 mM HEPES-KOH, pH 7.9, 2.5 mM Na-EDTA, 10 mM ammonium hydrogencarbonate, 2 mM ascorbate, 0.1% [w/v] BSA, 10 mM NaF, plant-specific protease inhibitor P9599 [Sigma]) with a ball mill (Retsch) for 20 s at highest frequency and the homogenate was then filtered through one layer of miracloth (Millipore). The chlorophyll concentration of the extract was determined according to the protocol of Porra and coworkers (54), and adjusted to 10 μg/ml chlorophyll before it was transferred to glass tubes and flash-frozen in liquid N_2_. Spectra were recorded with a Jasco FP-6500 spectrometer by selecting an excitation wavelength of 435 nm and detecting the chlorophyll fluorescence in an emission wavelength range from 600 to 800 nm. The data pitch was set to 1 nm and the scanning speed to 200 nm/min. Five emission spectra per biological replicate were measured and averaged.

### Chlorophyll Fluorescence Measurements

PSII-specific chlorophyll fluorescence was analyzed by using the pulse-amplitude-modulation (PAM) fluorometry with either Dual-PAM-100 or IMAGING-PAM (Walz) instruments. For the characterization of Arabidopsis seedlings grown on agar plates, photosynthetic performance was determined with the IMAGING-PAM system, which allows the simultaneous measurement of several selected spots per run. To record PSI-specific absorption parameters in addition to PSII-specific fluorescence signals, the Dual-PAM-100 equipped with DUAL-E and DUAL-DR measuring heads were used. Light response curves were measured to characterize the photosynthetic performances of the different Arabidopsis plant lines during the application of increasing light intensities, whereby one intact leaf per plant was used as one biological replicate. Prior to each measurement, plants were dark incubated for 20 min followed by the fixation of a selected leaf between the measuring heads and determination of the initial basic fluorescence F_0_ and the maximum fluorescence F_m_. The maximum P700 reduction was measured after a 10 s-phase of FR illumination via the application of a saturating light flash (55). Afterward, the leaf was treated with actinic light, which was increased in intensity in 2 min-intervals. Each illumination phase included the application of a saturating light flash at the end of the interval allowing the determination of the PSII- and PSI-specific, light-dependent parameters, such as the effective quantum yield of PSII, Y(II) (56), the nonphotochemical quenching NPQ (57), the photochemical quenching parameters qP and qL (58, 59), the quantum yields of regulated and nonregulated nonphotochemical quenching, Y(NPQ) and Y(NO) (59), as well as the quantum yield of PSI, Y(I), and the donor and acceptor side limitation of PSI, Y(ND) and Y(NA) (55).

For inducing and analyzing state transitions in Arabidopsis, the same Dual-PAM-100 setup was used. After an initial dark incubation of 20 min, the F_0_- and F_m_-parameters of a single leaf were measured by applying a saturating light flash. Photosynthesis was then activated by illumination with far-red and weak red light (23 μmol · m^−2^ · s^−1^) for a period of 5 min, followed by a phase of 20 min with only red-light incubation to induce state 2. Induction of state 1 was afterwards achieved by switching the far-red light source on again for an additional phase of 20 min. At the end of each 20 min-interval, a saturating light flash was given to determine the maximum fluorescence level in state 1 (F_m1_) and state 2 (F_m2_), respectively. The state transition parameters qT, qS, and IB were calculated according to the protocols of Yeates and coworkers as well as of Ruban and Johnson, whereby the measure of energy imbalance, IB, was used as an internal control to optimize the light settings of the measuring device (60, 61).

### Determination of CO_2_ Assimilation Rates

Six-week-old plants, which were adapted to GL (PPFD = 120 μmol · m^−2^ · s^−1^), were placed into the measuring chamber of a LI-6800 (LI-COR) gas exchange system and incubated in darkness with a constant CO_2_ concentration of 400 ppm. As soon as a stable CO_2_ level was reached, the actual assimilation rate was recorded and averaged over a period of 20 to 30 s. In a stepwise approach, the light intensity was increased and the CO_2_ assimilation rate was determined. The leaf area of each plant was calculated by dissecting the corresponding rosette and calculating its overall surface with the help of the image analysis software ImageJ (62). For correction of CO_2_ assimilation rates, each plant pot containing only the leftover soil was measured in the same experimental setup (63). Six plants per genotype were analyzed and differences between the plant lines were tested for significance by a One-Way ANOVA approach and a Fisher LSD test with a significance level of *p* ≤ 0.05.

### Sample Preparation for Transmission Electron Microscopy

Wild type and mutant plants were grown for 6 weeks under short-day conditions (8 h light/16 h darkness, PPFD of 120 μmol · m^−2^ · s^−1^) prior to the preparation of leaf discs. The subsequent steps of processing were then performed as described previously (27). In total, 160 grana stacks of 16 TEM images, which were derived from at least five biological replicates, were analyzed per genotype.

### Lysine Acetylome Profiling

Arabidopsis leaves were harvested in the middle of the light phase from plants, which have been grown for 6 weeks in an 8 h light/16 h darkness regime. The leaves were flash-frozen in liquid N_2_ and ground to a fine powder before proteins were extracted using a filter-assisted sample preparation protocol (64, 65). The proteins were trypsin-digested and afterwards dimethyl-labeled on C_18_ Sep-Paks plus short columns (Waters) as previously reported (66). A label swap was introduced. Equal amounts of light- and medium-labeled peptides were combined for each replicate and dried in a vacuum centrifuge. Before the enrichment of lysine-acetylated peptides, samples were resuspended in TBS buffer (50 mM Tris-HCl, pH 7.6, 150 mM NaCl). A 10 μg-peptide aliquot per sample was stored for whole proteome analysis, while the remaining peptide was treated with an anti-acetyllysine antibody coupled to agarose beads (ICP0388, Immunchem, www.immunechem.com) as described previously (64). Peptides were desalted, fractionated in three steps using SDB stage tips, and dried by vacuum centrifugation (67).

### Non-denaturating Extraction of the Leaf Proteome and Enrichment of GNAT-GFP Interacting Proteins

Arabidopsis leaf proteins were extracted in a native buffer and enriched as described in detail by Née and coworkers (68). In brief, frozen leaves were ground in extraction buffer (50 mM HEPES-KOH, pH 7.5, 150 mM NaCl, 10% [v/v] glycerol, 2 mM EDTA, 5 mM DTT, 0.5% [v/v] Triton X-100, plant-specific protease inhibitor P9599 [Sigma]) and the lysate was incubated for 1 h at 4 °C. After centrifugation, an aliquot of 20 μg protein per replicate of the soluble phase was stored for whole proteome analysis. For the pulldown analysis, 2 mg protein was mixed with 20 μl GFP-Trap A bead slurry (ChromoTek) and incubated for 2 h at 4 °C under constant agitation. The beads were washed with cold washing buffer (50 mM HEPES-KOH, pH 7.5, 150 mM NaCl, 10% [v/v] glycerol, 2 mM EDTA, 5 mM DTT) to remove non-binding proteins. At this point, the chemical cross-linker disuccinimidyl sulfoxide (DSSO) was introduced where indicated to generate covalent intra- and inter-protein bonds between neighboring lysine residues that can be cleaved later during the MS run. DSSO was added in a concentration of 12 mM and incubated for 1 h at 8 °C. Excess of DSSO was then quenched by the addition of Tris buffer in a final concentration of 20 mM. Afterwards, proteins were denatured, reduced, and alkylated on-bead as previously described (13). Proteins were digested with LysC overnight at room temperature, followed by a tryptic digest for an additional 4 h at 37°C. Peptides were then desalted using C_18_ stage tips and dried by vacuum centrifugation. Proteins of the 20 μg-total-proteome aliquots were purified from the extraction buffer and digested into peptides via the sample preparation protocol described by Hughes and coworkers using paramagnetic beads (Cytiva) (69).

### LC-MS/MS Data Acquisition

The dried peptide pellets were resuspended in 2% [v/v] ACN, and 0.1% [v/v] TFA for analysis. Samples were measured using an EASY-nLC 1200 coupled to a Q Exactive HF or an Orbitrap Exploris 480 mass spectrometer. In the case of cross-linking experiments samples were measured in a Vanquish Neo coupled to an Orbitrap Eclipse equipped with a FAIMS pro duo (all instruments Thermo Fisher Scientific, see also Supplemental Data S7). During liquid chromatography, peptides were separated on 20-cm-frit-less pulled emitters (CoAnn Technologies Inc, 0.75-μm inner diameter), packed in-house with reversed-phase ReproSil-Pur C_18_ AQ 1.9 μm resin (Dr Maisch), whereby the column was kept at 50 °C in a column oven throughout the run. The following parameters were applied for total-proteome analysis, while values stated in brackets indicate parameters used for lysine acetylome and co-immunoprecipitation analysis; if not stated separately, the parameters were identical. Peptides were eluted for 115 (68) minutes applying a segmented linear gradient of 0 to 98% solvent B (solvent A, 0% [v/v] ACN, 0.1% [v/v] FA; solvent B, 80% [v/v] ACN, 0.1% [v/v] FA) with a flow rate of 300 (250) nl/min. Mass spectra were acquired in a data-dependent acquisition mode with a Top15 method. The mass range was set to 300 to 1759 *m/z* at a resolution of 60,000 (120,000) FWHM, maximum IT of 55 ms, and a target value of 3 × 10^6^ ions. Precursors were selected within an isolation window of 1.3 (1.2) *m/z* and HCD fragmentation was performed with a normalized collision energy of 25. MS/MS spectra were acquired with a target value of 10^5^ (5 × 10^4^) ions at a resolution of 15,000 FWHM, a maximum IT of 55 (150) ms, and a fixed first mass of *m/z* 100. Peptides revealing a charge of +1, >6, or with an unassigned charge state were excluded from fragmentation for MS^2^, and dynamic exclusion for 30 s prevented repeated selection of precursors. Cross-linked peptides were measured twice using both the “XLMS Cleavable MS2 MS3” and “XLMS Cleavable MS2 MS2 MS3” method templates with 2 CVs (−50 V and −75 V) per run enabling the triggering of cross-link specific fragmentation spectra based on the specific mass shift of DSSO (31.9721 Da). Raw data (other than cross-link) were processed using MaxQuant software version 2.0.3.0 (70, 71), searching against the latest version of the Araport reference proteome for *A. thaliana* (Araport11 containing 48,266 entries; (72)). A list of common contaminants and a reverse decoy database were enabled for the search. Carbamidomethylation was used as a fixed modification and oxidation of methionine and N-terminal acetylation were set as variable modifications. In addition, lysine acetylation was enabled as variable modification in the case of lysine acetylome profiling for enriched samples. In the case of dimethyl-labeled peptides, the multiplicity of MS^1^-based quantification was set to two selecting the light and intermediate label. For processing of data derived from label-free GNAT-GFP co-enrichment approaches, the options of Label-Free Quantification (LFQ) and intensity-Based Absolute Quantification (iBAQ) were enabled. Trypsin and, if required, LysC, were selected as proteases, and maximal two missed cleavages (total-proteome, co-immunoprecipitation assay) or four missed cleavages (acetylome) were accepted. The minimum length of valid peptides was set to seven amino acids. Re-quantify and “match-between runs” was enabled. Other settings were kept as default: PSM and protein FDR was 0.01. A tolerance of peptide of 20/4.5 ppm was allowed for first/main search, while the MS/MS peak tolerance was 20 ppm. Data for cross-link detection was processed using ProteomeDiscoverer 3.0.1.27 and XlinkX (73). The Araport reference proteome for *A. thaliana* was filtered for proteins localizing to the plastid according to SUBA4 reducing the search space to 3318 entries (74). Trypsin was set as a peptidase allowing up to 2 missed cleavages. Carbamidomethylation was used as a fixed modification and oxidation of methionine and N-terminal acetylation were set as variable modifications, DSSO was used as a cross-linker. PSM, CSM, and protein FDR were 0.01. A tolerance of precursor ions of 10 ppm, FTMS Fragment Mass Tolerance of 20 ppm, and ITMS Fragment Tolerance of 0.5 Da were set. MS raw data were uploaded to the JPOST repository (identifier: JPST002262). In addition, spectra were also available via MS-viewer under sc2f0nxgso (for the co-immunoprecipitation profiles) and sjczk1ixdg (for the lysine acetylation profiles).

### Data Analysis

Downstream data analyses were performed using Perseus version 1.6.15.0 (75) and R version 4.1.1 in combination with the additional LIMMA statistics package (76, 77). Prior to differential expression or enrichment analyses, reverse hits and potential contaminants were removed. For the co-immunoprecipitation experiments, valid quantifications in at least three of four biological replicates per experiment (genotype) were required and in addition, significantly enriched protein groups had to possess a log_2_ fold change of ≥1 and a false discovery rate-corrected *p*-value ≤0.05 (LIMMA). Figures were prepared with the help of the software tool OriginPro version 2022b (OriginLab Corporation).

### MS-Based Characterization of Protein N-Termini and NTA Profiling

N-terminome analysis was performed on Arabidopsis leaves from the same plants that were harvested for the lysine acetylome profiling. These leaves were frozen in liquid nitrogen and ground before being processed following the SILProNAQ protocol (78). The powder was solubilized in a specific buffer for protein extraction (50 mM HEPES-NaOH pH 7.2, 1.5 mM MgCl_2_, 1 mM EGTA, 10% [v/v] glycerol, 1% [v/v] Triton X-100, 2 mM PMSF, 150 mM NaCl, protease inhibitor cocktail tablet). Protein amounts were determined by Bradford assays and 1 mg of protein was used for each sample. After denaturation of the proteins by reduction and subsequent alkylation of cysteine residues, proteins were N-terminally labeled with d3-N-Acetoxysuccinimide, before being digested with trypsin. Peptides were desalted using C_18_ SepPak cartridges and vacuum dried. Dried peptides were resuspended in 5 mM KH_2_PO_4_, 30% [v/v] ACN, 0.05% [v/v] FA (SCX buffer A) and loaded onto a Polysulfoethyl A column (polyLC, 2.1 × 200 mm, 5 μm, 200 Å). The elution was performed using a multi-step gradient of 5 mM KH_2_PO_4_, 350 mM KCl, 30% [v/v] ACN, 0.05% [v/v] FA (SCX buffer B) as follows: 0% B for 5 min; 0 to 26% B in 10 min; 26 to 35% B in 25 min; 35 to 60% B in 5 min; 100% B for 10 min; 0% B for 20 min. Fractions were collected every 2 min, for 30 min, at a constant flow rate of 0.2 ml/min. The fractions 2 to 11 were kept for analysis and vacuum-dried. The dried SCX fractions were resuspended in 30 μl of 2% [v/v] ACN, 0.1% [v/v] TFA each, before being loaded individually on an EASY-nLC 1000 coupled to an LTQ-Orbitrap Velos mass spectrometer (Thermo Fisher Scientific). The peptide separation was done using a 15 cm C_18_ capillary column (NTCC-360/100-5-153, Nikkyo Technos Co., Ltd) and a 55 min method that allows for fragmentation of singly charged ions. The MS full scans were set for an m/z range of 400 to 2000 Th, with a 60,000 FWHM resolving power. The 20 most intense ions were selected each time for CID fragmentation. With the chromatography buffers being 2% [v/v] ACN, 0.1% [v/v] FA (buffer A) and 100% [v/v] ACN, 0.1% [v/v] FA (buffer B), the separation was done as follows, with a constant flow rate of 300 nl/min: 5 to 35% B in 42 min; 35 to 80% B in 1 min; 80% B for 12 min. Raw data files from a sample’s fractions were processed together using Mascot Distiller version 2.6.2 (Matrix Science) to search against the Araport11 database (48,266 entries (72) with the mass tolerances respectively set to 10 ppm and 0.5 Da for parent and fragment ions, and cysteine carbamidomethylation and lysine d3-acetylation as fixed modifications, methionine oxidation as a variable modification. The enzyme was defined as Semi-Trypsin/P and up to 5 miss cleavages were allowed to account for incomplete trypsin digestion that can occur with acetylated lysine residues. The NTA was quantified by selecting the “Acetylation” quantitation method. Identification and quantitation results were exported from Mascot and parsed through the eNcounter script (26, 78). Thereby, the peptide ion score was set to 25 and the significance threshold to 0.2. These values were adjusted to a score of 30 and an e-value of 0.05 by the eNcounter tool (79). Further data processing was done in Microsoft Excel using the built-in statistics tools.

### Experimental Design and Statistical Rationale

We sampled material from four individual plants for MS analysis and processed them with no additional technical replicates. Detailed statistical and bioinformatic analyses of proteomics data are described in the respective sections above. The respective tests as well as p-value- and FDR-cutoffs are indicated within the respective figure legends. Supplemental Data S7 outlines the respective instruments used to generate the data.

## Results

### Recombinant His_6_-GNAT1 Shows a Relaxed Substrate Specificity *In Vitro*

**Fig. 1:**
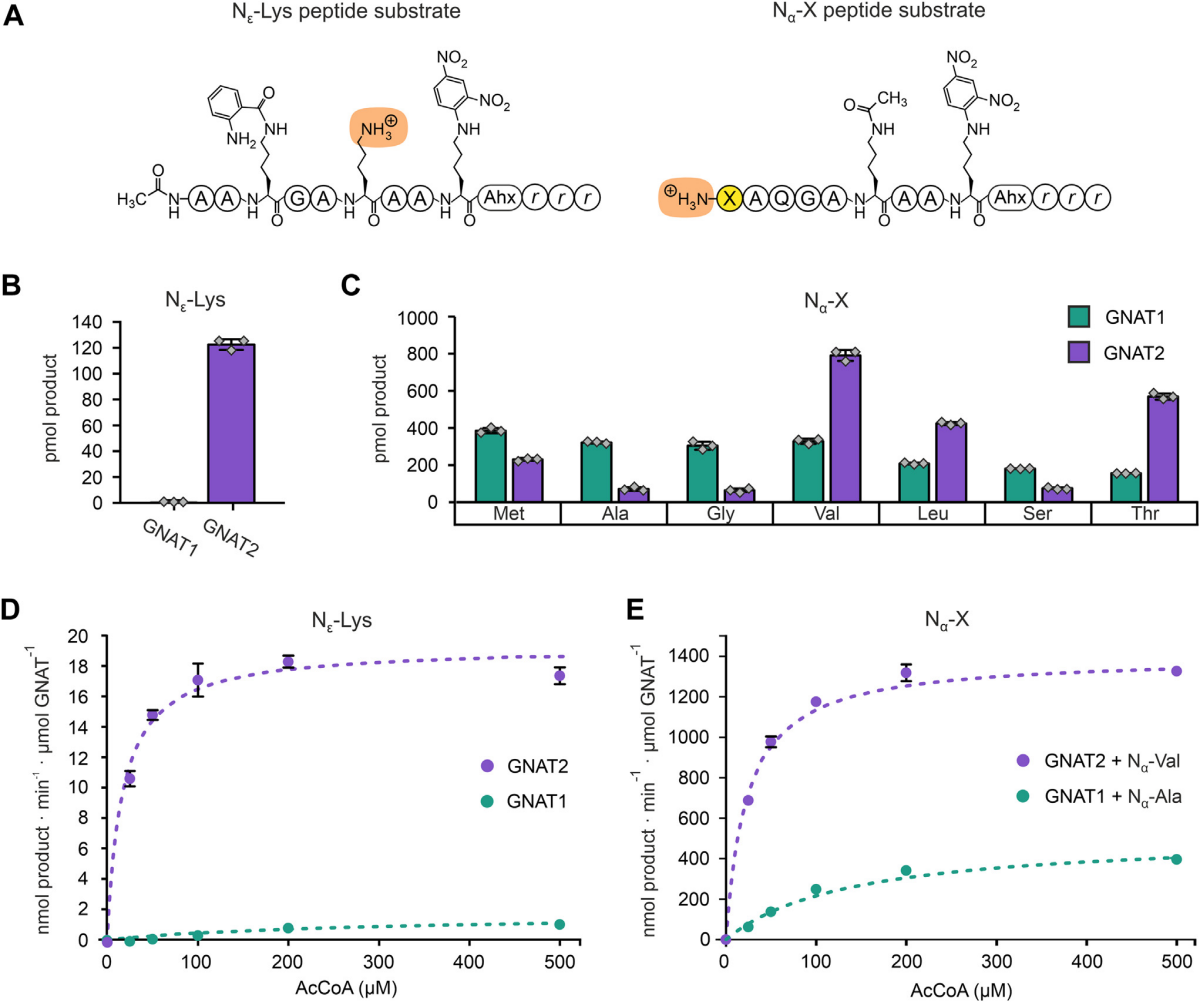
**Biochemical characterization of recombinant His**_**6**_**-GNAT1 and His**_**6**_**-GNAT2 reveals dual lysine and N-terminal acetylation activities.***A*, synthetic substrate peptides used for N_ε_-Lysine (KA) and N_α_-N-terminus acetylation (NTA) assays in an HPLC-based set-up. The introduced dinitroaniline group allows a specific detection of the peptides at a wavelength of 360 nm. The amino acid residues were labeled in one-letter code. Ahx refers to 6-aminohexanoic acid and *r* to D-arginine. *B*, KA and (*C*) NTA (Met, Ala, Gly, Val, Leu, Ser, Thr) activities of recombinant His_6_-GNAT1 and His_6_-GNAT2. The activities were determined by end-point analysis of the acetylated fraction that was generated with the different synthetic substrate peptides after 4 h (*B*) and 1 h (*C*), respectively. The recombinant GNATs (2 μM) were incubated with the synthetic peptide substrate (100 μM) and the reaction was started by the addition of 200 μM acetyl-CoA at 30 °C. A fraction corresponding to 1000 pmol of peptide was analyzed by HPLC. The bar graphs (*B* and *C*) represent the final fraction of acetylated peptide (product) generated by 20 pmol of the recombinant GNAT (n = 3, ±SD). *Grey* rectangles show the individual activities of each replicate. Michaelis-Menten kinetics of the KA (*D*) and the NTA activity (*E*) were performed with His_6_-GNAT1 and His_6_-GNAT2, respectively. The catalytic activities were determined with increasing concentrations of acetyl-CoA (0, 25, 50, 100, 200, 500 μM) and the resulting curves were fitted according to the Michaelis-Menten function f(x) = V_max_ ∙ x ∙ (K_m_ + x)^−1^. A fixed peptide substrate concentration of 100 μM was used (n = 3, ±SD).

Based on their phylogenetic relationship, GNAT1, 2, and 3 were previously assigned to a distinct subgroup of plastidial NATs called NAA90 (25, 26). Since GNAT1 and GNAT2 showed the highest degree of proximity within the phylogenetic tree, we first compared the *in vitro* substrate preferences of the recombinant GNAT proteins in acetyltransferase enzyme assays. For these *in vitro* assays, synthetic peptides were used, which are specifically designed to measure dual acetyltransferase activities in an established HPLC-based assay (Fig. 1*A*; (26, 27, 40)). The peptides share a nearly identical amino acid sequence and contain either a free N-terminus with varying amino acid residues (Ala, Gly, Ser, Thr, Val, Met, or Leu) or a free lysine side chain as a substrate for acetylation. In addition, the peptides carry a dinitroaniline side chain close to the C-terminus to specifically record their absorbance at 360 nm during HPLC analysis. The recombinant N-terminally His_6_-tagged GNAT1 and GNAT2 proteins lacking the predicted plastid transit peptides were expressed and purified from *E. coli* (Supplemental Fig. S1). For the comparison of their catalytic activities, we used fixed substrate (100 μM peptide, 200 μM acetyl-CoA) and protein concentrations (2 μM) in an end-point enzyme assay. GNAT1 exhibited almost no activity using the KA substrate peptide even after 4 h of incubation, while GNAT2 showed a considerable lysine acetylation activity as previously reported (Fig. 1*B*; (26, 27)). The substrate preference of GNAT1 for different peptide N-termini was slightly different from that of GNAT2. While GNAT2 showed the highest activity on N-terminal Val residues, followed by Thr and Leu, GNAT1 exhibited its highest activity on N-terminal non-polar amino acids, such as Met, Ala, Gly, or Val (Fig. 1*C*). Still, both enzymes were able to acetylate all provided NTA substrates. To compare their enzymatic properties, we determined the acetyl-CoA-dependent Michaelis-Menten kinetics for both GNATs on the lysine peptide as well as on one of their preferred N-terminus peptides. The Michaelis-Menten kinetics revealed a low KA activity for GNAT1, which was enhanced with increasing concentrations of acetyl-CoA and resulted in a maximum velocity V_max_/[E] of 1.7 ± 0.3 nmol product ∙ min^−1^ ∙ μmol enzyme^−1^. GNAT2 in contrast, exhibited a more than 10-fold higher activity with a V_max_/[E] of 19.3 ± 0.4 nmol product ∙ min^−1^ ∙ μmol enzyme^−1^ (Fig. 1*D* and Table 1). Moreover, GNAT2 had a lower K_m_-value (K_m_ = 16.9 ± 1.9 μM) for acetyl-CoA in combination with the KA peptide than GNAT1 (K_m_ = 302.1 ± 89.1 μM) indicating a higher affinity for acetyl-CoA in GNAT2. When using one of the preferred peptide substrates for NTA, such as N_α_-Ala, Michaelis-Menten parameters were measured for GNAT1 with a V_max_/[E] of 515.7 ± 14.8 nmol product ∙ min^-1^ ∙ μmol enzyme^-1^ and a K_m_ of 137.6 ± 9.8 μM (Fig. 1 E). Also, GNAT2 exhibited a distinct saturation curve profile on an NTA substrate peptide, here consisting of an N-terminal valine residue, with an almost three-fold higher V_max_/[E] of 1400.7 ± 3.0 nmol product ∙ min^−1^ ∙ μmol enzyme^−1^ than GNAT1 and a K_m_ of 23.4 ± 0.3 μM. The V_max_/[E]-values for both enzymes underline the preference of both enzymes for the NTA peptide probes and underpin that GNAT2 has a higher catalytic KA and NTA activity than GNAT1 in the course of specific enzyme kinetics measurements (Table 1).

**Table 1:** Enzymatic properties of recombinant His_6_-GNAT1 and His_6_-GNAT2

Catalytic parameters	KA activity N_ε_-Lys peptide substrate		NTA activity N_α_-X peptide substrate	
	GNAT1	GNAT2	GNAT1 (N_α_-Ala)	GNAT2 (N_α_-Val)
V_max_ (nmol∙min^−1^ μmol GNAT^-1^)	1.74 ± 0.28	19.26 ± 0.43	515.7 ± 14.8	1400.7 ± 3.0
K_m_ (μM)	302.1 ± 89.1	16.9 ± 1.9	137.6 ± 9.8	23.44 ± 0.32
k_cat_ (min^-1^)	0.002 ± 0.0003	0.019 ± 0.0004	0.515 ± 0.015	1.401 ± 0.003

The catalytic activities were measured with increasing concentrations of acetyl-CoA (0, 25, 50, 100, 200, 500 μM) and a fixed peptide substrate concentration of 100 μM (n = 3). The resulting curves were fitted according to the Michaelis-Menten function f(x) = V_max_ ∙ x ∙ (K_m_ + x)^−1^ and the parameters V_max_, K_m_, and k_cat_ are shown as averages ± SD.

### Predicted GNAT1 and GNAT2 Protein Structures Exhibit a High Degree of Similarity in the Architecture of Their Functional Acetyltransferase Domains

**Fig. 2:**
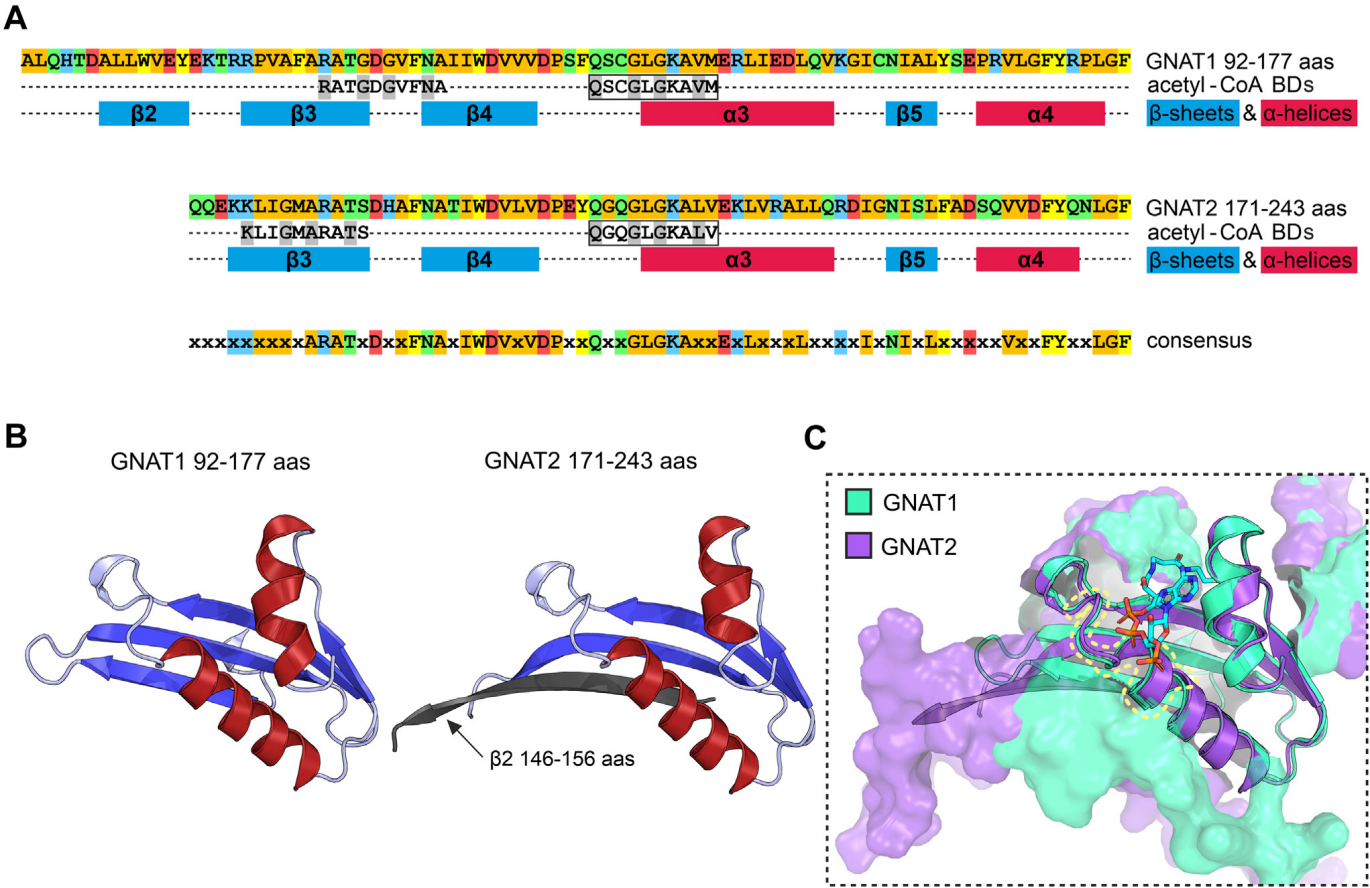
**The predicted structures of GNAT1 and GNAT2 acetyltransferase domains are highly similar.***A*, schematic overview of primary and secondary structures of GNAT1 and GNAT2 centered on the region annotated as acetyltransferase Pfam domain (PF00583, aligned region; Finn *et al.*, 2010). The colors, which highlight different amino acid residues displayed as one-letter code, represent the side chain features as follows: *orange*, non-polar; *yellow*, *aromatic*; *green*, polar (neutral); *blue*, positively charged, and *red*, negatively charged. The regions of acetyl-CoA binding are displayed below the primary amino acid sequence with the boxed region representing the main binding site (Bienvenut *et al.*, 2020). In the third row, secondary structure elements determined by AlphaFold 2 structure prediction were marked by *red* (α-helix) or *blue* (β-sheet) boxes (Jumper *et al.*, 2021). The consensus sequence combining the primary structure features of GNAT1 and GNAT2 is shown below. Amino acid residues, which are identical between both acetyltransferases, are shown in one-letter code, while differing amino acids in corresponding positions were replaced by an 'x'. *B*, AlphaFold 2 structure predictions for the acetyltransferase domains of GNAT1 and GNAT2. Protein structure data were obtained from the AlphaFold 2 database and visualized using PyMOL (The PyMOL Molecular Graphics System, Version 4.5 Schrödinger, LLC). *C*, superimposition of the predicted acetyltransferase domain models presented in (*B*). Protein structure predictions of the AlphaFold 2 platform were extended by integration of the cofactor coenzyme A (CoA) as a *stick* model with the help of the AlphaFill tool and a superimposition of both GNATs was performed using PyMOL (Hekkelman *et al.*, 2021). The *dashed yellow line* highlights the main acetyl-CoA binding site of both GNATs.

GNAT1 and 2 show the highest phylogenetic relationship among the plastidial GNATs and reveal a significant amino acid sequence conservation (41% sequence identity as assigned with PRALINE Multiple Sequence Alignment tool, Supplemental Fig. S2). To compare the catalytic sites of both enzymes we used the Pfam (Protein families) acetyltransferase domains as a basis (80). Most notably, regions of the β3-and the α3-motif, as well as two patches in between, are consistently characterized by a majority of non-polar amino acid residues. These residues form mainly groups of two or three amino acids flanking the highly conserved sites of polar residues (Fig. 2*A*). As there is no experimentally determined 3D structure available, AlphaFold 2 predicted models of mature GNAT1 and GNAT2 amino acid sequences were generated for tertiary structure comparisons. These predictions reveal an identical organization of the secondary structure motives belonging to the respective acetyltransferase domain (Fig. 2, *A* and *B*). The state-of-art computational method provided by AlphaFold 2 allows highly accurate protein structure modeling without necessarily requiring experimentally validated reference structures and serves as an excellent basis for additional tools such as AlphaFill, which even enables the *in silico* incorporation of ligand molecules into the predicted structure models (41, 42). By taking advantage of both methods and extending the computed protein structures of GNAT1 and GNAT2 by the structure model of their cofactor coenzyme A, respectively, a superimposition of both GNAT models was achieved centered on the cofactor. The identical architecture of the catalytic domains of GNAT1 and GNAT2 including the complete conformity of the primary acetyl-CoA binding sites can be derived as an obvious characteristic (Fig. 2*C*). These models elucidate the observed similarities in the catalytic activities of both enzymes but do not offer insights into their differences. Those might be due to differences in their structures outside of the catalytic domain.

### GNAT1 Regulates a Variety of Acetylation Sites on Plastidial Proteins as Identified by *In Vivo* Lysine and N-terminus Acetylome Profiling

**Fig. 3:**
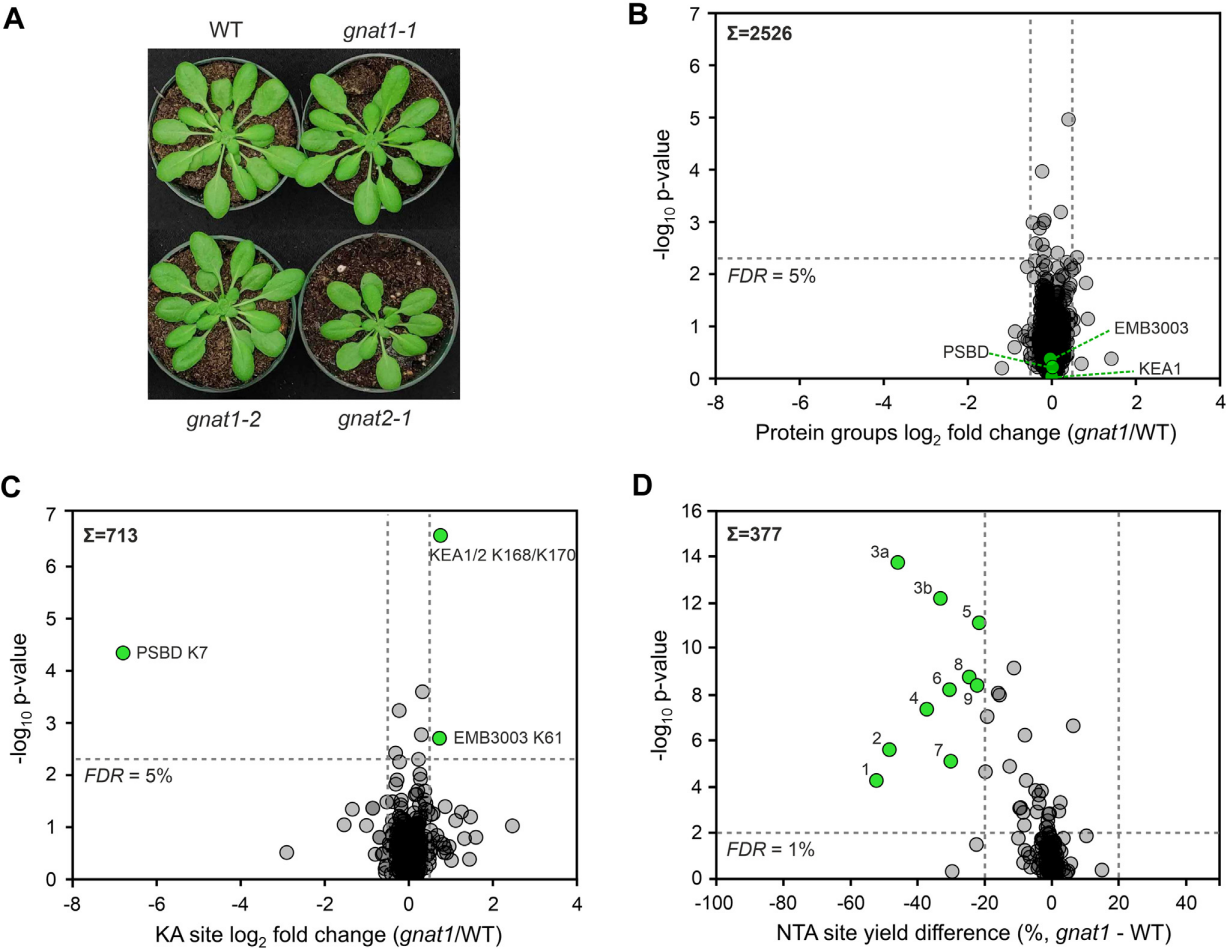
**GNAT1 regulates a variety of lysine and N-terminal acetylation sites but shows no obvious growth phenotype.***A*, Phenotype of *gnat1* mutant lines in comparison to the wild type (WT) and the GNAT2-deficient knockout line *gnat2-1*. Plants were cultivated under short-day conditions (8 h light/16 h dark) with PPDF = 120 μmol ∙ m^−2^ ∙ s^−1^, humidity = 50% and temperature = 22 °C for six to 7 weeks. Protein abundance (*B*), lysine acetylation KA (*C*), and N-terminal acetylation NTA (*D*) proteomic profiles displaying up- or downregulation in GNAT1 deficient plants in comparison to the WT. Sums indicate the total numbers of quantified protein groups or acetylation sites. For statistical evaluation of (*B* and *C*), the lines *gnat1-1* and *gnat1*-*2* were treated as one group and per protein group or protein site, a quantifiable identification in at least three out of eight biological replicates was required to be considered as valid. Altogether, four biological replicates of *gnat1-1* and of *gnat1-2*, respectively, and eight replicates of WT plants were processed, amounting to a number of four combined *gnat1-1*/WT and four combined *gnat1-2*/WT samples. *Green circles* highlight plastid localized proteins, which show significant up- or downregulation in abundance or acetylation of a specific site (log_2_ fold change ≤ −0.5 or ≥ 0.5 and false discovery rate-corrected *p*-value ≤0.05 [LIMMA]). For the investigation of the NTA profile of *gnat1* (*D*), the lines *gnat1-1* and *gnat1-2* were treated as one group, and the WT profile was derived by combining the data obtained in this work and previously published data set (Bienvenut *et al.*, 2020; Eirich *et al.*, 2024). For the experimental work that was performed in the course of the present study, three biological replicates of *gnat1-1*, of *gnat1-2*, and of WT plants, respectively, were processed. Significantly up- or downregulated N-termini that originate from proteins with plastid localization are marked by green color. The NTA differences between *gnat1* and WT were analyzed with GraphPad 10 and plotted as yield difference in % (*gnat1* - WT). An unpaired *t* test analysis with FDR 1% was performed and N-termini displaying >20% difference in their NTA yield were regarded as significant. 1. THIC (T55, AT2G29630) 2. SYNO (T60, AT4G17300) 3a. F16P1 (V61, AT3G54050) 3b. F16P1 (A60, T3G54050) 4. ZHD10 (A72, AT2G21530) 5. ZEP (A61, AT5G67030) 6. PORC (T69, AT1G03630) 7. NAGK (T51, AT3G57560) 8. AT2G14880 (T46) 9. MORF9 (T61, AT1G11430).

To investigate the *in vivo* substrates of GNAT1 and to compare them to the previously identified substrates of GNAT2, we isolated two independent homozygous T-DNA insertion lines from the Arabidopsis stock centers (*gnat1-1*, *gnat1-2*; Supplemental Figs. S3 and S4). Both *gnat1* lines showed no obvious growth phenotype compared to the wild type (WT), while the *gnat2-1* mutant exhibited delayed growth as reported previously (Fig. 3 A; Table 2 (27, 31, 81). Furthermore, each of the *gnat1* lines carries a single or a double T-DNA insertion within the first 100 bp of the exon region of the *GNAT1* gene locus (AT1G26220), as determined by sequencing of the T-DNA borders (Supplemental Fig. S3). Despite the verification of the homozygous T-DNA incorporation into the *GNAT1* gene, a trimmed GNAT1 mRNA transcript was identified by Reverse Transcriptase-PCR for both mutant lines (Supplemental Fig. S4). This truncated transcript would lack an intact plastidial targeting sequence in case it would be translated in frame. Hence, the identified *gnat1* T-DNA lines are both treated as KO lines for the plastidial GNAT1 protein. For proteome as well as KA and NTA analyses, we used whole rosettes of both *gnat1* T-DNA lines and of WT plants harvested 4 h after the onset of light. All plants were grown for 6 to 7 weeks under short-day conditions before four to eight biological replicates were collected (*gnat1-1* and *gnat1-2* in four replicates, each, and WT in eight replicates). For proteome and KA profiling, the proteins were extracted, trypsinated, and the peptides dimethyl-labeled with light and medium labels. A label swap of light and medium dimethyl-labels was introduced for two of the four *gnat1* sample replicates, respectively, and for four of the eight WT sample replicates. Differentially labeled WT and mutant peptide samples from each biological replicate were pooled for subsequent analyses. In the samples used for the quantitative proteome profiling, 5302 protein groups were identified with 2625 protein groups that were quantified in at least three biological replicates of eight replicates in total. To reveal significant differences, which are consistent in both *gnat1* mutant lines, both lines were treated as one sample group in the LIMMA statistical analyses. No significant differences in protein abundances were observed between WT and the *gnat1* mutants (Fig. 3*B* and Supplemental Data S2). In the course of the KA analyses, the pooled peptides were enriched using an acetyllysine antibody coupled to agarose beads before LC-MS/MS. From 912 identified KA sites, 713 were quantified in at least three from eight biological replicates and used for further downstream analyses. In the end, only 1 KA site was identified as significantly downregulated in the absence of GNAT1 in both mutants in comparison to the WT plants, but this site revealed a remarkable decrease in its acetylation abundance by a factor of more than a hundred-fold (Fig. 3*C*, Supplemental Table S1, and Supplemental Data S1). The affected protein is the chloroplast-encoded PSII subunit PSBD (D2) that harbors the identified KA residue in its N-terminal domain (K7). Together with cytochrome b_559_, PSBD forms the first subcomplex unit in the process of *de novo* PSII assembly, thereby providing the basic structure for binding and arranging additional subunits as well as redox-active cofactors (82). In the mature PSII complex, a heterodimer composed of PSBD and PSBA (D1) represents the central reaction center core, which coordinates the reactive chlorophyll *a* pair of PSII (83). Interestingly, the KA-site that has been previously identified as the most downregulated KA site in the *gnat2* acetylome profile was also found in the acetylome of the *gnat1* mutants (27). It can be assigned to the plastid inner envelope potassium transporters KEA1 and KEA2 (K168/K170) and in *gnat1*, it showed a slight but significant upregulation in KA in comparison to the WT (Fig. 3*C*, Supplemental Table S1, and Supplemental Data S1). This finding might hint at a compensatory GNAT2 activity in the absence of GNAT1. The overall protein abundances of the PSBD subunit as well as of KEA1 and KEA2 were not altered between mutant and WT plants (see Fig. 3*B*, Supplemental Table S1, and Supplemental Data S2).

**Table 2:** Physiological and photosynthesis-related parameters investigated for the wild type and the mutant lines *gnat1-1*, *gnat1-2*, and *gnat2*

Plant line	Dry weight (mg)	Chl *a*+*b* (μg/mg)	Chl *a*/*b*	F_v_/F_m_	P_m_	qT	qS
Wild type	59.9 ± 14.2^a^	1.02 ± 0.11^a^	3.64 ± 0.14^a^	0.81 ± 0.01^a^	0.84 ± 0.10^a^	0.11 ± 0.01^a^	0.91 ± 0.01^a^
*gnat1-1*	66.2 ± 14.6^a^	1.02 ± 0.06^a^	3.59 ± 0.15^a^	0.81 ± 0.01^a^	0.94 ± 0.09^bc^	0.10 ± 0.01^a^	0.91 ± 0.01^a^
*gnat1-2*	64.9 ± 14.7^a^	1.02 ± 0.09^a^	3.55 ± 0.12^ab^	0.81 ± 0.02^a^	0.91 ± 0.09^ab^	0.11 ± 0.01^a^	0.91 ± 0.02^a^
*gnat2-1*	45.3 ± 13.2^b^	1.01 ± 0.11^a^	3.37 ± 0.13^b^	0.81 ± 0.01^a^	1.02 ± 0.06^c^	0.00 ± 0.01^b^	0.28 ± 0.02^b^

Differences between genotypes were tested with ANOVA and for multiple comparisons, Fisher’s LSD test was applied (n = 6). F_v_/F_m_ = (F_m_ -F_0_)/F_m_, qT = (F_m1_ - F_m2_)/F_m1_, and qS = (F_S1′_ - F_S2′_)/(F_S1′_ - F_S2_).

Different letters indicate significant differences (ANOVA post-hoc Fisher LSD *p* ≤ 0.05).

To identify the *in vivo* NTA substrates of GNAT1, samples of three biological replicates per genotype were processed according to the SILProNAQ protocol (78). Analyses of WT and *gnat1* resulted in the identification of 14,359 N-termini corresponding to 1482 non-redundant proteoforms (Supplemental Data S3). Of these proteoforms, 633 were quantified with respect to their N-terminal acetylation yield (489 in WT as well as 498 and 493 in *gnat1-1* and *gnat1-2*, respectively). In contrast to KA, the N-terminus acetylome of both mutant plant lines revealed a higher number of peptides with de-regulated NTA compared to WT. Since the two *gnat1* KO mutants exhibited similar overall profiles, we combined the data from both mutants (Fig. 3*D*, Supplemental Table S2, and Supplemental Data S3). Moreover, as no major differences to the previously published WT data sets were found, we also decided to combine the WT data for a more robust and statistically relevant interpretation as well as WT and *gnat2* data from another publication for more in-depth comparisons of the *gnat* mutants (Supplemental Data S8; (26, 84)). Out of 377 quantified N-termini, nine proteins were significantly decreased in their NTA yield in the *gnat1* background (*i.e.* > 20% decrease; Fig. 3*D* and Supplemental Table S2). Interestingly, one of these proteins, the Fructose-1,6-bisphosphatase 1 (F16P1), revealed acetylation of two contiguous neo-N-termini starting with different amino acids. Altogether, these nine proteins were predicted to be plastid-localized and their function was linked to processes such as the Calvin-Benson cycle, chlorophyll, vitamin or amino acid metabolisms as well as protein biosynthesis (Supplemental Table S2 and Supplemental Data S3). The strongest decrease of NTA was found for the Phosphomethylpyrimidine synthase THIC (from a yield of 88.1% in the WT to 35.8% in the *gnat1* mutant resulting in an NTA yield difference of −52.3%), but acetylation was still detectable in the mutant background. The most remarkable decrease was observed for the N-terminus of a plastidial forkhead-domain protein (ZHD10; Supplemental Table S2 and Supplemental Data S3), whose level of NTA was nearly abolished (from a yield of 38.9% in the WT to 1.6% in the *gnat1* mutant resulting in an NTA yield difference of −37.3%). Other affected proteins included the Asparagine tRNA ligase SYNO, the Fructose-1,6-bisphospatase 1 (F16P1), the Protochlorophyllide reductase C (PORC), or the Zeaxanthin epoxidase (ZEP), which were downregulated in NTA in a range from −48.5 to −21.7% (NTA yield differences in %, *gnat1*-WT).

Next, we compared the N-termini that were mostly affected in *gnat1* with those identified in *gnat2* (26, 84). The sites with significantly decreased NTA in *gnat1* were also found in *gnat2* to be less acetylated, whereby the effect was at least similar or often even stronger in *gnat2* (Supplemental Fig. S5, *A* and *B*). Interestingly, a distinct number of additional proteins, such as High Chlorophyll Fluorescence Phenotype 244 (HC244), the chloroplast Translocon protein TIC55, the NDH-subunit PNSB2, the Argininosuccinate synthase ASSY, and the Light-harvesting complex protein LHCA5 showed differentially acetylated N-termini only in the *gnat2* plants and revealed a severely reduced acetylation yield or even a full loss of NTA in comparison to the WT (Supplemental Fig. S5, *A* and *B* and Supplemental Tables S2 and S3). We therefore concluded that the spectrum of target sites attributed to GNAT1 is mainly a subset of target sites subjected to NTA by GNAT2. However, the NTA analyses also show that GNAT2 is not able to fully compensate for the loss of GNAT1 in the *gnat1* plants (Fig. 3*D*). Moreover, the data indicate that GNAT1 supports the overall GNAT2 activity.

### gnat1 Mutants Reveal a More Compact Thylakoid Stacking but no Defects in State Transitions

**Fig. 4:**
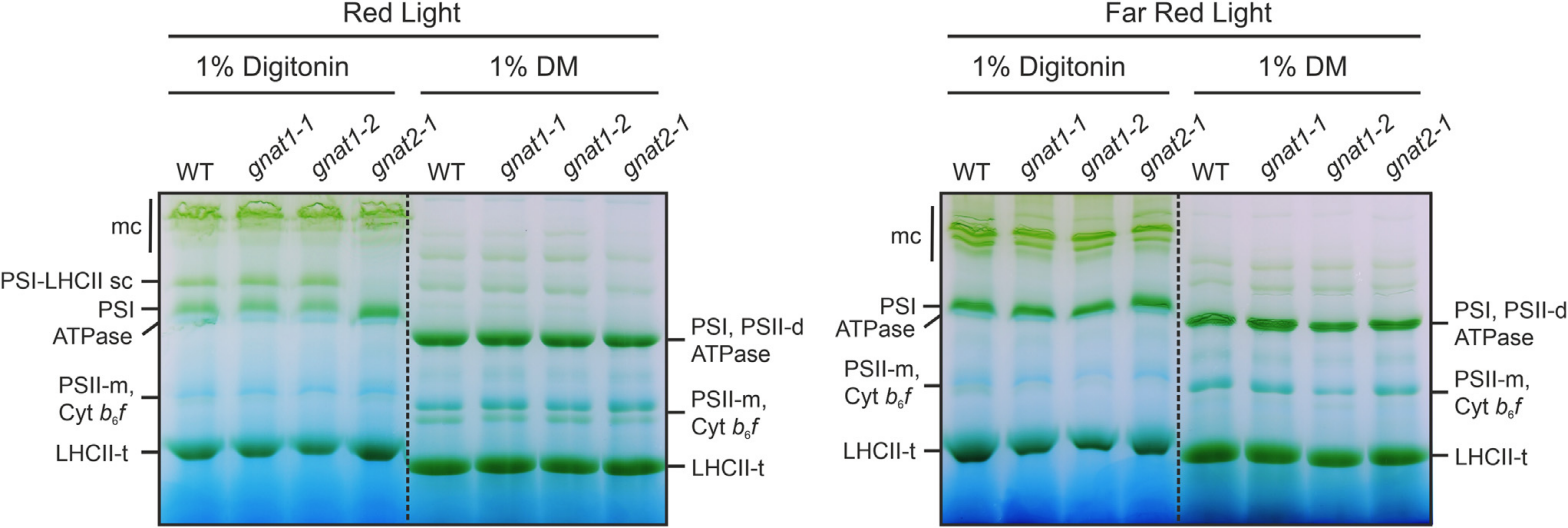
***gnat1* mutant plants are able to perform state transitions.** Large-pore blue native gel (lpBN-PAGE) images of thylakoid protein complexes isolated from Arabidopsis *gnat1-1*, *gnat1-2*, *gnat2-1*, and wild type (WT) plants. GL (PPFD = 120 μmol m^−2^ s^−1^) adapted plants were either treated with *red* (PPFD = 53 μmol m^−2^ s^−1^) or far-red light (PPFD = 30 μmol m^−2^ s^−1^) for at least 1 h before thylakoid membranes were extracted and subsequently solubilized with 1% digitonin or β-dodecylmaltoside (DM). The solubilized protein complexes were separated by lpBN-PAGEs under native conditions. mc, megacomplex; sc, supercomplex; t, trimer; d, dimer; m, monomer.

**Fig. 5:**
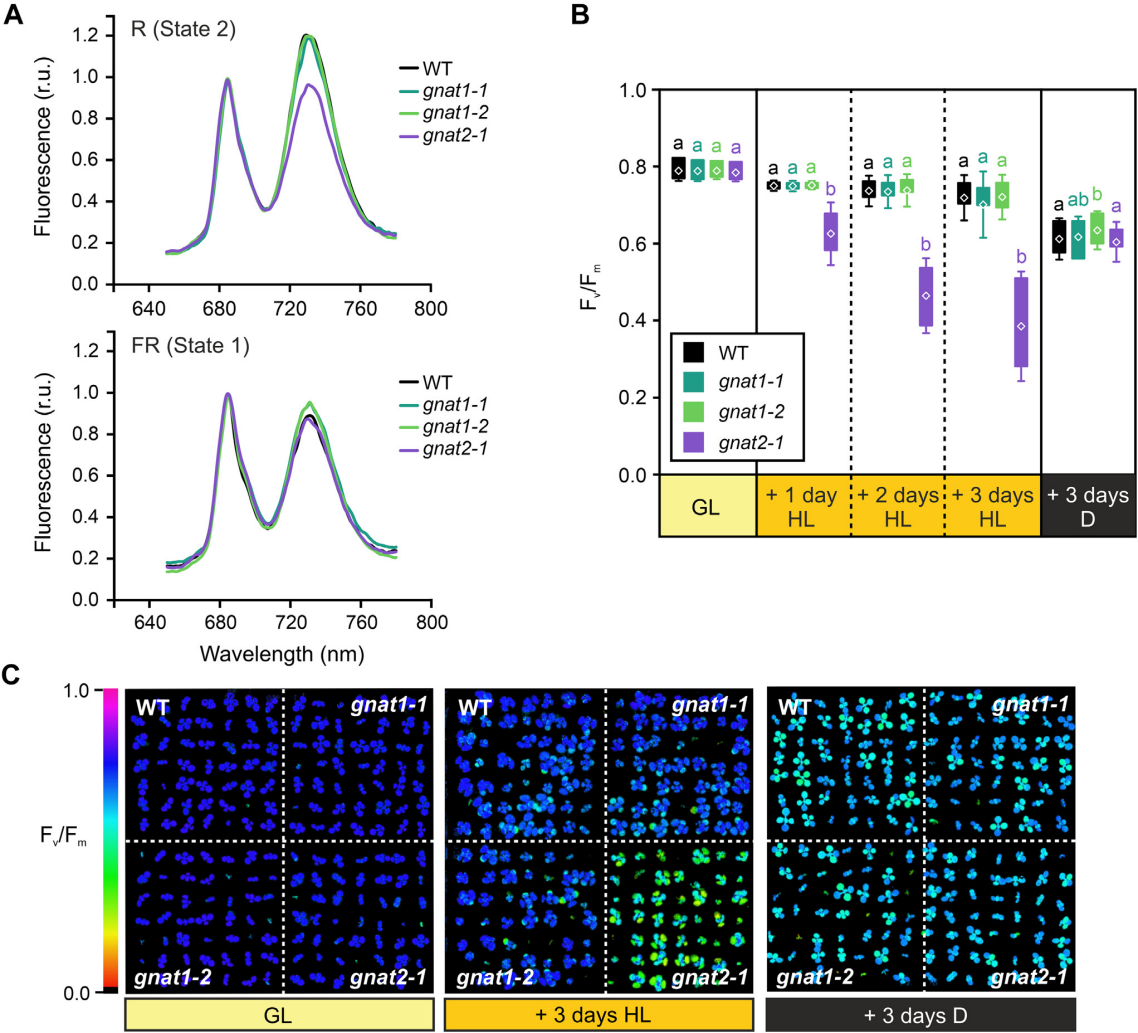
**Phenotyping of *gnat1* and *gnat2* mutants after high light treatment.***A*, 77K chlorophyll fluorescence emission spectra displaying the chlorophyll emission signal of crude leaf extracts after red (R, state 2) and far-red light treatment (FR, state 1) of *gnat1-1*, *gnat1-2*, *gnat2-1*, and wild type (WT) plants. Spectra were normalized to the fluorescence peak at 685 nm and represent the average of three replicates per genotype. The fluorescence maximum around 685 nm originates from chlorophyll molecules associated with PSII, whereas the second maximum at 735 nm is the result of PSI-specific chlorophyll fluorescence emission. *B*, maximum quantum yields (F_v_/F_m_) determined for *gnat1-1*, *gnat1-2*, *gnat2-1*, and WT seedlings treated with standard growth light (GL, PPDF = 120 μmol ∙ m^−2^ ∙ s^−1^), continuous high light (HL, PPDF = 800 μmol ∙ m^−2^ ∙ s^−1^) or darkness for the indicated number of days. Plants were grown on plates containing sterile media for 8 days under long-day conditions (16 h light/8 h darkness, PPDF = 120 μmol ∙ m^−2^ ∙ s^−1^) before the treatment. Photosynthetic performances of the seedlings were analyzed by using the Imaging-PAM system (Walz) and differences between genotypes were tested for significance by One-Way ANOVA applying a Fisher LSD test with a significance level of *p* ≤ 0.05. *C*, exemplary images of the analyzed seedlings grown on sterile media that were colored in relation to the maximum quantum yields (F_v_/F_m_). These were determined after the indicated treatments. The correlation between colors and numerical values is predefined by the Imaging-PAM software and ranges from minimal F_v_/F_m_ (0, *black*) to maximal F_v_/F_m_ (1, *magenta*).

Since several proteins of the photosynthetic machinery were affected in their acetylation status in *gnat1* mutants compared to WT, we performed further physiological characterizations. No significant differences were observed concerning dry weight per plant, chlorophyll (Chl) content, and Chl *a/b* ratio, as well as basic parameters linked to photosynthesis efficiency, such as the maximum quantum yield of PSII (F_v_/F_m_), and the amplitude of PSI oxidation (P_m_) (Table 2 and Supplemental Table S5). On average, *gnat1* rosettes showed a slightly higher biomass compared to WT. However, this difference was not significant due to the high variance among individual plants (n = 6), but similar observations have been made before on the analysis of a single *gnat1-2* line in a previous study (81). On the contrary, *gnat2-1* exhibited a significantly lower dry weight compared to WT and *gnat1* plants, a higher amplitude of P_m_, and a slightly lower Chl *a/b* ratio, whereby the latter was already previously reported (27, 31, 81). Since *gnat2* plants are not capable of performing photosynthetic state transitions as previously reported, and GNAT1 and GNAT2 share similar protein substrates (Fig. 1, *B* and *C*), we were interested to see whether *gnat1* mutants also show some deficiency in state transitioning (27, 31, 32, 81). No defect in the formation of the PSI-LHCII state transition complex was observed in *gnat1* and WT thylakoids that were treated with red light to induce state 2 and solubilized with 1% digitonin before lpBN-PAGE analysis (Fig. 4). In contrast, the absence of the PSI-LHCII complex in thylakoids extracted from *gnat2-1* could be clearly recognized. The state-specific association of the LHCII antennae was additionally analyzed by recording 77K chlorophyll emission spectra of crude leaf extracts (Fig. 5*A*). After red light treatment, the ratio between PSI and PSII chlorophyll fluorescence (emission maximum at 735 and 690 nm, respectively) was increased in *gnat1* extracts, mirroring the emission characteristics of WT plant extracts. As expected, no detectable PSI-LHCII state transition complex formation upon red light illumination was detected in *gnat2-1* (Fig. 5*A*). Furthermore, the method of Pulse Amplitude Modulated (PAM) fluorometry was applied to track LHCII antennae adjustment in a real-time manner depending on the predominance of red or far-red light conditions. The maximum fluorescence yields of PSII and PSII-associated LHCII chlorophyll molecules were determined under both conditions and the offset value was monitored as qT parameter for state transitions. Additionally, the balancing of energy flow (qS) upon state transitions from state 1 to state 2 can be evaluated (Table 2 and Supplemental Fig. S6). Again, *gnat1-1* and *gnat1-2* did not differ from WT plants, neither in qT nor in qS. The deficiency of state transition performance of *gnat2-1* mutants, however, becomes clearly apparent, since qT and qS were notably reduced as it was also previously observed by Koskela and coworkers (27).

Since an increased association of RBCL and RCA to the thylakoid membranes has been reported for *gnat1-2* and *gnat2-1* in previous studies, we monitored the rate of CO_2_ assimilation in the mutant lines compared to WT plants (81). In relation to increasing light intensities applied in the experimental setup, the mutant plants exhibited a slightly higher rate of CO_2_ fixation than WT, especially in the range of high light intensities (Supplemental Fig. S7). Starting from a PPFD of 400 μmol · m^-2^ · s^-1^ and higher, most notably *gnat1-2* showed a significantly enhanced CO_2_ assimilation with a rate about 10 to 15% higher than WT. When investigating the ultrastructural thylakoid membrane organization, a similar phenotype of *gnat1* and *gnat2* lines compared to WT could be observed (Supplemental Figs. S8 and S9). A more compact packing of grana stacks in *gnat2* was previously reported and could be again identified for *gnat2-1* in this study (27). By analyzing transmission electron microscopy images obtained from mutant and WT chloroplasts, more compact grana stacks were also detected in *gnat1-1* and *gnat1-2* (Supplemental Fig. S8*A*). However, *gnat1* mutant lines showed more grana stacks with a lower number of membranes compared to WT and *gnat2* (Supplemental Fig. S8, *B* and *C*). In both *gnat1* mutant lines, only 1.0 to 1.3% of all grana stacks consist of 10 membrane layers or more, whereas in chloroplasts of WT or *gnat2-1* mutants around five times as many can be found.

### High Light Treatment Strongly Affects the Photosynthetic Performance of GNAT2 but not of GNAT1 Seedlings

To reveal whether the significant downregulation of PSBD-K7 acetylation in the *gnat1* mutant plants could affect the integrity of PSII upon high light treatment, we exposed 8-day-old seedlings grown on sterile media plates of both *gnat1* lines as well as WT and *gnat2-1* plants to continuous high light (HL) conditions or continuous darkness (D). A notable drop in the maximum quantum yield F_v_/F_m_ was detected for *gnat2-1* treated with HL (Fig. 5, *B* and *C* and Supplemental Table S6). With a constant decrease starting from day 1 of HL, *gnat2-1* mutant seedlings exhibited a depletion of photosynthetic activity by nearly 50% on day 3 of HL in comparison to WT and *gnat1* mutants (Fig. 5*B*). The same trend was observed for the effective quantum yield Y(II) determined by analyzing the light response of the seedlings via Imaging-PAM measurements (Supplemental Table S6). The difference between *gnat2-1* and WT or *gnat1* plants was especially striking in the range of low-to medium-range light intensities applied in the course of the light response analyses. Interestingly, the decrease in the yield of photochemical quenching was compensated in *gnat2* on day 1 of HL mainly by an increase in the yield of non-regulated energy dissipation Y(NO). While Y(NO) decreased back to WT or *gnat1* level on days 2 and 3, the yield of non-photochemical quenching Y(NPQ) was then increased, indicating an upregulation of light-induced energy quenching mechanisms in *gnat2-1*. This increase in Y(NPQ), also reflected by the coefficients NPQ and qN, required an acclimation phase since it was only observable after 2 days of HL. Hence, we can conclude that the defect in lysine acetylation of PSBD in *gnat1* does not result in an altered high light sensitivity in these lines, but it appears conceivable that the more severe defect of KA and NTA of photosynthetic proteins in *gnat2-1* contributes to the high light sensitivity of this mutant.

### Pulldown and BiFC Analyses Reveal a Strong Interaction of GNAT1 with GNAT2

**Fig. 6:**
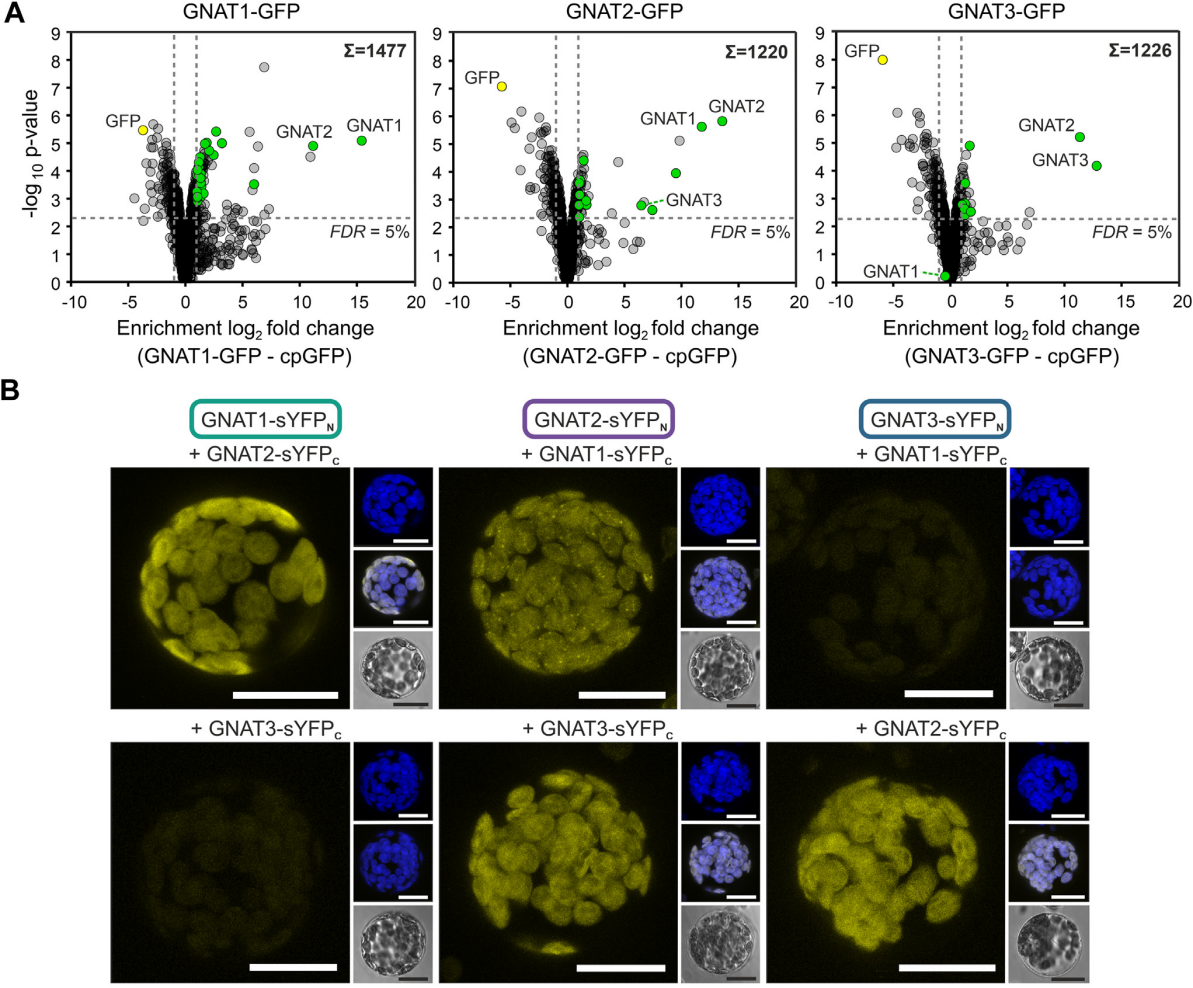
**GNAT1 exhibits a strong interaction with GNAT2 as revealed by co-immunoprecipitation and BiFC analyses.** Volcano plots of co-immunoprecipitation assays performed on leaf material of GNAT1-, GNAT2-or GNAT3-GFP overexpressing plant lines (*A*). Sums represent the total number of quantified protein groups per experiment by Label-Free-Quantification (LFQ) *via* MaxQuant. For each protein group, the log_2_ fold change was calculated by comparing LFQ intensities of the respective GNAT-GFP experiment with those of the control experiment comprising the co-immunoprecipitation of chloroplast-targeted GFP (cpGFP). Per genotype, four biological replicates were harvested and processed. To be recognized as significant, valid quantifications in at least three of four biological replicates per experiment were required, and in addition, enriched protein groups had to possess a log_2_ fold change of ≥1 and a false discovery rate-corrected *p*-value ≤0.05 (LIMMA). *Green circles* highlight protein groups with assigned chloroplast localization according to Suba4 (74). *Yellow circles* represent the GFP protein that was found in the control experiment as well as in samples of the GNAT-GFP overexpressors as part of the respective fusion proteins. *B*, confocal Laser Scanning Microscopy (CLSM) analysis of protein interactions between GNAT1, GNAT2, and GNAT3 by Bimolecular Fluorescence Complementation (BiFC). Arabidopsis wild type (WT) protoplasts were transiently transformed with GNAT plasmid constructs enabling the expression of recombinant GNATs C-terminally fused to a split-YFP domain. Per assays, combinations of two GNATs were tested for interaction, which can be detected by YFP fluorescence emission as a result of sYFPN- and sYFPC-domain interplay. The main panels represent protoplasts in the channel of YFP detection showing either a fluorescence signal or in case of missing interaction a weak background emission obtained under the same conditions of signal detection. The three small images on the *right side* of each main panel display the chlorophyll autofluorescence (*top*), a merged view of YFP and autofluorescence signals (*middle*), and a bright-field image (*bottom*) of the respective protoplast. The scale bar corresponds to a distance of 20 μm.

To identify proteins that could have a regulatory impact on GNAT1, we investigated its interactions with other proteins with the help of Arabidopsis lines overexpressing GNAT1 C-terminally linked to GFP. For a quantitative comparison, a plant line overexpressing chloroplast-targeted GFP (cp-GFP) served as a background control to identify non-specifically GFP-interacting proteins (68). A co-immunoprecipitation assay using GFP-trap beads was performed on plant leaf extracts from 6-7-weeks-old plants followed by the identification and quantification of co-isolated proteins by mass spectrometry (IP-MS) from four biological replicates each. In the GNAT1-GFP pulldown, a strong enrichment of GNAT2 (around 2000-fold) next to the pulled GNAT1-GFP was observed (Fig. 6*A*). To confirm the interaction between these two GNATs, the same experiment was also conducted using a GNAT2-GFP Arabidopsis overexpression line. Also, by introducing GNAT2 as the bait for interacting proteins, the strong GNAT1-GNAT2 interaction could be verified (Fig. 6*A*). Intriguingly, next to GNAT1 the third GNAT of the NAA90 subfamily, GNAT3, appeared as one of the most abundant prey proteins in the GNAT2 pulldown. In a third IP-MS experiment using GNAT3-GFP as bait for interacting proteins, the association with GNAT2 could be confirmed, whereas GNAT1 was not detected among the co-enriched proteins (Fig. 6*A*).

In the GNAT1-GFP pulldown, 23 additional interacting proteins were identified with an enrichment factor of at least 2-fold (Supplemental Datas S4 and S6). Among those proteins, Seedling Plastid Development 1 (SPD1), a member of the nucleoside-triphosphate hydrolase superfamily, was identified as 60-fold enriched. In addition to SPD1, the co-eluted proteins Accumulation and Replication of Chloroplasts 11 (ARC11), FTSZ1-1, FTSZ2-1, FTSZ2-2, CPN60A, RABE1b, and HCF136, indicate an enrichment of proteins involved in the process of plastid organization. Within this cluster, an enrichment of proteins involved in chloroplast fission (ARC11, FTSZ1-1, FTSZ2-1, FTSZ2-2) was noticed. Furthermore, the 6-Phosphogluconate Dehydrogenases PGD1 and PGD3 of the oxidative pentose phosphate pathway were co-precipitated. Enzymes from sulfate assimilation, such as the ATP sulfurylases APS1, APS2, and APS4, proteins involved in arginine and proline synthesis, and proteins involved in photosynthesis (CRR41, LHCB4.3, HCF136, FNRL, CPN60A), plastid transcription (RNA Polymerase subunit Alpha, PTAC17) and protein synthesis (RABE1b, RPL16, SVR3) were among the co-enriched proteins. Interestingly, of these putative GNAT1 partners, no candidate was identified to undergo significant acetylation by GNAT1, neither in NTA nor in KA (Supplemental Datas S1 and S3).

The immunoprecipitation of GNAT2-GFP resulted in the co-enrichment of six unique plastidial proteins apart from GNAT1 and 3 (Supplemental Datas S4 and S6). The most co-enriched protein was the Enhancer of NSI Activity (ENA; more than 700-fold), which is a DnaJ-type chaperone with a cysteine-rich (CR) domain. An enrichment of nearly 180-fold was observed for a poorly characterized Tetratricopeptide repeat (TPR)-like superfamily protein (AT1G78915), followed by proteins enriched by about two-fold, such as two proteins involved in protein synthesis (RPS17, RPL9), the plastidial histone deacetylase 14 (HDA14), and Cold-Regulated 15B (COR15B), which is involved in the protection against abiotic stresses. Only three proteins were found co-enriched by about two-fold in both GNAT1-and GNAT2-GFP pulldowns. Among those, the Translocon at the outer Envelope Membrane of Chloroplasts 75-III (MAR1), Cold-Responsive 6.6 (KIN2), and a Cyclophilin-like peptidyl-prolyl cis-trans isomerase family protein (CYP26–2) were identified (Supplemental Datas S4 and S6). Our results indicate that GNAT1 and GNAT2 can form a stable complex, but both enzymes also interact with other proteins independently.

None of the plastidial proteins identified as enriched upon GNAT immunoprecipitation exhibited significant up- or downregulation in its total abundance, pointing towards a true interaction with the GNATs (Supplemental Fig. S10, Supplemental Datas S5, and S6). Interestingly, within the set of de-regulated protein groups in GNAT3-GFP overexpression lines, the level of quantified GNAT1 was reduced to less than 20% in comparison to the plastid GFP control line. In contrast, neither GNAT1-nor GNAT2-GFP overexpression revealed an impact on the other physiologically expressed GNATs (Supplemental Datas S5, and S6).

To confirm the interactions between the GNATs, we performed a bimolecular fluorescence complementation (BiFC) assay using Arabidopsis protoplasts. For this purpose, the GNATs were C-terminally fused to either an N- or a C-terminal domain of a split-YFP protein. Co-transformation of protoplasts with GNAT combinations resulted in the identification of interacting GNATs in a pairwise manner indicated by the fluorescence emission of YFP. YFP-dependent fluorescence signals that were detected by confocal laser scanning microscopy (CLSM) were reported for combinations of GNAT1-GNAT2 and GNAT2-GNAT3, whereby both GNAT variants, either coupled to the N- or to the C-terminal domain of YFP, enabled the complementation of the YFP protein (Fig. 6*B*). No direct interaction between GNAT1 and GNAT3 was observed, which supports the results of the GNAT-GFP pulldowns.

### Cross-Linking Experiments Reveal Potential Heterodimer Formation and Independent Complexes Between GNAT1-GNAT2 and GNAT2-GNAT3

**Fig. 7:**
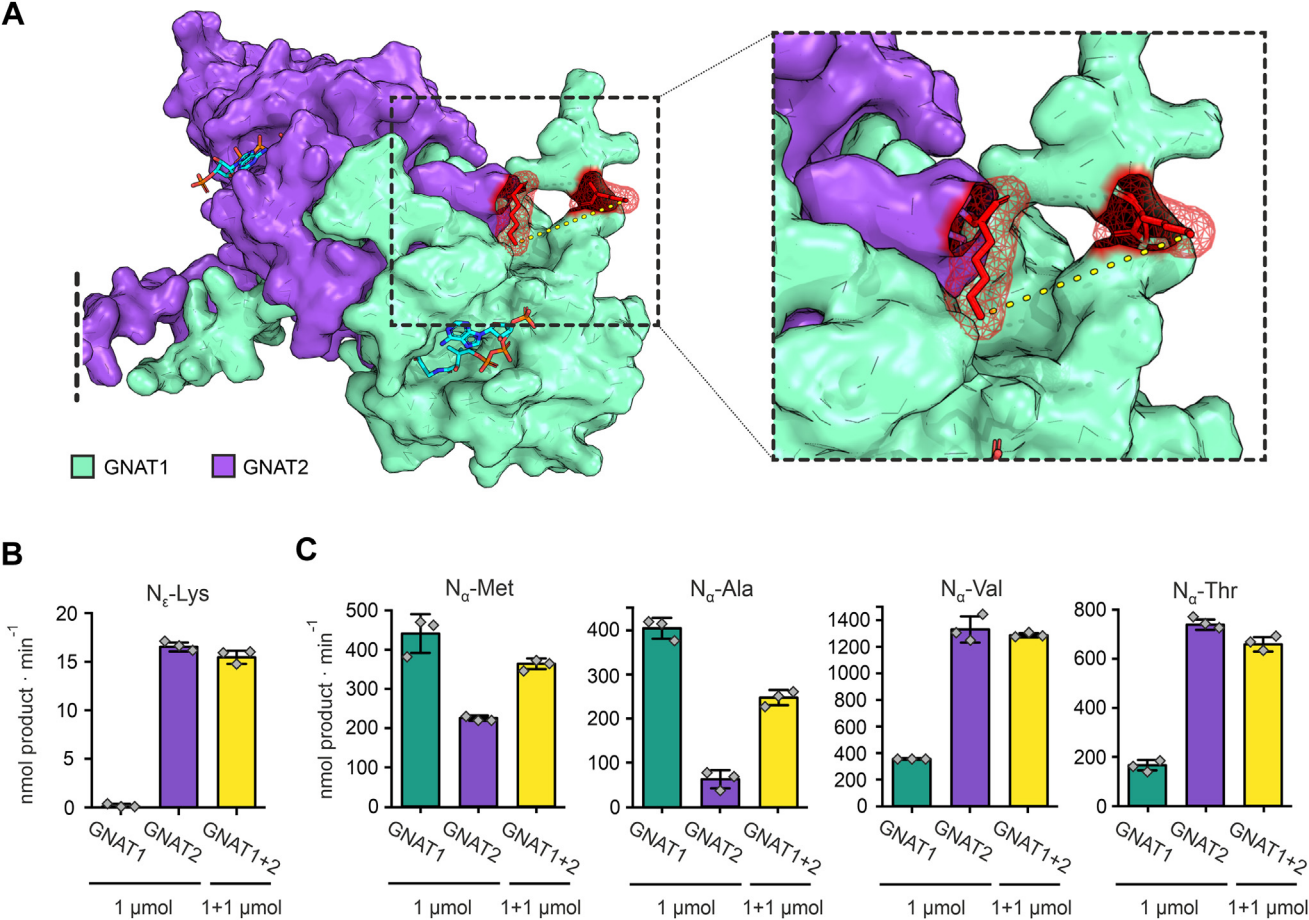
**Chemical cross-linking of GNAT1 and 2 during co-immunoprecipitation and combined *in vitro* activity assays.***A*, structure predictions for the heterodimer GNAT1-GNAT2 derived by AlphaFold 2 Multimer and visualized using PyMOL (Evans *et al.*, 2021). Sequence data were obtained from the Araport 11 database and N-terminally truncated by the amino acid sequence corresponding to predicted chloroplast transit peptides. The lysine residues involved in the formation of the identified DSSO protein cross-link were shown as *red stick* models covered by the *red*, partially transparent mesh structure of the computed protein surface. The *dotted yellow line* marks the distance between the two amino groups of the lysine pair revealing a value of 14.3 Å. The position of the CoA molecule presented as a stick model was derived from a structure prediction provided by the AlphaFill database (Hekkelman *et al.*, 2021). N_ε_-Lysine (*B*) and N_α_-N-terminus acetylation (*C*) activity defined for single GNATs and a combination of His_6_-GNAT1 and His_6_-GNAT2. Both proteins were used in a concentration of 1 μM (single) or 1 + 1 μM (GNAT1 + GNAT2) and mixed with a synthetic peptide substrate in a concentration of 100 μM. Subsequently, the reaction was started by adding 200 μM of the acetyl-group donor acetyl-CoA, and an aliquot corresponding to 1000 pmol was analyzed by HPLC to detect a retention-time shift indicating the addition of an acetyl-group. The bar graph represents the fractions of acetylated peptide (product) generated per minute within the linear phase of the acetylation reaction (n = 3, ±SD).

To identify the interaction sites between the GNATs, the protein cross-linking reagent disuccinimidyl sulfoxide (DSSO) was used. With the help of this chemical cross-linker, lysine residues at a distance of around 10 Å can be coupled, and the linked fragments of proteins identified by mass spectrometry. Only two inter-molecular cross-linked GNAT fragments were detected in the GNAT-GFP pulldowns: one coupling GNAT1 and GNAT2, and the other one connecting GNAT2 and GNAT3 (Fig. 7*A*, Supplemental Figs. S11, and S12). Modeling results obtained by AlphaFold 2 Multimer structure prediction of the corresponding hetero-dimers further support a dimerization of these GNATs (43). All lysine residues of both cross-links are located within the interface area of the corresponding dimer models with a predicted distance of 14.3 (GNAT1-GNAT2, Fig. 7*A*) and 5.1 Å (GNAT2-GNAT3; Supplemental Fig. S12). Noteworthy, confidence score calculations by AlphaFold 2 strongly suggest dimerization as the most likely form of GNAT multimerization with hetero-as well as homo-dimer predictions reaching high ranks of confidence (Table 3 and Supplemental Fig. S13). Modeled oligomers consisting of more than two GNATs, however, fall substantially in their score ranking (Supplemental Table S4).

**Table 3:** Confidence score values calculated by AlphaFold 2 Multimer for all homo- and hetero-dimer predictions including GNAT1, 2, and three

Modeled GNAT dimer	AF2 confidence (iptm + ptm)
GNAT1-GNAT1	0.882
GNAT2-GNAT2	0.817
GNAT3-GNAT3	0.802
GNAT1-GNAT2	0.882
GNAT1-GNAT3	0.881
GNAT2-GNAT3	0.815

The average quality of each complex is evaluated by the intrinsic model accuracy estimates pTM and ipTM (predicted Template Modeling Score and interface predicted Template Modeling Score) and represented as a value between 0 to 1.

### *In Vitro* Assays Indicate that the Interaction of GNAT1 and GNAT2 Does Not Boost Their Acetyltransferase Activities

After discovering the mutual affinity between GNAT1 and GNAT2 by co-immunoprecipitation from Arabidopsis leaves, recombinant His_6_-GNAT1 and His_6_-GNAT2 were combined in the *in vitro* activity assay to analyze the effects of their interactions on their acetyltransferase activities. The N_ε_-Lys as well as four different N_α_-N-terminus substrate peptides, which have been demonstrated to correlate with the substrate preferences of GNAT1 and/or GNAT2 (Fig. 1*C*), were tested either with a single GNAT or a combination of both. Interestingly, the mixing of GNAT1 (1 μM) and GNAT2 (1 μM) did not result in a burst, not even an addition of the individually determined acetyltransferase activities (Fig. 7, *B* and *C*). The highest difference can be observed throughout the acetylation of the N_α_-Ala peptide. While in the case of additive total GNAT activity a substrate conversion rate of around 450 nmol ∙ min^-1^ would be expected, the effective catalytic activity turned out to be only about 250 nmol ∙ min^-1^, far less than the individual activity of GNAT1. In a similar range, with an effective activity of only about 55% of the hypothetical activity, the N_α_-Met peptide substrate was acetylated by GNAT1 and GNAT2. For the substrates N_α_-Val and N_α_-Thr, a smaller difference between effective and hypothetical conversion rates was observed with a percentage of around 76% and 73% of effective activity, respectively. The smallest difference can be determined in the case of the lysine acetylation rate of the corresponding N_ε_-Lys peptide, which enables an effective acetylation activity of 93% for the combination of GNAT1 and GNAT2. However, since GNAT1 shows negligible rates in acetylation of the N_ε_-Lys substrate in the single GNAT assay, GNAT2 is probably mainly responsible for the resulting enzymatic activity (Fig. 7*B*). The lower catalytic rates in the combined assay might be explained by the competition of both enzymes for their substrates.

## Discussion

Protein acetylation is a prevalent modification in chloroplasts of plants (13, 15, 85, 86). Eight novel enzymes that possess dual lysine and N-terminus acetylation activities have been recently discovered in Arabidopsis, which are conserved in the plant kingdom (25, 26). GNAT1, GNAT2, and GNAT3 show high phylogenetic proximity, as they cluster on one distinct branch of the derived phylogenetic tree of Arabidopsis acetyltransferases, thereby defining a specific subtype of the GNAT-related plastidial acetyltransferases (26). This subgroup was later named NAA90, with at least one ortholog being present in all representative members of the plant kingdom (25). While most of the GNAT mutants have no apparent growth phenotype under standard growth conditions, *gnat2* was shown to be essential for photosynthetic state transitions and has a delayed growth phenotype, especially under fluctuating light conditions (27, 31, 81). Here, we have expanded our knowledge concerning the role of the closely related GNAT1 enzyme.

### Structural Characteristics of the Arabidopsis NAA90 Enzymes in Comparison to Other GNAT Acetyltransferases

**Fig. 8:**
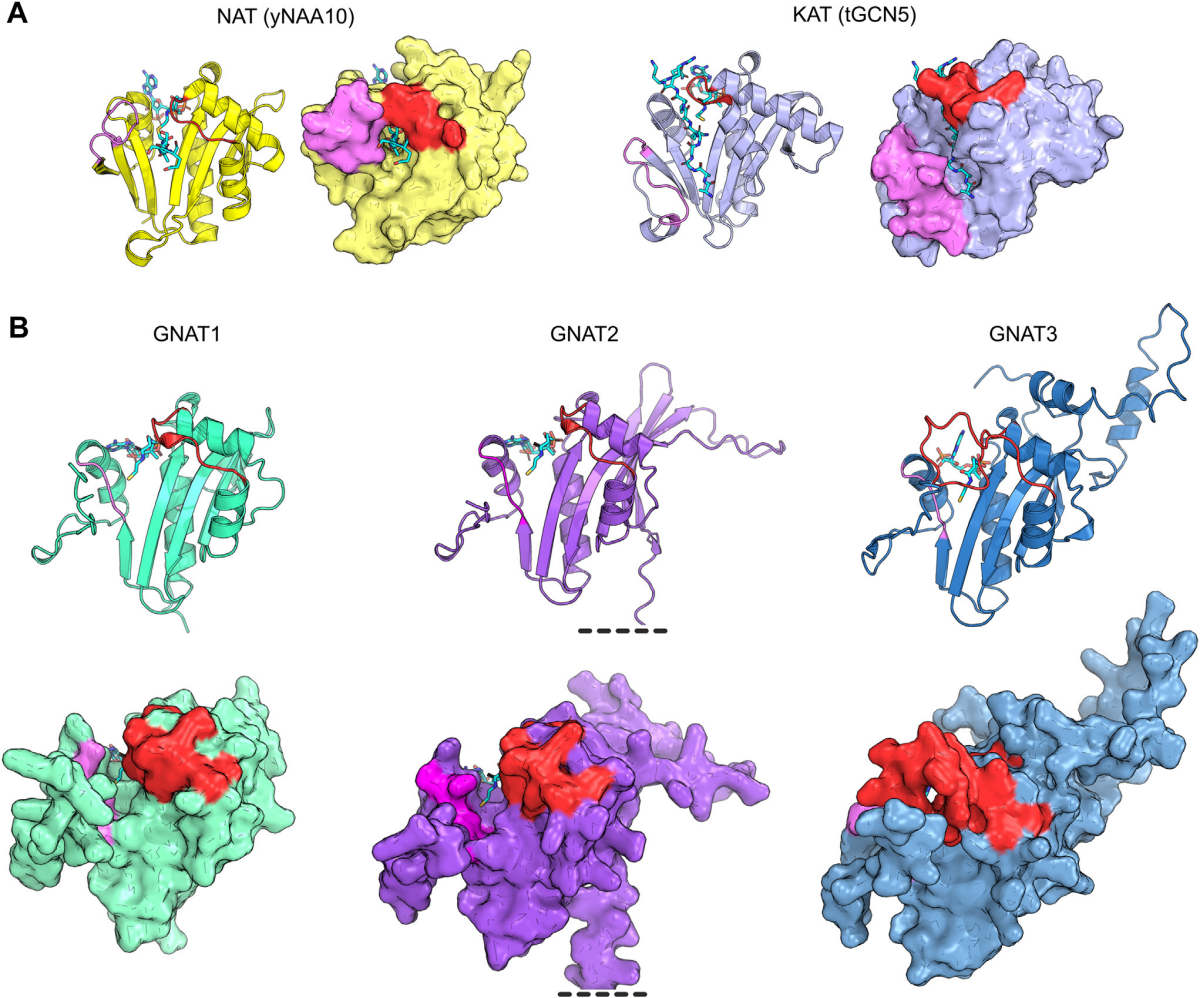
**AlpaFold 2 structure predictions indicate the presence of accessory features flanking the more conserved ‘core’ domain.***A*, crystal structures of NAA10 from *Schizosaccharomyces pombe* (PDB code 4KVM) and GCN5 from *Tetrahymena thermophila* (PDB code 1QSN) as well as structure models of GNAT1, 2, and 3 (*B*) provided by AlphaFold 2. The region of the α1-α2 loop is colored in *red* and the β6-β7 loop or the corresponding β5-α4-loop in GNAT1, 2, and 3, respectively, is colored in *magenta*. Each GNAT model was trimmed by the N-terminal region representing the plastid transit peptide. Furthermore, the extensive N-terminal loop of GNAT2 was removed from the corresponding images as indicated by the *dashed black lines*.

A structurally conserved fold of β-sheets and α-helices, which is shared by the majority of the GNAT acetyltransferases, was previously identified (1, 4). One of its central elements is formed by the acetyl-CoA binding site located in the turn between strand β4 and helix α3. In its architecture, it is arranged by additionally recruiting helix α4 and strand β4 (Fig. 8*A*; (29). Moreover, the strands β1-β4 and β5-β6 exhibit an almost even surface of antiparallel organization resulting in a characteristic V-shaped configuration. Accessory structural features can be typically found in flanking N- and C-terminal regions contributing to the different functions of distinct GNATs (4). It is assumed that individual substrate preferences are mainly defined by the configuration of the β6-β7 and the α1-α2 loop, which appear to form a narrow groove or even a tunnel in case of evident NATs, thereby impeding an interaction with lysine residues of KAT-specific substrates (Fig. 8*A*; (28, 29)). The substrate binding site of a typical KAT, such as the histone acetyltransferase GCN5, exhibits a rather open architecture and a less pronounced hairpin-like loop between the sheets β6 and β7 (Fig. 8*A*; (28, 87)). In the case of the new plastidial GNAT family in Arabidopsis, no experimentally evaluated structure information is available. An acetyltransferase with a high degree of identity to GNAT2 of *Arabidopsis* is the serotonin *N*-acetyltransferase SNAT1 of *Oryza sativa*, which was mainly characterized by its function in melatonin biosynthesis (88). As its crystal structure was recently resolved (89), SNAT1 might allow a first substantiation of structural interpretations drawn for its closest homologs GNAT2, GNAT1, and GNAT3 in Arabidopsis. To reveal the characteristic features of GNAT1, novel machine learning modeling approaches such as AlphaFold 2 were used as a valuable tool helping to classify this GNAT, which provides a new layer of conclusive information that facilitates the investigation of still poorly understood functional domains of proteins (90). A remarkable congruence between the experimentally derived SNAT1 structure and a corresponding AlphaFold 2 model can be noted (Supplemental Fig. S15*A*). In a comparable range, the modeled GNAT2 and the experimentally derived SNAT1 structure resemble one another, especially in the core region flanking the acetyl-CoA binding site (Supplemental Fig. S15*C*). An interesting feature of GNAT2 that differs from SNAT1 is the extended loop between the β-sheets 2 and 3, which shows an accumulation of serine residues directly downstream to β-sheet 2 followed by several residues of negative charge, such as aspartate and glutamate (Supplemental Figs. S15*C* and S16). Interestingly, the predicted models of GNAT1, 2, and 3 as well as the crystal structure of SNAT1 indicate the absence of the β-strands 6 and 7, so that the region defining the substrate binding surface area appears smaller and more accessible than the substrate binding sites of “classical” KATs and NATs (Fig. 8*B* and Supplemental Fig. S14). Since the region between sheet β5 and helix α4 is arranged in a position directly facing the α1-α2 loop instead, it might contribute to the shaping of the substrate binding site as well. In GNAT1 and 2, both, the α1-α2 and the β5-α4 loop exhibit a very high confidence (pLDDT >90) in the context of architectural organization, possibly indicating a more or less rigid conformation. In GNAT3 in contrast, the α1-α2 loop appears to be much more enlarged and covers the substrate binding surface completely (Fig. 8*B*), though the confidence index is low to very low (pLDDT <70 or even <50) and the arrangement therefore rather dynamic (Supplemental Figs. S14 and S17). In addition to the structural resolution of monomeric SNAT1, the study of Liao and coworkers also provides models presenting the homodimerization of SNAT1 as its preferred configuration, thereby revealing again a high degree of congruence between the experimentally derived SNAT1 homodimer and a GNAT2 homodimer model generated by AlphaFold 2 Multimer (Supplemental Fig. S15*E*; (43, 89)). Experimentally derived structure information is necessary for a robust interpretation of functional elements and a deeper understanding of structural dynamics, for instance in the process of substrate interaction or complex formation.

### NAA90 Enzymes Form Complexes *in vivo*

The first experimental evidence of GNAT oligomerization within the chloroplast was revealed in this study by four different approaches (coIP, cross-link MS, BiFC, and peptide assays) *in planta*, *in vivo*, and *in vitro* setups. Throughout the profiling of proteins interacting with GNAT1, we found GNAT2 to be highly co-enriched during the GNAT1-GFP pulldown experiment and could confirm this interaction in a reversed set-up (Fig. 6*A*). Furthermore, GNAT3 turned out to form a stable complex with GNAT2 as shown by GNAT2-GFP as well as GNAT3-GFP immunoprecipitation. These interactions between GNAT1 and GNAT2 and GNAT2 and GNAT3 were also traceable in transient split-YFP assays in Arabidopsis protoplasts in combination with CLS microscopy (Fig. 6*B*). Including a chemical protein cross-linker in the procedure of GNAT-GFP immune-precipitation further underpinned the detected interactions and uncovered the concrete lysine residues of the respective GNATs that are in close proximity (Fig. 7*A* and Supplemental Fig. S12). When mapping the lysine residues involved in the GNAT1-GNAT2 and the GNAT2-GNAT3 interaction in AlphaFold 2 Multimer-predicted dimer structures, both, the experimentally as well as computationally derived information can be consistently brought together. Notably, both cross-links comprise lysine 258 (K258) of GNAT2, the terminal amino acid residue of its C-terminus, which appears to stretch out onto the cleft-like structure of GNAT1 or GNAT3 that forms the substrate binding site in both proteins, respectively. The dimer models of GNAT1-GNAT2 and GNAT2-GNAT3 indicate that the same surface-exposed amino acid residues of GNAT2 are involved in both interactions making the formation of multimers containing more than two GNATs rather unlikely to occur (Fig. 7*A*, Supplemental Figs. S12, and S18). This conclusion is also supported by the AlphaFold 2 multimer-confidence score index (Table 3 and Supplemental Table S4), according to which all multimeric structures of three or more GNATs drop substantially in their confidence ranking (43). Unraveling the functional impact of the GNAT dimer formation will be a promising subject of future research.

### *In Planta* Substrate Preference of GNAT1 Indicates its Major Function as N-terminal Acetyltransferase

In the case of the cytosolic NAT machinery, the composition of distinct complexes in conjunction with the impact of its individual subunits is already well understood for many eukaryotic organisms (15, 91, 92). Our analyses on the interplay between recombinant GNAT1 and GNAT2 revealed that a combination of both acetyltransferases did not result in a burst of activity, not even in a summation of the individual catalytic activities that were determined in the same setup for both enzymes separately (Fig. 7*B*). The highest difference between the effective substrate conversion rate measured for a GNAT1-GNAT2 combination and a hypothetical enzymatic activity was thereby observed for the N_α_-Ala peptide. In the presence of both GNATs, only 53% of turnover was achieved, if 100% stands for the hypothetical sum of both individual activities. N_α_-peptide substrates that were predominantly favored by GNAT2 showed a smaller difference between effective and hypothetical GNAT activity. In general, the *in vitro* preference of recombinant GNAT2 for Val and Thr as N-terminal amino acids for acetylation is consistent with the previously reported activity data (26). The remarkably higher level of enzymatic activity observed in our study might be due to the different structural properties of the recombinant GNAT2 used here and the one introduced by Bienvenut and coworkers since the previously characterized variant was N-terminally coupled to a maltose-binding protein (MBP). In the comprehensive characterization performed in this study, all GNATs have been also analyzed for their NTA-substrate preferences in a cellular context during heterologous overexpression in *E. coli* (26). On bacterial proteins, recombinant GNAT2 revealed only a relatively small subset of NTA-targets starting with a valine residue, whereas *in vitro*, the purified GNAT2 showed a clear preference for the N_α_-Val peptide. However, the major subgroup of *E. coli*-proteins targeted by GNAT2 comprised a methionine residue at their N-terminus. GNAT1 exhibited only a very low yield of NTA during heterologous overexpression on *E. coli* proteins so no clear substrate preference could be derived. Though, the limited set of substrate N-termini already indicated a specificity for GNAT1 in accordance with NatA-type acetyltransferases favoring Ala, Val, Ser, and Thr (26). This is also reflected by our NTA profile derived from *gnat1* mutant plants in comparison to WT. In general, the divergence in the substrate spectra, which can be observed in *in vitro* test systems, *in vivo* in *E. coli*, or *in planta*, might be explained by the very different testing conditions affecting the enzymatic activity. Specific factors might modulate activities and substrate preferences of GNAT1 and GNAT2 within their physiological cellular environment resulting in altered specificities when these factors are missing.

### The Interaction of GNAT1 and GNAT2 is not Required for State Transitions or Acclimation to High Light

Very recently, a subset of the plastidial GNATs (GNAT1, GNAT2, GNAT4, GNAT6, GNAT7, and GNAT10) was analyzed in a reverse genetics approach with a focus on changes in metabolite profiles and photosynthesis (81). There, it was reported that these GNATs have individual impacts on a variety of metabolic pathways in Arabidopsis. In comparison to the other *gnat* mutants, *gnat1* only showed a few specific features on metabolome or phenotype level, such as the slight accumulation of free serine. Ivanauskaite and co-workers identified *gnat2* as the only GNAT-deficient plant line with a visible phenotype exhibiting lower biomass and slightly differing photosynthetic properties in comparison to WT (81). Interestingly, the retarded growth phenotype of *gnat2* was only apparent under low light cultivation and the effects on fresh weight as well as on rosette size diminished when higher light intensities were applied. A light-dependent phenotype of *gnat2* was also described in previous studies, for instance within a comparative analysis of growth and photosynthetic performance under fluctuating light cultivation or manifested as delayed flowering and retarded developmental stage phenotype; in the latter case again especially apparent under low light conditions (31, 93). No phenotypic difference between WT and two independent *gnat1* mutant plant lines could be revealed under the tested light conditions, as well as no impact on the plants in their performance of photosynthetic state transitions. To test, if the adaption to high light could be affected in *gnat2* or *gnat1* mutant plants, we exposed seedlings of both genotypes to continuous high light illumination and evaluated their photosynthetic performance. A substantial drop in the maximum and effective quantum efficiency was observed in *gnat2* after 1 day of high light, along with a notable increase in the part of non-regulated energy dissipation Y(NO) (Fig. 5, *B* and *C* and Supplemental Table S6). This finding points towards a regulatory impact of GNAT2 in the process of light acclimation since there was no sign of recovery for the maximum and the effective quantum yields detectable in the following days of high light treatment. The part of non-photochemical quenching of excessive energy Y(NPQ) showed a notable increase from the second day after the start of high light treatment, coincidently with a beginning decrease of Y(NO) (Supplemental Table S6). This phenotype of a delayed NPQ upregulation, plausibly in response to the high level of potentially damaging, non-regulated energy dissipation, supports an additional function of GNAT2 in light acclimation beyond its role in the regulation of state transitions. For adaption to high light conditions, the precise adjustment of both types of photosynthetic electron transport, the linear and the cyclic electron flow, is well-known to be a significant factor. The cyclic electron flow (CEF) is characterized by a cycling of electrons around PSI, thereby comprising the cytochrome *b*_6_*f* complex, PSI, the small electron carrier Ferredoxin, and specific proteins, which are able to re-inject the shuttled electrons into the plastoquinone pool, such as the proteins PGRL1 and PGR5 or the NADH dehydrogenase-like complex NDH-1 (94, 95, 96). Due to this, the CEF has an essential function in preserving the photosynthetic electron flow, when no final acceptors downstream from the primary light reactions, such as NADP^+^, are available. Moreover, it is assumed that by increasing the proton influx into the lumen the CEF may play an important role in the high light-dependent downregulation of PSII and PSI activity by non-photochemical quenching and photosynthetic control, respectively (97, 98, 99). The identification of proteins related to CEF among the NTA substrates of GNAT2 might hint at a defect in CEF in *gnat2*. The identified proteins comprise, for instance, the NDH-1 subunit PSNB2, the Ferredoxin-NADP^+^ reductase FNR2, as well as the plastidial translocon subunit TIC55 that is described to form a redox-regulon involved in membrane-anchoring of the FNR (Supplemental Data S3; (100, 101, 102)). Intriguingly, some of the other proteins targeted by GNAT2-mediated NTA reveal a link to the repair and/or *de novo* synthesis of PSII, namely, the PSII assembly factor HCF244 or the protein LPA3, a thylakoid membrane protein, which also appears to be involved in the maturation of PSII (103, 104). In addition, the NTA site with the strongest downregulation in the NTA yield identified in *gnat2* mutants (from a yield of 99.1% in the WT to 1.8% in *gnat2*, NTA yield difference of −97.2%), can be assigned to the plastidial S-sulfocysteine synthase CYSK4. Plants deficient in CYSK4 were described to reveal an accumulation of ROS, a remarkably reduced maximum quantum yield, and an upregulation of NPQ (105, 106). Altogether, it can be summarized that the NTA profile of *gnat2* points towards the involvement of the CEF and/or PSII assembly machinery in the mediation of the *gnat2*-specific phenotype. However, the precise mechanisms through which GNAT2 operates need to be deciphered in future work. No apparent phenotype could be detected for seedlings of the two GNAT1-deficient plant lines upon high light treatment, even though the KA profile pointed towards a potential function of GNAT1 in photosynthetic adaptation by revealing a remarkable down-regulation of the acetylation state of the PSII core protein PSBD (D2). For the acetylation site K7 of the PSBD protein, a remaining KA abundance of less than 1% in *gnat1* compared to the WT was detected. Together with the PSII subunit PSBA (D1), PSBD builds the reaction center of PSII in the form of a heterodimer and coordinates its central chlorophyll *a* dimer P_680_. Additional inner antennae proteins and several membrane-intrinsic low-molecular-mass subunits surround the reaction center, thereby forming the full PSII complex. Interestingly, PSBD is involved in the first steps of PSII *de novo* assembly by binding the cytochrome b_559_ and subsequently initiating the PSII assembly process (82, 107). Recently, it has been reported that a substantial increase in PSBD transcription, which was obtained by inserting a strong promotor construct upstream to the *psbd* gene, did not result in enhanced protein abundance in tobacco plants (108). In contrast, a reduction of the PSBD transcript level led to a considerable decrease, not only in PSBD abundance but also in PSII accumulation. These findings highlight the limiting function of PSBD synthesis on PSII assembly, but also indicate a strict control of PSBD protein abundance, presumably by regulatory mechanisms that target protein stability and degradation (108). An important factor that can modulate the lifetime of a protein is the post-translational protein modification, such as the acetylation of lysine residues. Whether the acetylation of K7 mediated by GNAT1 has a regulatory impact on the stability of the PSBD subunit, is a question to be answered in future research. Under the light conditions, which were applied in this study for the proteome analyses, no change in PSBD protein abundance could be observed in *gnat1* mutants. Intriguingly, a remarkable upregulation in the acetylation of K7 and a neighboring lysine residue, K10, was discovered previously in thylakoid preparations of a mutant line deficient in the plastidial histone deacetylase 14 (HDA14), underpinning the putatively regulatory importance of these sites (13). Also here, the altered levels in KA of the D2 protein were not accompanied by changes in protein abundance. The decrease in NTA abundance of several protein N-termini, for instance of the neo-N-terminus V61 of the zeaxanthin epoxidase (ZEP), led to the suggestion that GNAT1 could be involved in photosynthetic acclimation. ZEP catalyzes the conversion of zeaxanthin to violaxanthin via a two-step epoxidation reaction, whereby the formation of zeaxanthin from violaxanthin, in turn, is induced under high-light conditions as part of the energy-dependent, rapidly reversible component qE of the NPQ (109, 110). Moreover, when zeaxanthin accumulates, the zeaxanthin-dependent quenching component qZ is activated, which forms a proportion of the enduring, slowly reversible part of the NPQ (111). However, when comparing the NTA yield differences determined for *gnat1* plants with the NTA profile defined for GNAT2, it becomes apparent that the target sites attributed to GNAT1 form mainly a subset of the GNAT2 NTA substrate spectrum (Supplemental Fig. S5, Supplemental Tables S2, S3, and Supplemental Data S3). In line with this, seedlings deficient in GNAT1 did not reveal an NPQ phenotype upon HL treatment indicating a compensatory activity of GNAT2 in these plants (Supplemental Table S6).

In summary, our study provides important first insights into the specific features of GNAT1 and the interactions of GNAT1, 2, and 3 in the NAA90 subfamily. In future studies, it will be a promising task to examine the exact role of GNAT1 in complex with GNAT2. Further stress conditions that affect PSII assembly or electron flow balancing between linear and cyclic electron transfer should be tested on *gnat1* and *gnat2* mutant plants since our KA and NTA profiles indicate a potential involvement of GNATs in the regulation of these processes. On the other hand, higher-order mutant lines deficient in GNAT1 and GNAT2, or even GNAT1, 2, and 3, will be crucial to decipher the function of these complexes for photosynthesis regulation. Acetylome data derived from knockout plants as well as from heterologous overexpression of GNATs point towards a putative mutual compensation in the acetylation of specific sites. Furthermore, by additional *in vitro* activity assays the interplay between GNAT1, 2, and 3 could be further examined to decipher the preferred modes of multimerization.

## Data Availability

Lysine acetylome, pull-downs and full proteome data can be found at JPOST (identifier JPST002262): https://repository.jpostdb.org/entry/JPST002262.

In addition to that, spectra are also accessible via MSviewer. The data of the coimmunoprecipitation profiles can be accessed using the following URL: https://msviewer.ucsf.edu/prospector/cgi-bin/mssearch.cgi?report_title=MS-Viewer&search_key=sc2f0nxgso&search_name=msviewer and the data of the acK profiling here: https://msviewer.ucsf.edu/prospector/cgi-bin/mssearch.cgi?report_title=MS-Viewer&search_key=sjczk1ixdg&search_name=msviewer

N-terminomics data have been deposited to the ProteomeXchange Consortium (http://proteomecentral.proteomexchange.org) repository *via* the PRIDE submission tool: Project accession: PXD046152proteomecen. For every sample, search results (.xml) and raw files (.raw) have been made available, as well as Mascot Distiller project files (.rov) to allow the viewing of annotated spectra.

## Supplemental data

This article contains supplemental data (26, 27, 41, 42, 43, 44, 45, 60, 70, 72, 80, 84, 89).

## Conflict of interest

The authors declare that they have no conflicts of interest with the contents of this article.

## Supplementary Data

Suppl. Data 1+2

Suppl. Data 3

Suppl. Data 4+5

Suppl. Data 6

Suppl. Data 7

Suppl. Data 8

Suppl. Table 1+2+3

Suppl. Table 4

Suppl. Table 5

Suppl. Table 6

Suppl. Fig. 1

Suppl. Fig. 2

Suppl. Fig. 3

Suppl. Fig. 4

Suppl. Fig. 5

Suppl. Fig. 6

Suppl. Fig. 7

Suppl. Fig. 8

Suppl. Fig. 9

Suppl. Fig. 10

Suppl. Fig. 11

Suppl. Fig. 12

Suppl. Fig. 13

Suppl. Fig. 14

Suppl. Fig. 15

Suppl. Fig. 16

Suppl. Fig. 17

Suppl. Fig. 18
