# Supplementary material for: The Plastidial Protein Acetyltransferase GNAT1 Forms a Complex With GNAT2, yet Their Interaction Is Dispensable for State Transitions
Source: Mol Cell Proteomics. 2024 Sep 28;23(11):100850. doi: 10.1016/j.mcpro.2024.100850 (PMC11585782; doi:10.1016/j.mcpro.2024.100850)
Supplement: Suppl. Table 1+2+3 [file mmc7.pdf]

Supplemental Table 1. Comparison of plastidial target sites that are significantly downregulated in KA as a result of GNAT1 or GNAT2 inactivation. Fold-changes were derived by calculating the ratio of quantified lysine acetylation identified for knockout mutant lines versus wild type (WT) plants, whereby candidate sites with a fold-change ≥ 0.5 or ≤-0.5 and a significance of p ≤ 0.05 (false discovery corrected, LIMMA) were recognized as significant. Acetylation sites as well as protein groups that were experimentally identified, but not significantly regulated were marked with a circle in the respective column. Minus symbols stand for no identification in the corresponding experiment. Candidates, whose ID shows a superscript "x", originate from the *gnat2* lysine acetylation profile published previously (27). The subcellular localization was assigned by using SUBA4, unless otherwise stated, or in cases of unavailable or unclear classification by referring to the TAIR10 database. Classification of biological processes was assigned by using MapMap, unless otherwise stated, or in cases of unavailable or unclear assignment by consulting the Gene Ontology resource (GO) or the UniProt database. Candidates without official or submitted names were marked with an asterisk and referred to by using their UniProt accession ID. The global analysis of the proteomic profile and KA fold-changes is presented in the Suppl. Data 1 and 2 and corresponding plots are shown in Fig. 3 B and C.

					gnat1		***gnat2***	
Protein ID	Accession number (ARAPORT-11)	Subcellular localization	Biological process	Position of KA site	KA site log_2 FC	ProtG FC	KA site log_2 FC	ProtG FC
PSBD	ATCG00270	Plastid	Photosynthesis light reaction	K7	-6.80	o	-	o
KEA1/2[x]	AT1G01790/AT4G00630	Plastid	Transport, potassium	K168/K170	0.75 (KEA1/2)	o (KEA1) o (KEA2)	-5.37 (KEA1/2)	o (KEA1) o (KEA2)
PSBP-1[x]	AT1G06680	Plastid	Photosynthesis light reaction	K88	-	o	-3.70	o
Q9SJ31[x]*	AT2G05310	Plastid, Mitochondrion (GO)	-	K62	o	o	-2.29	o
FER1[x]	AT5G01600	Plastid	Metal handling	K134	o	o	-0.62	o
LHCB1.4[x]	AT2G34430	Plastid	Photosynthesis light reaction	K40	-0.23	-	-0.30	o
PSAH1/2[x]	AT3G16140/AT1G52230	Plastid	Photosynthesis light reaction	K138	- (PSAH1) - (PSAH2)	- (PSAH1) o (PSAH2)	-0.28 (PSAH1/2)	- (PSAH1) o (PSAH2)

Supplemental Table 2. Plastidial target sites that are significantly downregulated in NTA as a result of GNAT1 inactivation. The NTA yield differences between *gnat1* and wild type (WT) plants were analyzed with GraphPad 10. An unpaired t-test analysis with FDR 1% was applied, whereby the WT profile data obtained in this study and those published by Bienvenut and coworkers as well as by Eirich and coworkers were combined (26, 84). Sites revealing a yield difference of >20% were recognized as significant. The global analysis is presented in the Suppl. Data 3 and plots of NTA yield differences are shown in Fig. 3 D and Suppl. Fig. 5. The position of NTA within the corresponding target protein sequence is given as a combination of number and amino acid residue in a one-letter code. Subcellular localizations were assigned by using SUBA4, unless otherwise stated, or in cases of unavailable or unclear classification by referring to the TAIR10 database. Classification of biological processes was assigned by using MapMap, unless otherwise stated, or in cases of unavailable or unclear assignment by consulting the Gene Ontology resource (GO) or the UniProt database. Candidates without official or submitted names were marked with an asterisk and referred to by using their UniProt accession ID.

					gnat1		
Protein ID	Accession number (ARAPORT-11)	Subcellular localization	Biological process	Position of NTA	NTA yield in *gnat1* (mean, %)	NTA yield in WT (mean, %)	Difference *gnat1* - WT (%)
THIC	AT2G29630	Plastid	Co-factor & vitamin metabolism	T55	35.78	88.14	-52.36
SYNO	AT4G17300	Plastid	Protein activation	T60	20.55	69.05	-48.50
F16P1	AT3G54050	Plastid	Photosynthesis Calvin cycle	V61	42.20	88.18	-45.98
ZHD10	AT2G21530	Plastid (GO)	mRNA binding (GO)	A72	1.60	38.94	-37.34
F16P1	AT3G54050	Plastid	Photosynthesis Calvin cycle	A60	10.95	44.21	-33.26
PORC	AT1G03630	Plastid	Tetrapyrrole synthesis	T69	18.10	48.66	-30.56
NAGK	AT3G57560	Plastid	Nucleotide metabolism	T51	44.63	74.82	-30.19
O82326*	AT2G14880	Plastid, Mitochondrion (GO)	-	T46	2.47	27.16	-24.69
MORF9	AT1G11430	Plastid	Development	T61	1.36	23.67	-22.31
ZEP	AT5G67030	Plastid	Xanthophyll biosynthetic process (GO)	A61	1.56	23.23	-21.69

Supplemental Table 3. Comparison of downregulated NTA target sites shared in plants of the *gnat1* and the *gnat2* background. The yield differences in NTA were calculated by subtracting the NTA yield determined for *gnat1* from the yield defined for *gnat2*. The NTA profiles of the *gnat2* mutant lines were previously generated by Bienvenut and coworkers as well as by Eirich and coworkers (26, 84). The global analysis is presented in the Suppl. Data 3 and plots of NTA yield differences are shown in Suppl. Fig. 5. The position of NTA within the corresponding target protein sequence is given as a combination of number and amino acid residue in a one-letter code. Subcellular localizations were assigned using SUBA4, unless otherwise stated, or in cases of unavailable or unclear classification by referring to the TAIR10 database. Classification of biological processes was assigned using MapMap, unless otherwise stated, or in cases of unavailable or unclear assignment by consulting the Gene Ontology resource (GO) or the UniProt database. Candidates without official or submitted names were marked with an asterisk and referred to by using their UniProt accession ID.

					gnat1	***gnat2***	**Difference**
Protein ID	Accession number (ARAPORT-11)	Subcellular localization	Biological process	Position of NTA site	NTA yield in *gnat1* (mean, %)	NTA yield in *gnat2* (mean, %)	*gnat1 - gnat2* (%, ± SE)
HC244	AT4G35250	Plastid	PSII assembly (GO)	S65	98.80	2.14	96.66 ± 0.62
TIC55	AT2G24820	Plastid	Protein targeting, chloroplast	A51	95.22	2.24	92.98 ± 1.02
PSNB2	AT1G64770	Plastid	Photosynthesis light reaction	S19	73.57	0.78	72.79 ± 0.88
PORB	AT4G27440	Plastid	Tetrapyrrole synthesis	T68	69.02	4.14	64.88 ± 0.96
ASSY	AT4G24830	Plastid	Amino acid metabolism	V75	92.67	29.01	63.66 ± 1.01
LHCA5	AT1G45474	Plastid	Photosynthesis light reaction	A33	65.91	4.96	60.95 ± 4.63
A0A178WL48*	AT1G16080	Plastid	-	A45	62.26	2.04	60.22 ± 0.90
NAGK	AT3G57560	Plastid	Nucleotide metabolism	T51	44.63	5.48	39.15 ± 1.65
LPA3	AT1G73060	Plastid	-	G25	31.03	6.41	24.62 ± 0.95
P25696*	AT4G36530	Plastid, Cytosol, Mitochondrion, Nucleus (GO)	-	S53	25.00	4.51	20.49 ± 1.16
