# Supplementary material for: The Plastidial Protein Acetyltransferase GNAT1 Forms a Complex With GNAT2, yet Their Interaction Is Dispensable for State Transitions
Source: Mol Cell Proteomics. 2024 Sep 28;23(11):100850. doi: 10.1016/j.mcpro.2024.100850 (PMC11585782; doi:10.1016/j.mcpro.2024.100850)
Supplement: Suppl. Table 4 [file mmc8.pdf]

Supplemental Table 4: Confidence score values calculated by AlphaFold 2 Multimer for homo-oligomer models of GNAT1, GNAT2, or GNAT3. The average quality of each multimer prediction is evaluated by the intrinsic model accuracy that estimates pTM and ipTM (predicted Template Modeling score and interface predicted Template Modeling score) as a value between 0-1 (43).

GNAT multimer	AF2 confidence (iptm+ptm)
GNAT1 homo-dimer	0.882
GNAT1 homo-trimer	0.545
GNAT1 homo-tetramer	0.460
GNAT1 homo-pentamer	0.322
GNAT1 homo-hexamer	0.290
GNAT1 homo-septamer	0.268
GNAT1 homo-octamer	0.260
GNAT2 homo-dimer	0.817
GNAT2 homo-trimer	0.568
GNAT2 homo-tetramer	0.393
GNAT2 homo-pentamer	0.319
GNAT2 homo-hexamer	0.299
GNAT2 homo-septamer	0.281
GNAT2 homo-octamer	0.275
GNAT3 homo-dimer	0.802
GNAT3 homo-trimer	0.381
GNAT3 homo-tetramer	0.358
GNAT3 homo-pentamer	0.287
GNAT3 homo-hexamer	0.280
GNAT3 homo-septamer	0.257
GNAT3 homo-octamer	0.262
