# Supplementary material for: The Plastidial Protein Acetyltransferase GNAT1 Forms a Complex With GNAT2, yet Their Interaction Is Dispensable for State Transitions
Source: Mol Cell Proteomics. 2024 Sep 28;23(11):100850. doi: 10.1016/j.mcpro.2024.100850 (PMC11585782; doi:10.1016/j.mcpro.2024.100850)

Supplemental Table 5. Analysis of photosynthetic performance of the Arabidopsis knockout lines *gnat1-1*, *gnat1-2* and *gnat2-1* in comparison to wild type (WT) plants. Photosystem I- and photosystem II-specific parameters were determined by measuring light response curves with a Dual-PAM-100 device (Walz). Thereby, chlorophyll *a*-fluorescence of photosystem II and the P700 signal of photosystem I (830 nm- and 875 nm-transmittance signals) were recorded simultaneously. In total, six replicates per line were analyzed.

F_v/F_m

WT		*gnat1-1*		*gnat1-2*		*gnat2-1*	
Average	St. Dev.	Average	St. Dev.	Average	St. Dev.	Average	St. Dev.
0.81	0.01	0.81	0.01	0.81	0.02	0.81	0.01

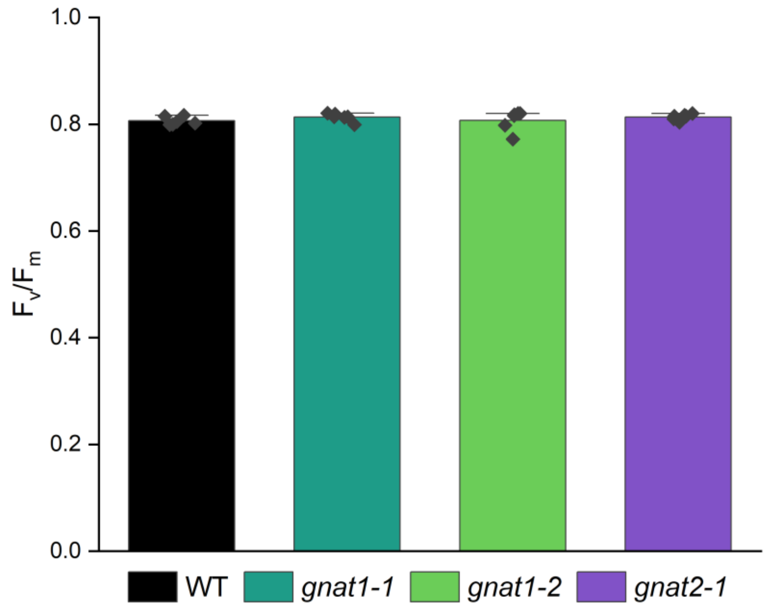

P_m

WT		*gnat1-1*		*gnat1-2*		*gnat2-1*	
Average	St. Dev.	Average	St. Dev.	Average	St. Dev.	Average	St. Dev.
0.84	0.10	0.94	0.07	0.91	0.09	1.02	0.06

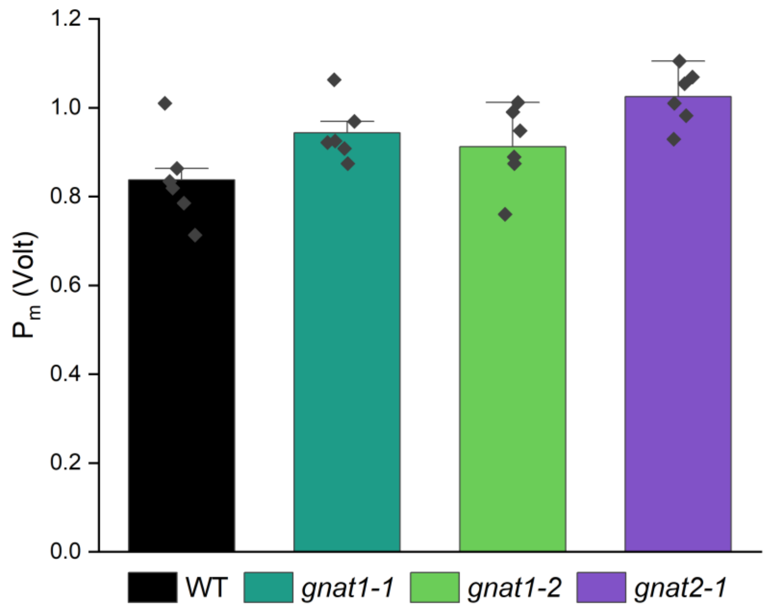

Y(II)

	WT		*gnat1-1*		*gnat1-2*		*gnat2-1*	
PAR (µE)	Average	St. Dev.	Average	St. Dev.	Average	St. Dev.	Average	St. Dev.
23	0.64	0.02	0.65	0.02	0.64	0.01	0.60	0.01
54	0.54	0.02	0.52	0.02	0.54	0.04	0.51	0.04
127	0.27	0.02	0.26	0.01	0.28	0.03	0.26	0.04
217	0.16	0.01	0.16	0.01	0.17	0.02	0.17	0.03
431	0.08	0.01	0.09	0.01	0.09	0.01	0.09	0.01
661	0.06	0.01	0.06	0.01	0.06	0.01	0.07	0.01
1029	0.04	0.01	0.04	0.01	0.05	0.01	0.05	0.00

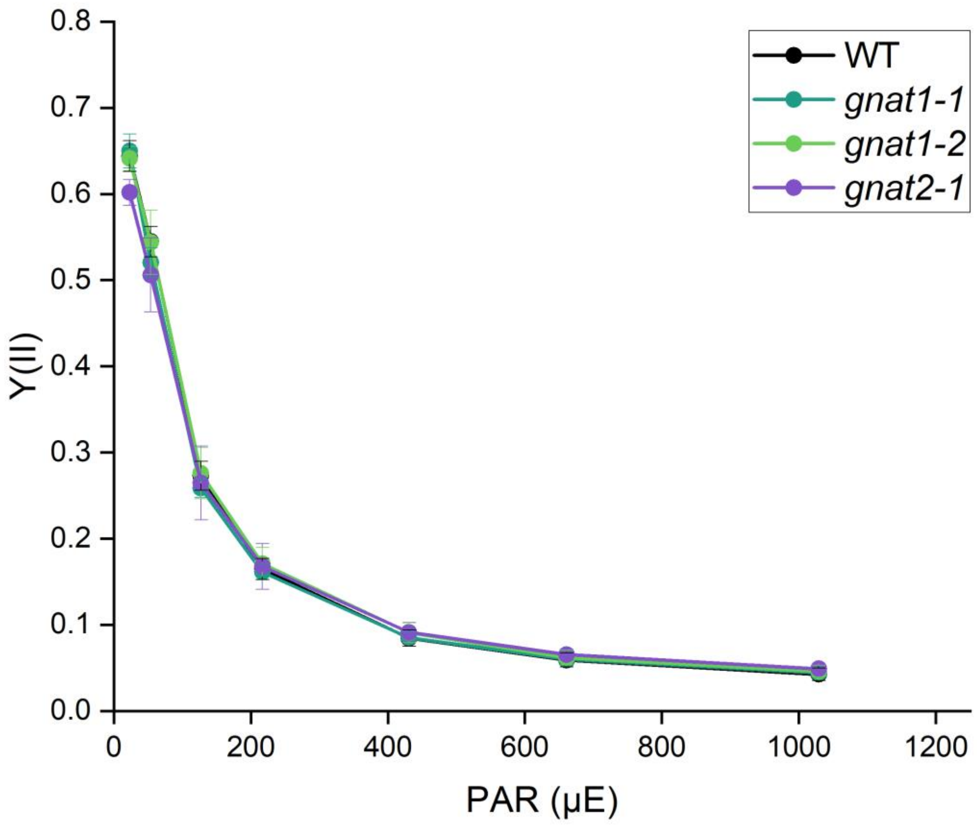

Y(NPQ)

	WT		*gnat1-1*		*gnat1-2*		*gnat2-1*	
PAR (µE)	Average	St. Dev.	Average	St. Dev.	Average	St. Dev.	Average	St. Dev.
23	0.07	0.02	0.07	0.03	0.06	0.02	0.06	0.01
54	0.20	0.03	0.23	0.03	0.19	0.05	0.19	0.07
127	0.45	0.02	0.47	0.01	0.45	0.03	0.45	0.05
217	0.53	0.01	0.54	0.01	0.53	0.02	0.54	0.03
431	0.59	0.01	0.59	0.01	0.59	0.02	0.61	0.02
661	0.62	0.01	0.62	0.02	0.61	0.02	0.63	0.01
1029	0.64	0.01	0.64	0.02	0.63	0.01	0.65	0.01

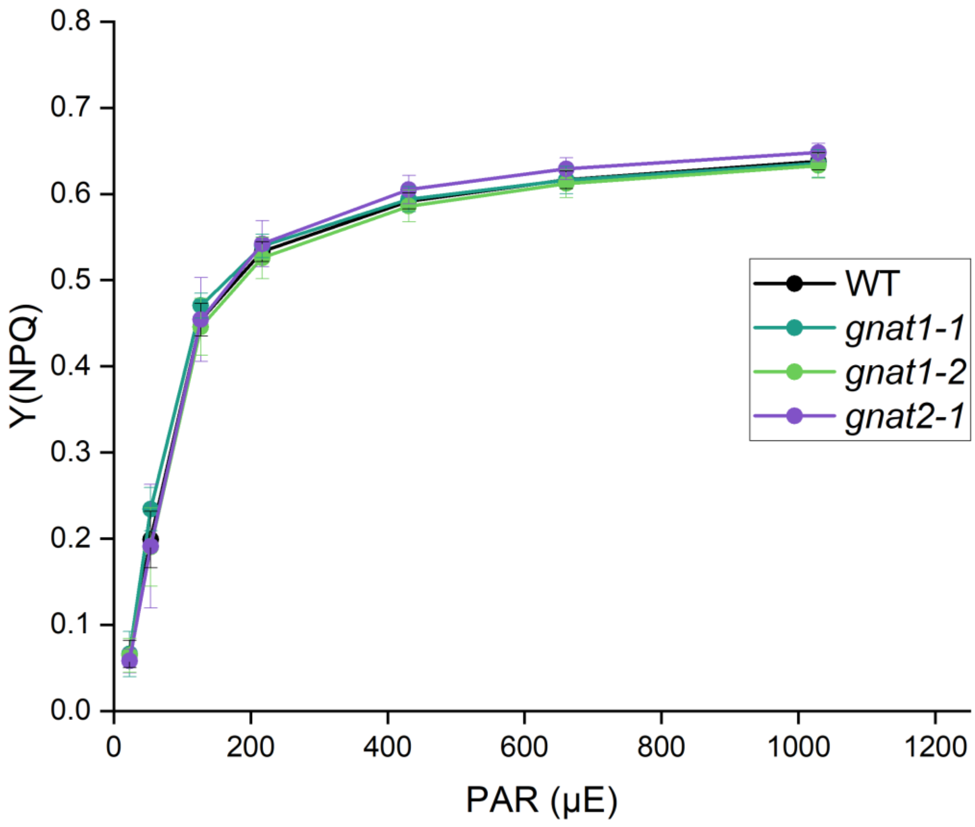

Y(NO)

	WT		*gnat1-1*		*gnat1-2*		*gnat2-1*	
PAR (µE)	Average	St. Dev.	Average	St. Dev.	Average	St. Dev.	Average	St. Dev.
23	0.29	0.00	0.28	0.01	0.29	0.01	0.34	0.03
54	0.26	0.02	0.25	0.01	0.27	0.02	0.28	0.04
127	0.27	0.01	0.27	0.01	0.28	0.02	0.27	0.03
217	0.30	0.01	0.30	0.01	0.30	0.01	0.29	0.02
431	0.32	0.01	0.32	0.01	0.32	0.01	0.30	0.02
661	0.32	0.01	0.32	0.01	0.33	0.01	0.31	0.02
1029	0.32	0.01	0.32	0.01	0.32	0.01	0.30	0.01

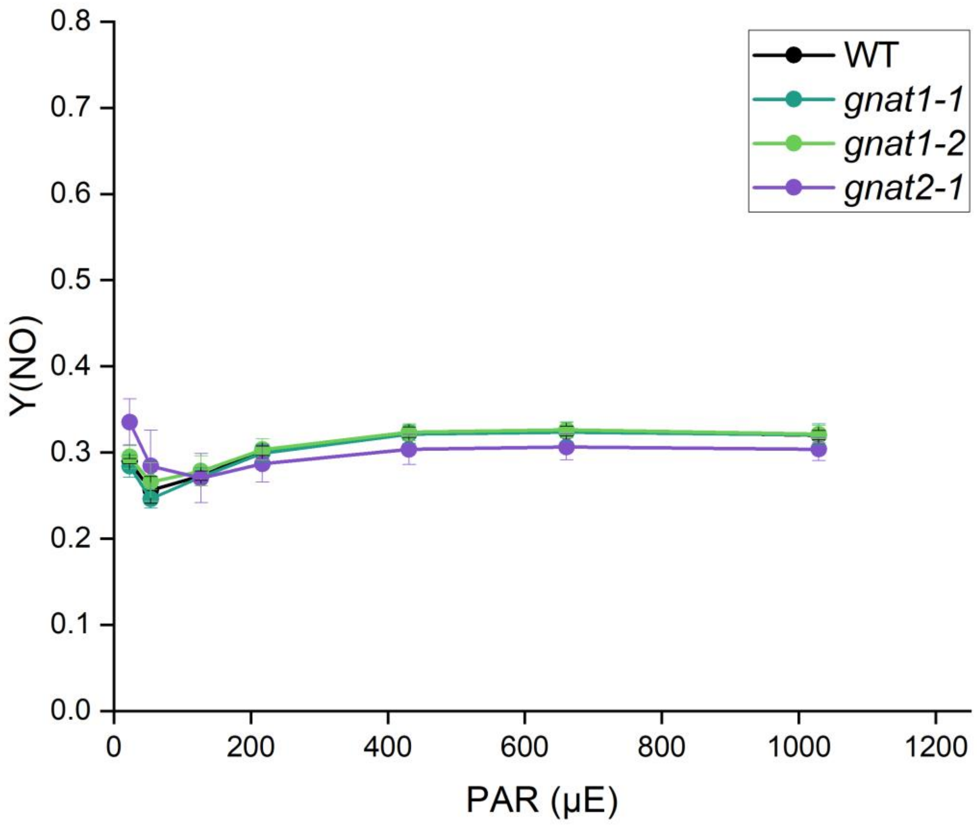

NPQ

	WT		*gnat1-1*		*gnat1-2*		*gnat2-1*	
PAR (µE)	Average	St. Dev.	Average	St. Dev.	Average	St. Dev.	Average	St. Dev.
23	0.23	0.05	0.24	0.10	0.22	0.07	0.18	0.05
54	0.78	0.17	0.95	0.14	0.73	0.21	0.71	0.34
127	1.66	0.09	1.74	0.11	1.61	0.18	1.71	0.30
217	1.77	0.05	1.81	0.10	1.74	0.13	1.90	0.20
431	1.83	0.05	1.85	0.10	1.81	0.10	2.00	0.16
661	1.90	0.06	1.91	0.11	1.88	0.10	2.06	0.14
1029	1.99	0.06	1.99	0.12	1.97	0.10	2.14	0.12

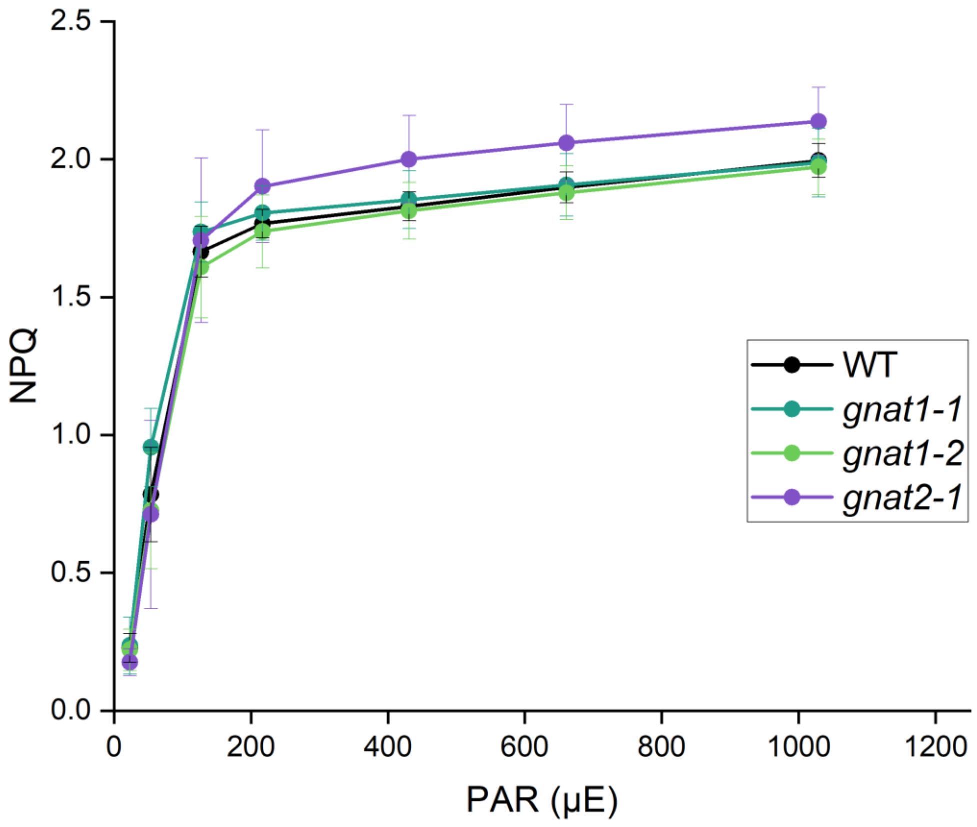

qN

	WT		*gnat1-1*		*gnat1-2*		*gnat2-1*	
PAR (µE)	Average	St. Dev.	Average	St. Dev.	Average	St. Dev.	Average	St. Dev.
23	0.22	0.04	0.22	0.08	0.21	0.06	0.17	0.04
54	0.51	0.06	0.57	0.04	0.48	0.08	0.46	0.14
127	0.72	0.01	0.73	0.02	0.71	0.03	0.72	0.05
217	0.73	0.01	0.73	0.01	0.73	0.02	0.74	0.03
431	0.74	0.01	0.74	0.01	0.74	0.01	0.76	0.02
661	0.75	0.01	0.75	0.01	0.75	0.01	0.76	0.02
1029	0.76	0.01	0.76	0.01	0.76	0.01	0.77	0.01

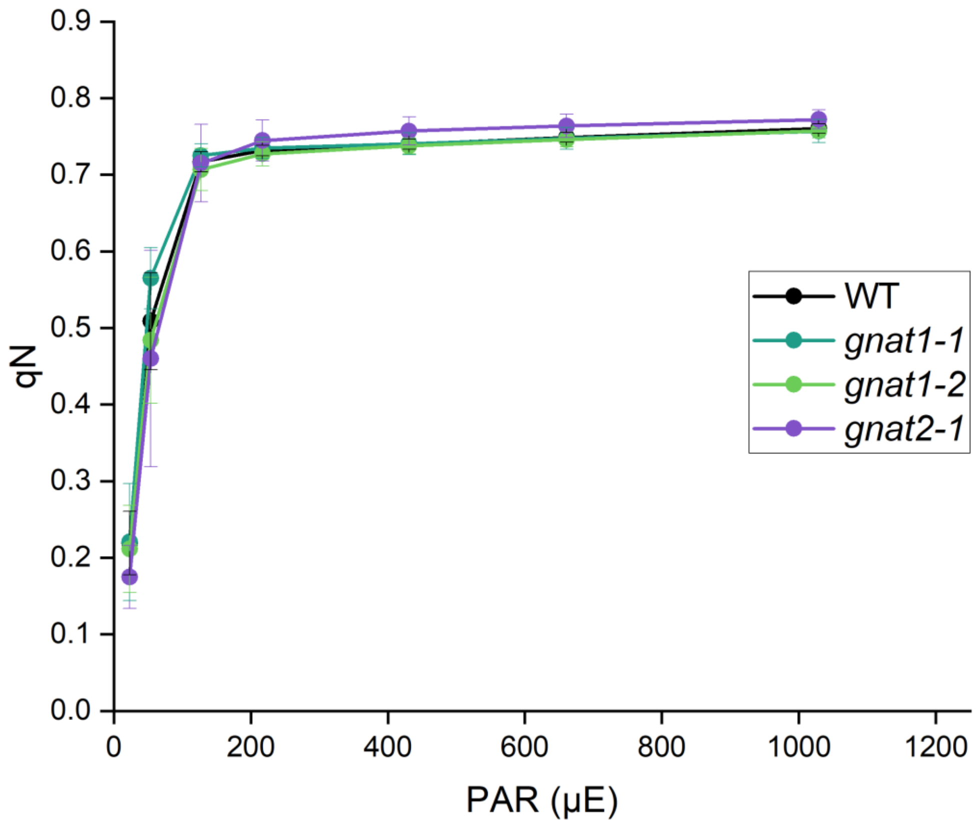

qP

	WT		*gnat1-1*		*gnat1-2*		*gnat2-1*	
PAR (µE)	Average	St. Dev.	Average	St. Dev.	Average	St. Dev.	Average	St. Dev.
23	0.84	0.02	0.84	0.01	0.83	0.01	0.77	0.03
54	0.78	0.01	0.76	0.01	0.77	0.02	0.73	0.03
127	0.45	0.03	0.43	0.02	0.45	0.04	0.45	0.05
217	0.28	0.02	0.27	0.01	0.29	0.03	0.29	0.04
431	0.14	0.02	0.14	0.01	0.15	0.02	0.16	0.02
661	0.10	0.02	0.10	0.01	0.11	0.01	0.11	0.02
1029	0.07	0.01	0.08	0.01	0.08	0.01	0.08	0.01

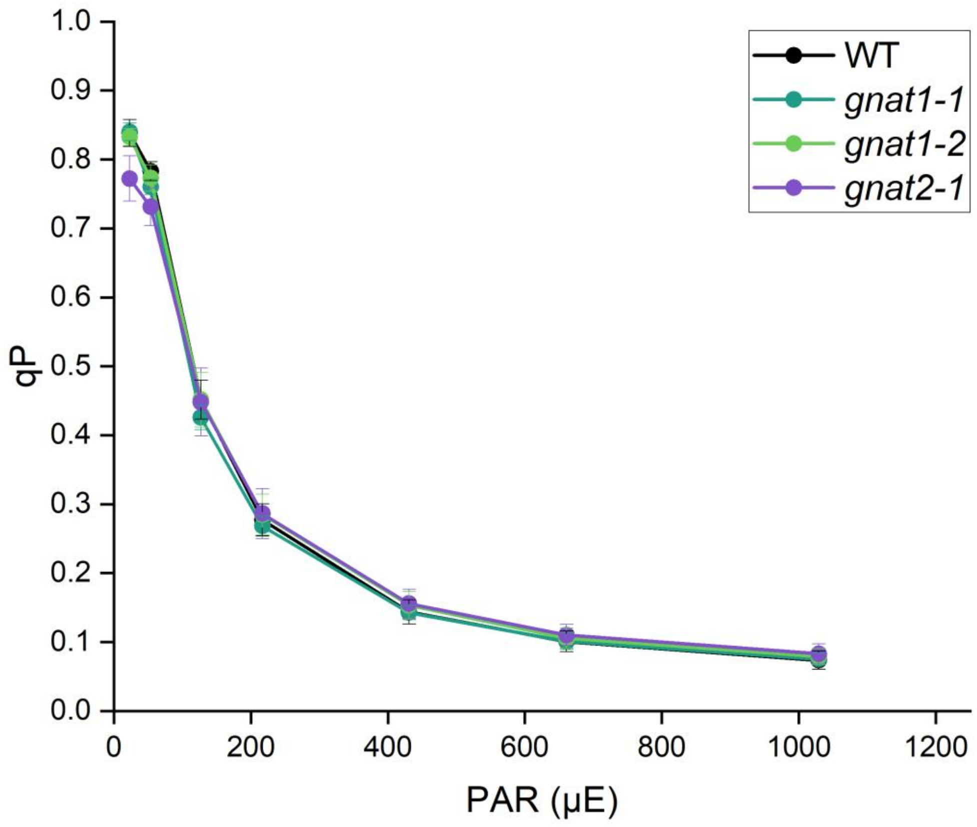

qL

	WT		gnat1-1		gnat1-2		gnat2-1	
PAR (µE)	Average	St. Dev.	Average	St. Dev.	Average	St. Dev.	Average	St. Dev.
23	0.55	0.04	0.54	0.02	0.53	0.04	0.42	0.05
54	0.52	0.02	0.50	0.02	0.50	0.02	0.43	0.05
127	0.25	0.02	0.23	0.02	0.24	0.03	0.24	0.04
217	0.13	0.02	0.13	0.01	0.14	0.02	0.14	0.02
431	0.06	0.01	0.06	0.01	0.07	0.01	0.07	0.01
661	0.04	0.01	0.04	0.01	0.05	0.01	0.05	0.01
1029	0.03	0.01	0.03	0.00	0.03	0.01	0.04	0.01

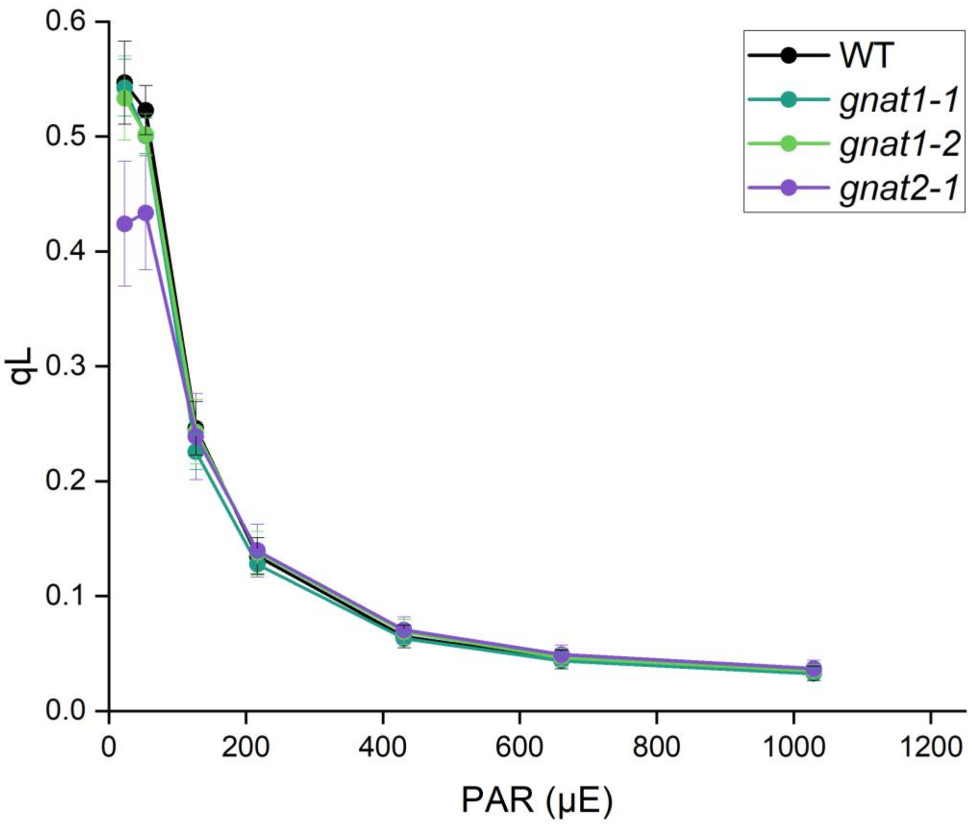

ETR(II)

	WT		*gnat1-1*		*gnat1-2*		*gnat2-1*	
PAR (µE)	Average	St. Dev.	Average	St. Dev.	Average	St. Dev.	Average	St. Dev.
23	6.22	0.17	6.27	0.20	6.18	0.15	5.88	0.22
54	12.37	0.44	11.82	0.40	12.33	0.86	11.87	0.81
127	14.55	0.90	13.80	0.62	14.72	1.57	14.68	1.82
217	15.03	1.09	14.68	0.84	15.57	1.72	15.62	1.94
431	15.32	1.68	15.42	1.17	16.42	2.11	16.58	2.03
661	16.35	2.29	16.68	2.09	17.18	2.42	17.85	2.32
1029	18.32	3.04	19.15	2.28	19.83	2.78	20.73	3.38

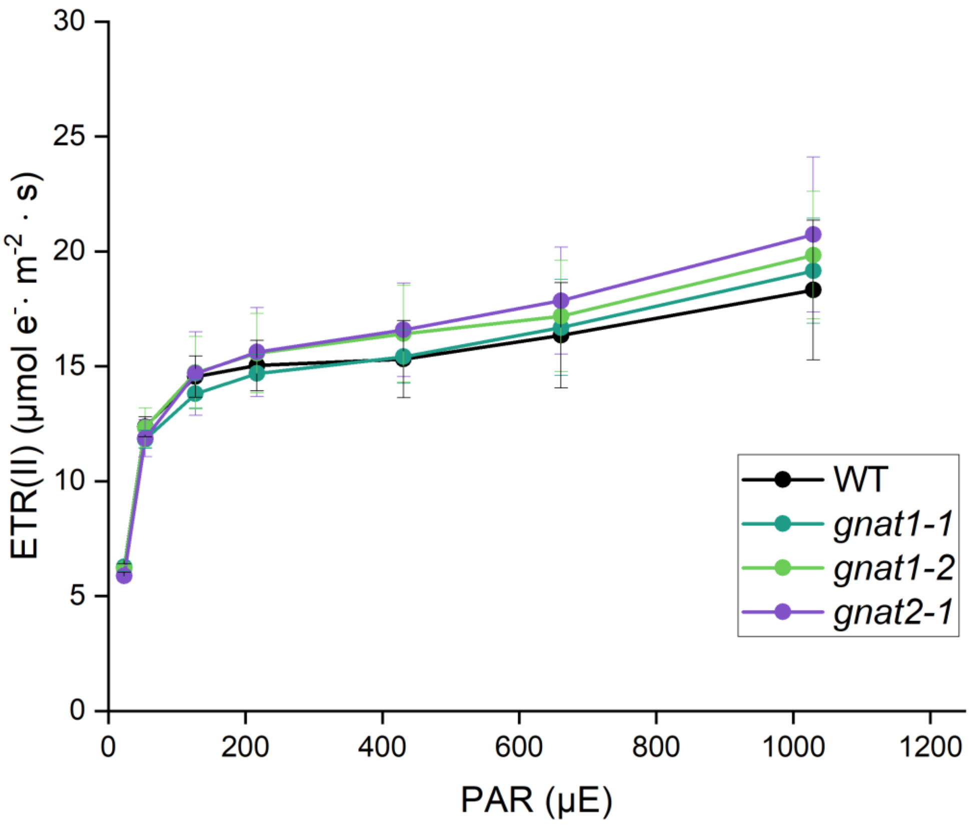

F/F_m

	WT		*gnat1-1*		*gnat1-2*		*gnat2-1*	
PAR (µE)	Average	St. Dev.	Average	St. Dev.	Average	St. Dev.	Average	St. Dev.
23	0.29	0.00	0.28	0.01	0.29	0.01	0.34	0.03
54	0.26	0.02	0.25	0.01	0.27	0.02	0.28	0.04
127	0.27	0.01	0.27	0.01	0.28	0.02	0.27	0.03
217	0.30	0.01	0.30	0.01	0.30	0.01	0.29	0.02
431	0.32	0.01	0.32	0.01	0.32	0.01	0.30	0.02
661	0.32	0.01	0.32	0.01	0.33	0.01	0.31	0.02
1029	0.32	0.01	0.32	0.01	0.32	0.01	0.30	0.01

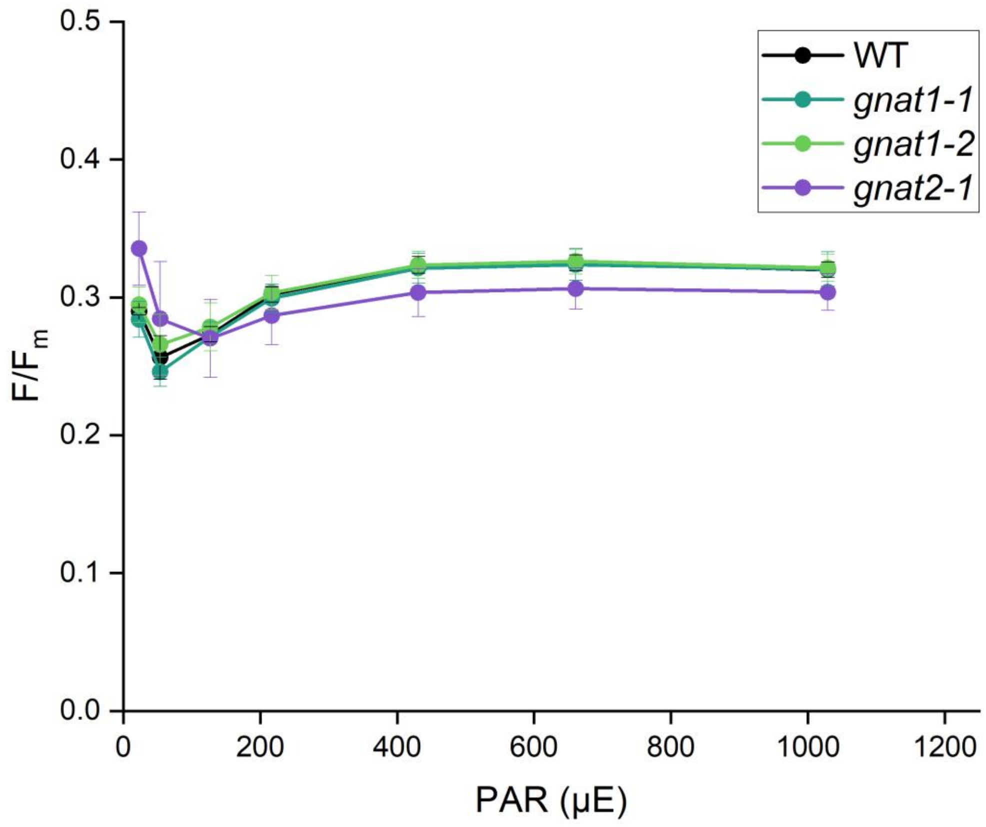

$F_{m'}/F_m$

	WT		*gnat1-1*		*gnat1-2*		*gnat2-1*	
PAR (µE)	Average	St. Dev.	Average	St. Dev.	Average	St. Dev.	Average	St. Dev.
23	0.82	0.04	0.81	0.06	0.82	0.05	0.85	0.04
54	0.57	0.06	0.51	0.04	0.59	0.07	0.60	0.12
127	0.38	0.01	0.37	0.01	0.39	0.03	0.37	0.05
217	0.36	0.01	0.36	0.01	0.37	0.02	0.35	0.03
431	0.35	0.01	0.35	0.01	0.36	0.01	0.33	0.02
661	0.35	0.01	0.34	0.01	0.35	0.01	0.33	0.01
1029	0.33	0.01	0.34	0.01	0.34	0.01	0.32	0.01

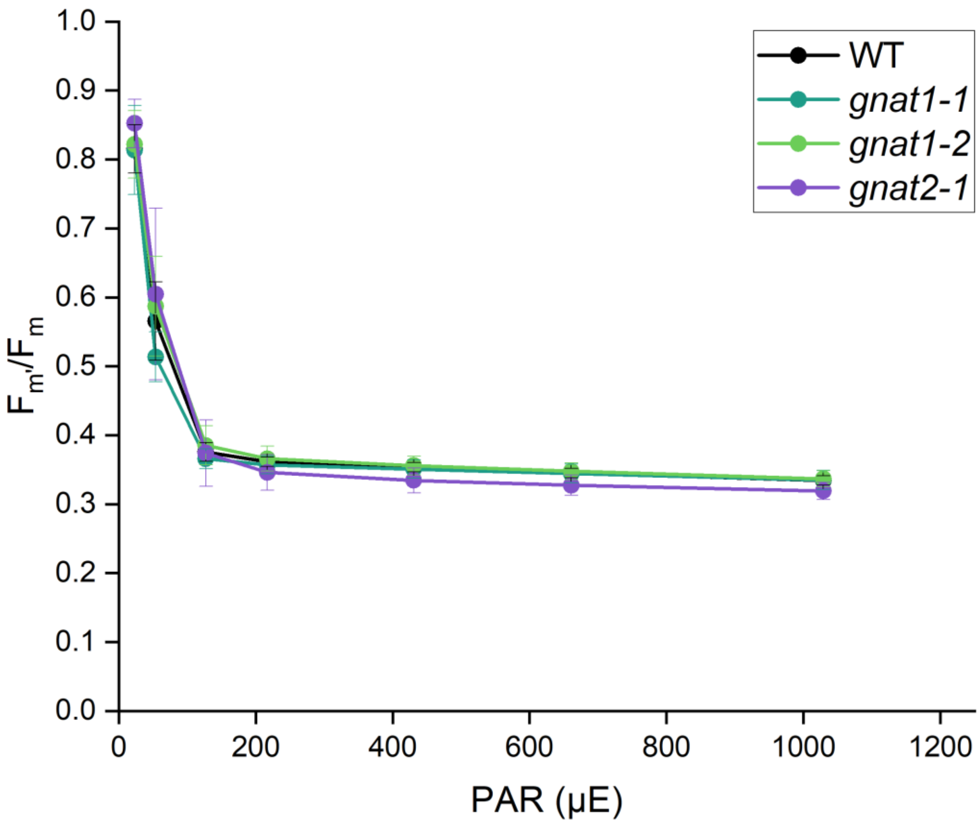

$F_{0'}/F_0$

	WT		*gnat1-1*		*gnat1-2*		*gnat2-1*	
PAR (µE)	Average	St. Dev.	Average	St. Dev.	Average	St. Dev.	Average	St. Dev.
23	0.96	0.01	0.96	0.02	0.96	0.01	0.97	0.01
54	0.87	0.02	0.85	0.02	0.88	0.03	0.88	0.05
127	0.75	0.01	0.75	0.01	0.76	0.02	0.76	0.03
217	0.74	0.01	0.74	0.01	0.75	0.01	0.74	0.02
431	0.73	0.01	0.74	0.01	0.74	0.01	0.73	0.02
661	0.73	0.01	0.73	0.01	0.73	0.01	0.72	0.01
1029	0.72	0.01	0.72	0.01	0.72	0.01	0.71	0.01

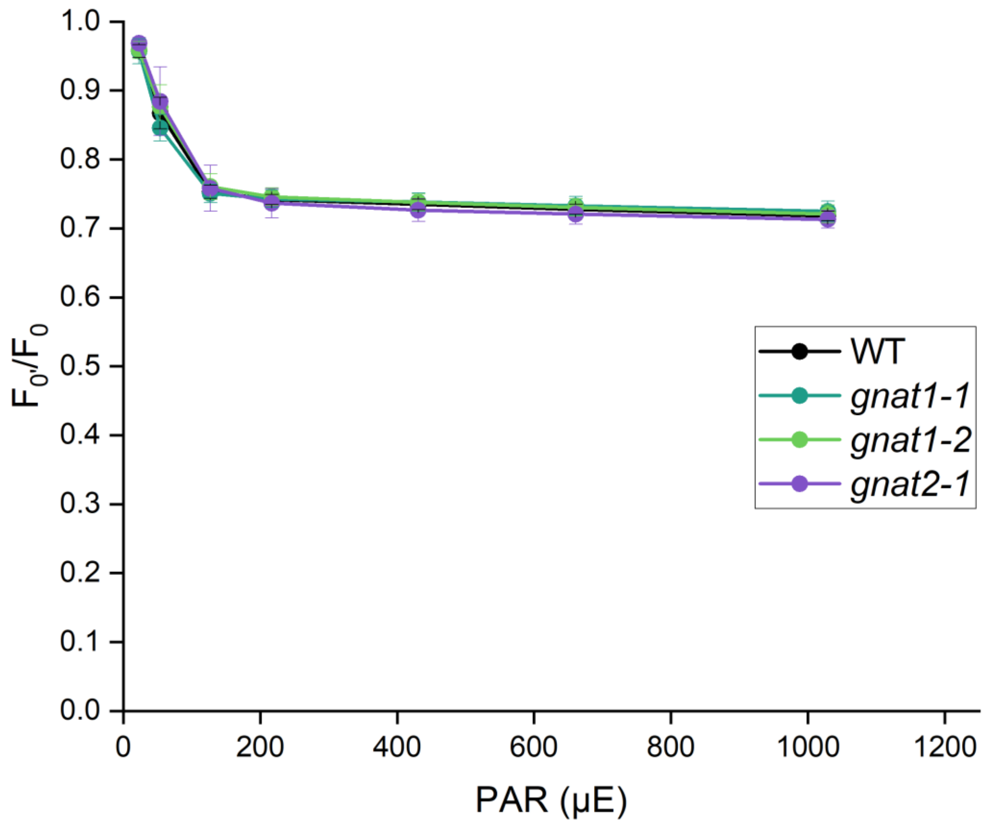

F_0, $F_{0'}$

	WT		*gnat1-1*		*gnat1-2*		*gnat2-1*	
PAR (µE)	Average	St. Dev.	Average	St. Dev.	Average	St. Dev.	Average	St. Dev.
0	0.16	0.02	0.17	0.01	0.17	0.01	0.18	0.01
23	0.15	0.02	0.17	0.01	0.16	0.01	0.18	0.01
54	0.14	0.02	0.15	0.01	0.15	0.01	0.16	0.01
127	0.12	0.02	0.13	0.01	0.13	0.01	0.14	0.01
217	0.12	0.02	0.13	0.01	0.13	0.01	0.14	0.01
431	0.12	0.02	0.13	0.01	0.12	0.01	0.13	0.01
661	0.12	0.02	0.13	0.01	0.12	0.01	0.13	0.01
1029	0.12	0.02	0.13	0.01	0.12	0.01	0.13	0.01

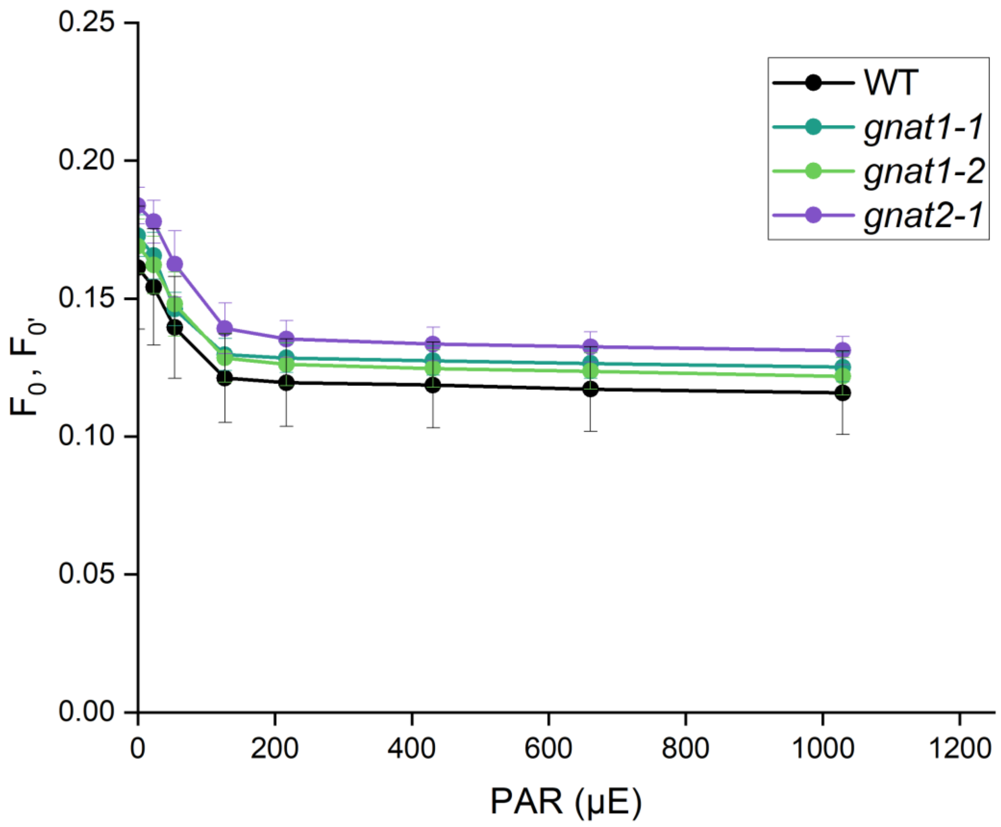

F_m, F_m'

	WT		*gnat1-1*		*gnat1-2*		*gnat2-1*	
PAR (µE)	Average	St. Dev.	Average	St. Dev.	Average	St. Dev.	Average	St. Dev.
0	0.82	0.12	0.90	0.03	0.86	0.05	0.97	0.03
23	0.67	0.10	0.74	0.07	0.71	0.06	0.83	0.05
54	0.46	0.06	0.46	0.03	0.51	0.08	0.59	0.13
127	0.31	0.04	0.33	0.02	0.33	0.03	0.36	0.05
217	0.30	0.04	0.32	0.02	0.31	0.02	0.34	0.03
431	0.29	0.04	0.32	0.02	0.31	0.02	0.33	0.02
661	0.28	0.04	0.31	0.02	0.30	0.02	0.32	0.02
1029	0.27	0.04	0.30	0.02	0.29	0.02	0.31	0.02

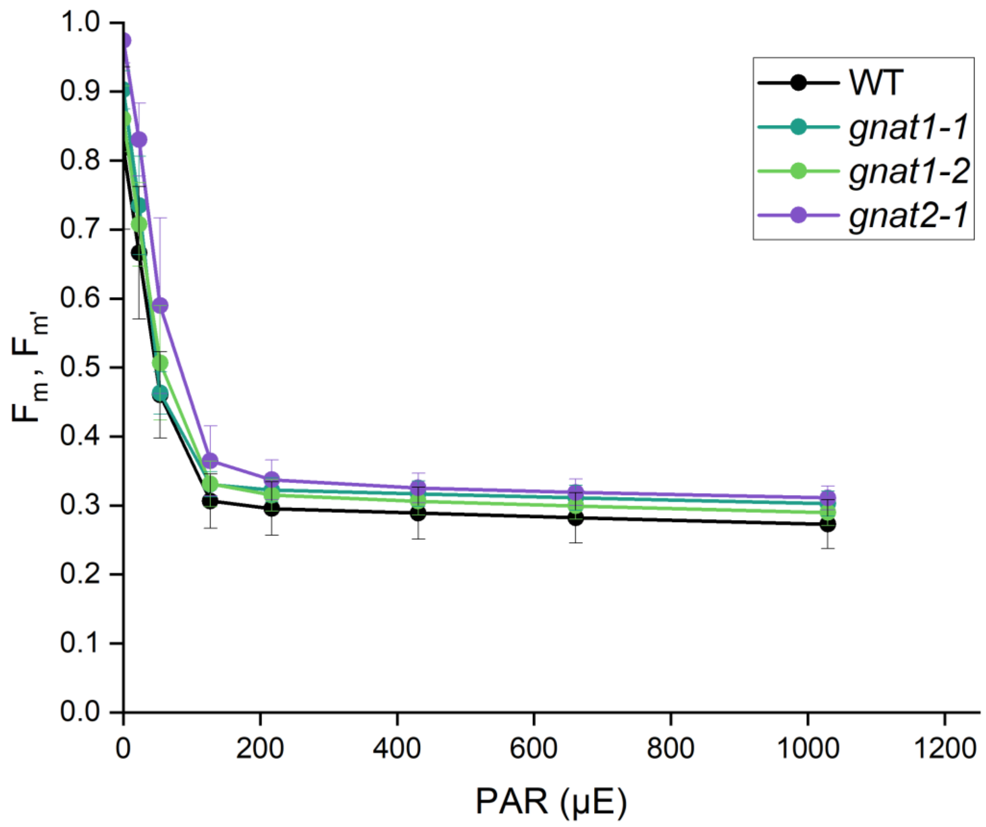

F

	WT		*gnat1-1*		*gnat1-2*		*gnat2-1*	
PAR (µE)	Average	St. Dev.	Average	St. Dev.	Average	St. Dev.	Average	St. Dev.
0	0.16	0.02	0.17	0.01	0.17	0.01	0.18	0.01
23	0.24	0.03	0.26	0.01	0.25	0.01	0.33	0.03
54	0.21	0.03	0.22	0.01	0.23	0.02	0.28	0.04
127	0.22	0.03	0.25	0.01	0.24	0.02	0.26	0.03
217	0.25	0.03	0.27	0.02	0.26	0.01	0.28	0.02
431	0.26	0.04	0.29	0.02	0.28	0.02	0.30	0.02
661	0.27	0.04	0.29	0.02	0.28	0.02	0.30	0.02
1029	0.26	0.03	0.29	0.02	0.28	0.02	0.30	0.02

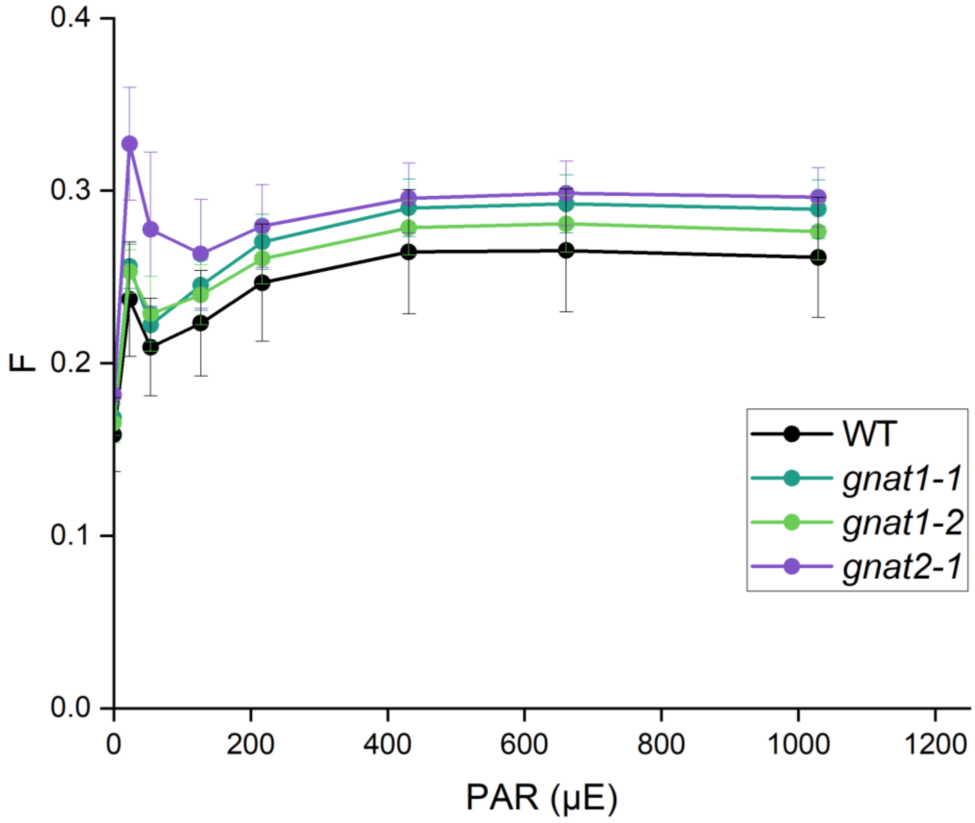

Y(I)

	WT		*gnat1-1*		*gnat1-2*		*gnat2-1*	
PAR (µE)	Average	St. Dev.	Average	St. Dev.	Average	St. Dev.	Average	St. Dev.
23	0.71	0.04	0.71	0.05	0.74	0.03	0.73	0.04
54	0.74	0.02	0.73	0.03	0.75	0.03	0.74	0.01
127	0.39	0.03	0.39	0.02	0.43	0.04	0.44	0.05
217	0.24	0.03	0.24	0.01	0.27	0.04	0.28	0.04
431	0.13	0.03	0.12	0.01	0.14	0.02	0.15	0.03
661	0.09	0.03	0.09	0.02	0.10	0.02	0.11	0.02
1029	0.05	0.03	0.07	0.01	0.08	0.02	0.07	0.02

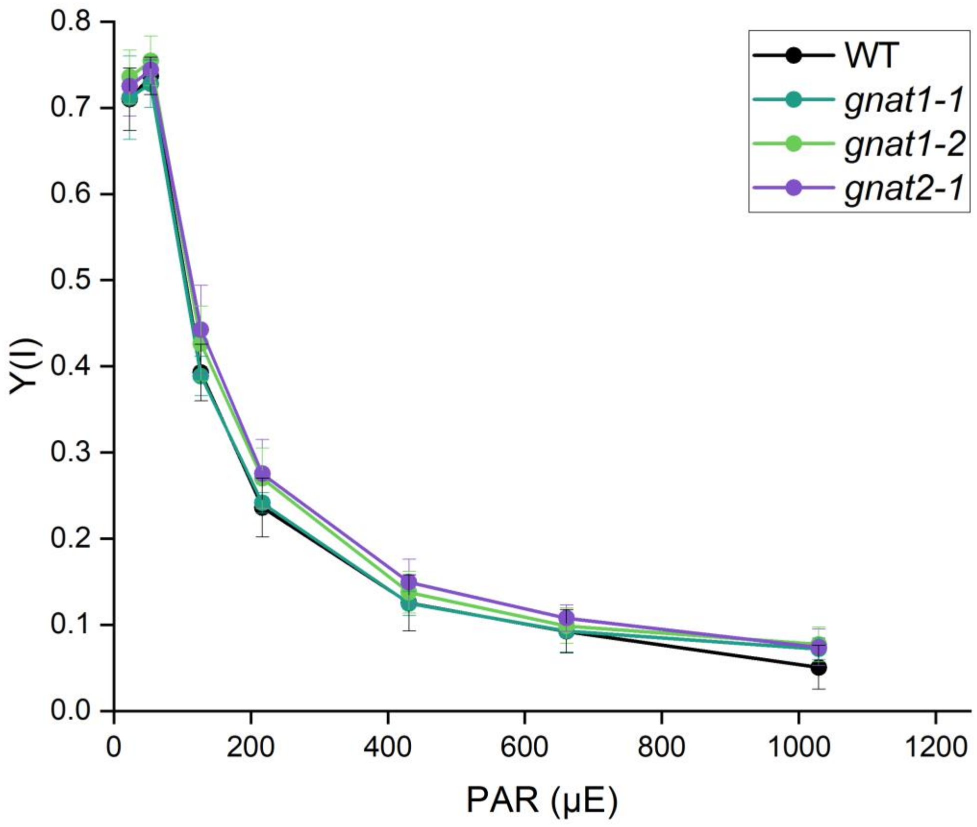

Y(ND)

	WT		*gnat1-1*		*gnat1-2*		*gnat2-1*	
PAR (µE)	Average	St. Dev.	Average	St. Dev.	Average	St. Dev.	Average	St. Dev.
23	0.02	0.02	0.01	0.02	0.01	0.02	0.00	0.00
54	0.14	0.02	0.16	0.03	0.13	0.04	0.09	0.06
127	0.50	0.02	0.51	0.02	0.49	0.04	0.46	0.07
217	0.64	0.03	0.64	0.02	0.64	0.03	0.61	0.05
431	0.75	0.02	0.75	0.01	0.74	0.02	0.73	0.04
661	0.78	0.04	0.76	0.02	0.78	0.03	0.76	0.04
1029	0.79	0.03	0.77	0.01	0.80	0.03	0.76	0.05

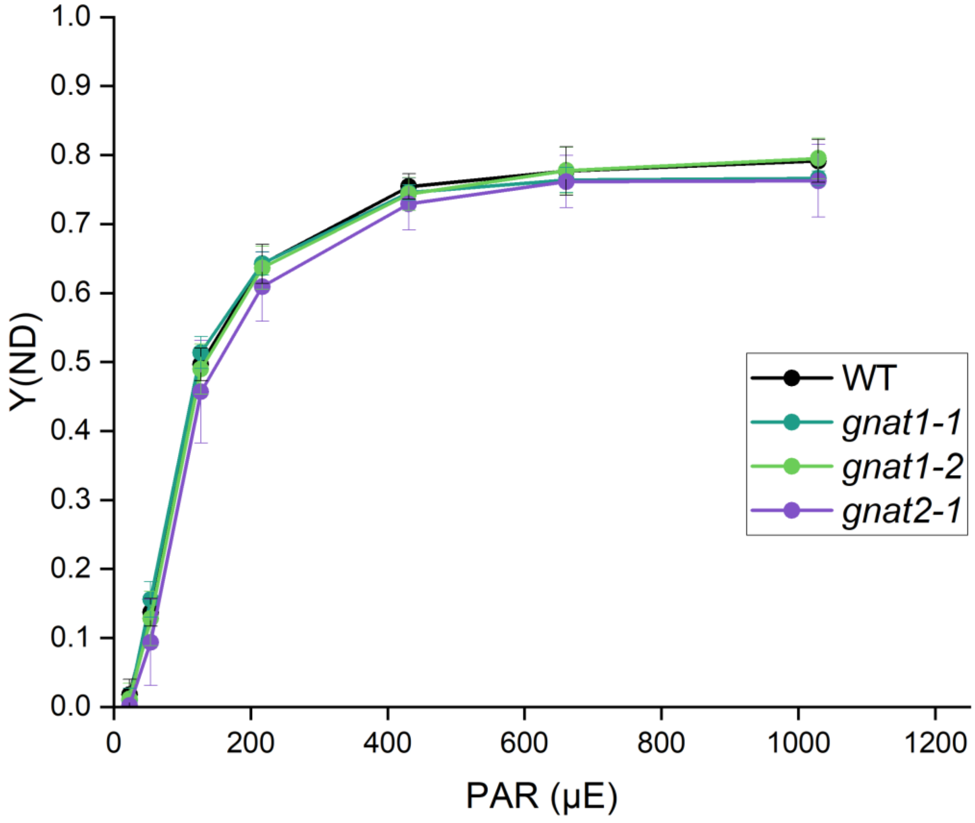

Y(NA)

	WT		*gnat1-1*		*gnat1-2*		*gnat2-1*	
PAR (µE)	Average	St. Dev.	Average	St. Dev.	Average	St. Dev.	Average	St. Dev.
23	0.27	0.04	0.28	0.05	0.25	0.05	0.27	0.03
54	0.13	0.04	0.12	0.04	0.12	0.04	0.16	0.06
127	0.11	0.02	0.10	0.02	0.08	0.02	0.10	0.03
217	0.12	0.03	0.12	0.02	0.09	0.02	0.12	0.01
431	0.12	0.03	0.13	0.01	0.12	0.03	0.12	0.02
661	0.13	0.03	0.14	0.04	0.12	0.03	0.13	0.02
1029	0.16	0.03	0.16	0.02	0.13	0.02	0.16	0.03

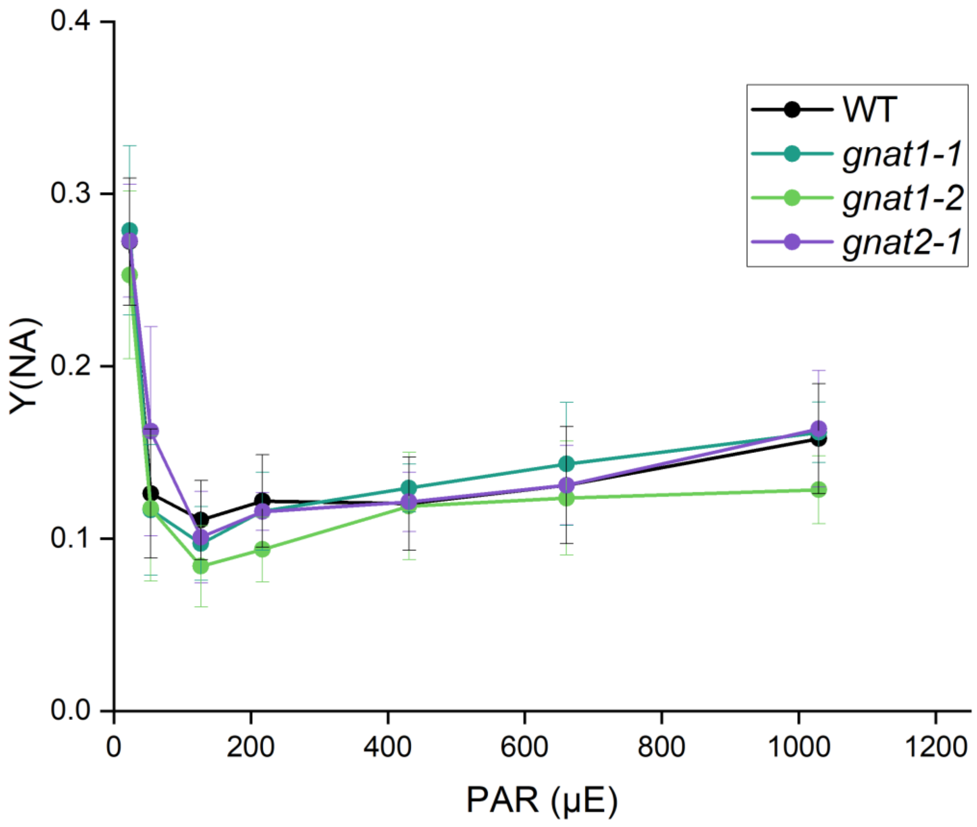

ETR(I)

	WT		*gnat1-1*		*gnat1-2*		*gnat2-1*	
PAR (µE)	Average	St. Dev.	Average	St. Dev.	Average	St. Dev.	Average	St. Dev.
23	6.85	0.34	6.87	0.48	7.10	0.30	7.00	0.35
54	16.70	0.49	16.50	0.62	17.10	0.64	16.87	0.29
127	20.92	1.77	20.75	1.22	22.72	2.32	23.58	2.72
217	21.50	3.13	21.98	1.11	24.58	3.17	25.08	3.64
431	22.65	5.84	22.62	2.49	24.90	4.39	27.08	4.84
661	25.62	6.93	25.75	6.82	27.38	5.75	29.85	4.35
1029	21.82	11.04	31.07	5.28	33.28	8.65	31.95	8.91

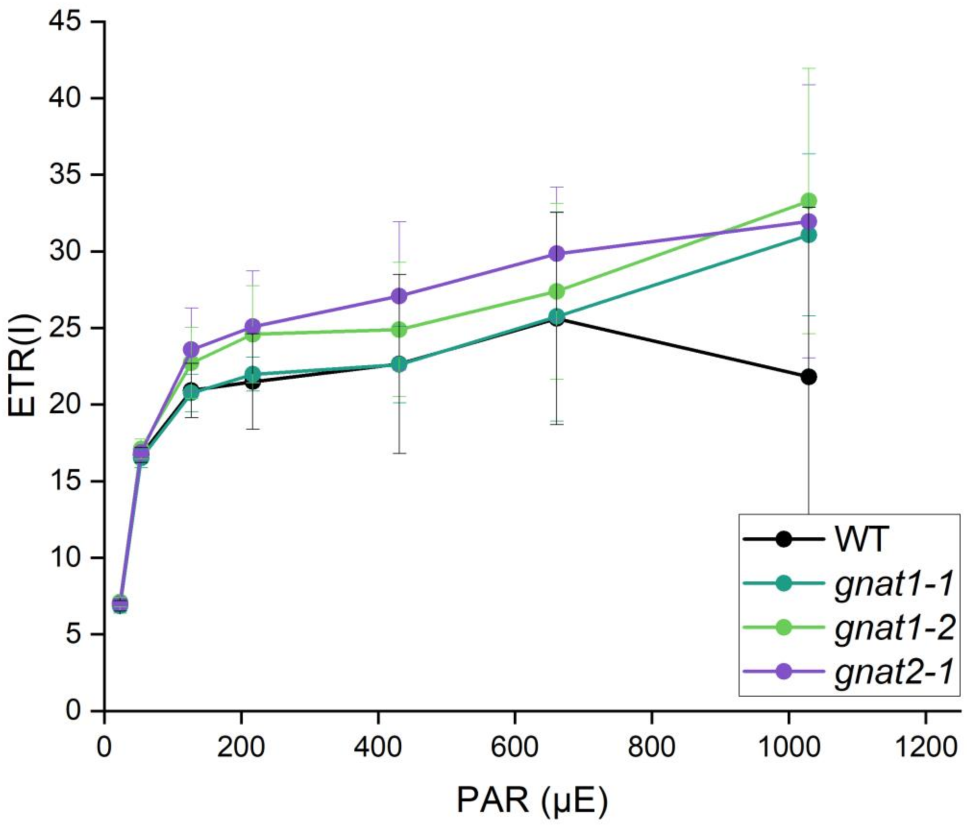

$P_{m'}$

	WT		*gnat1-1*		*gnat1-2*		*gnat2-1*	
PAR (µE)	Average	St. Dev.	Average	St. Dev.	Average	St. Dev.	Average	St. Dev.
23	0.61	0.09	0.67	0.06	0.68	0.11	0.74	0.06
54	0.73	0.10	0.83	0.07	0.81	0.09	0.86	0.10
127	0.75	0.10	0.85	0.06	0.84	0.09	0.92	0.08
217	0.74	0.09	0.83	0.08	0.83	0.09	0.91	0.06
431	0.74	0.10	0.82	0.07	0.81	0.10	0.90	0.06
661	0.73	0.11	0.81	0.08	0.80	0.09	0.89	0.06
1029	0.71	0.11	0.79	0.06	0.80	0.08	0.86	0.07

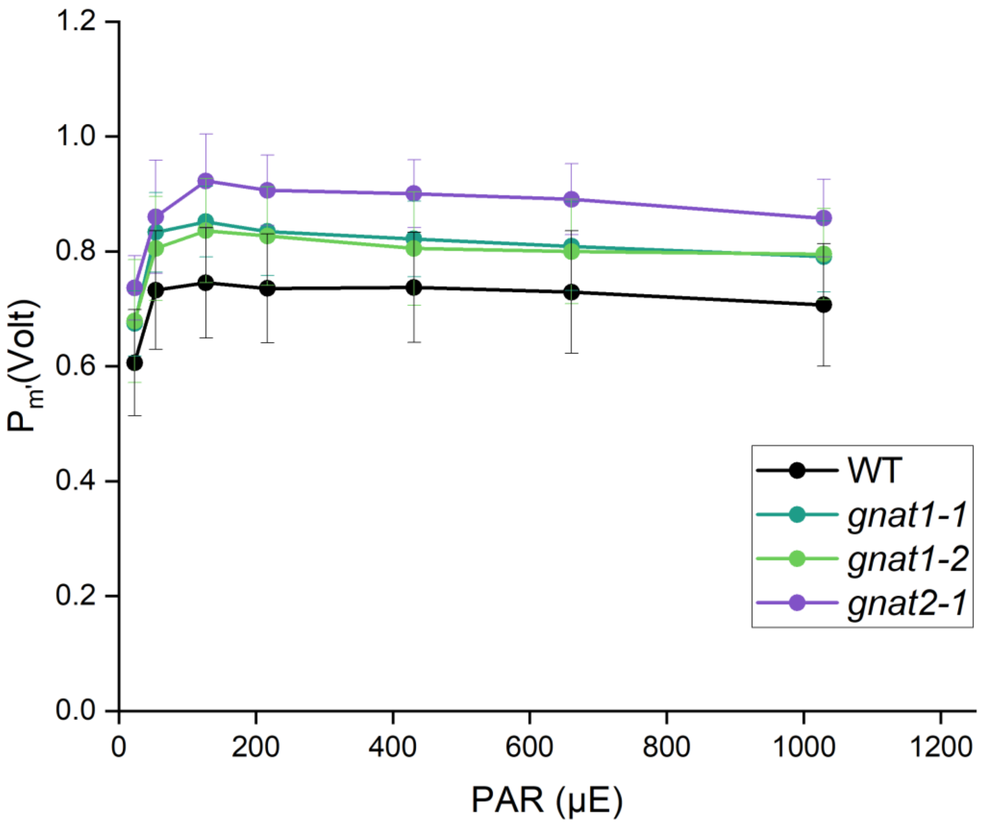

Supplement: Suppl. Table 5 [file mmc9.pdf]
