# Supplementary material for: The Plastidial Protein Acetyltransferase GNAT1 Forms a Complex With GNAT2, yet Their Interaction Is Dispensable for State Transitions
Source: Mol Cell Proteomics. 2024 Sep 28;23(11):100850. doi: 10.1016/j.mcpro.2024.100850 (PMC11585782; doi:10.1016/j.mcpro.2024.100850)
Supplement: Suppl. Table 6 [file mmc10.pdf]

Supplemental Table 6. Determination of photosynthesis performance parameters of the Arabidopsis knockout lines *gnat1-1*, *gnat1-2* and *gnat2-1* in comparison to wild type (WT) plants. Per treatment (GL, HL or D), three plates containing, each, seedlings of all genotypes were analyzed, whereby spots on 15 seedlings per plant line were selected. Chlorophyll *a* fluorescence of photosystem II was determined by using an Imaging-PAM device (Walz).

F_v/F_m

	WT		*gnat1-1*		*gnat1-2*		*gnat2-1*	
	Average	St. Dev.	Average	St. Dev.	Average	St. Dev.	Average	St. Dev.
GL	0.79	0.03	0.79	0.03	0.79	0.02	0.78	0.02
+ 1 day HL	0.75	0.01	0.75	0.01	0.75	0.01	0.63	0.08
+ 2 days HL	0.74	0.04	0.73	0.04	0.74	0.04	0.46	0.10
+ 3 days HL	0.72	0.06	0.70	0.09	0.72	0.06	0.39	0.14
+ 3 days D	0.61	0.05	0.62	0.05	0.63	0.05	0.60	0.05

Y(II)

**GL**	WT		*gnat1-1*		*gnat1-2*		*gnat2-1*	
PAR (µE)	Average	St. Dev.	Average	St. Dev.	Average	St. Dev.	Average	St. Dev.
50	0.492	0.018	0.504	0.019	0.501	0.024	0.452	0.020
124	0.445	0.019	0.457	0.022	0.454	0.025	0.401	0.020
451	0.229	0.018	0.227	0.022	0.232	0.030	0.225	0.019
962	0.103	0.013	0.108	0.018	0.105	0.015	0.115	0.018

**+ 1 day HL**	WT		*gnat1-1*		*gnat1-2*		*gnat2-1*	
PAR (µE)	Average	St. Dev.	Average	St. Dev.	Average	St. Dev.	Average	St. Dev.
50	0.427	0.027	0.430	0.024	0.432	0.022	0.301	0.075
124	0.410	0.024	0.417	0.022	0.413	0.024	0.263	0.069
451	0.247	0.021	0.257	0.019	0.258	0.019	0.151	0.060
962	0.145	0.018	0.145	0.020	0.153	0.013	0.092	0.032

**+ 2 days HL**	WT		*gnat1-1*		*gnat1-2*		*gnat2-1*	
PAR (µE)	Average	St. Dev.	Average	St. Dev.	Average	St. Dev.	Average	St. Dev.
50	0.425	0.045	0.434	0.042	0.423	0.052	0.193	0.069
124	0.440	0.041	0.447	0.048	0.438	0.052	0.174	0.077
451	0.276	0.035	0.281	0.041	0.278	0.044	0.081	0.069
962	0.152	0.033	0.163	0.033	0.167	0.034	0.054	0.043

**+ 3 days HL**	WT		*gnat1-1*		*gnat1-2*		*gnat2-1*	
PAR (µE)	Average	St. Dev.	Average	St. Dev.	Average	St. Dev.	Average	St. Dev.
50	0.410	0.090	0.374	0.142	0.387	0.082	0.164	0.088
124	0.461	0.082	0.432	0.122	0.433	0.073	0.157	0.097
451	0.292	0.074	0.286	0.092	0.279	0.053	0.072	0.079
962	0.164	0.048	0.164	0.062	0.161	0.041	0.047	0.049

**+ 3 days D**	WT		*gnat1-1*		*gnat1-2*		*gnat2-1*	
PAR (µE)	Average	St. Dev.	Average	St. Dev.	Average	St. Dev.	Average	St. Dev.
50	0.390	0.042	0.402	0.047	0.404	0.048	0.367	0.047
124	0.262	0.042	0.259	0.034	0.277	0.036	0.250	0.030
451	0.084	0.044	0.085	0.025	0.099	0.032	0.093	0.020
962	0.055	0.027	0.056	0.025	0.059	0.020	0.055	0.025

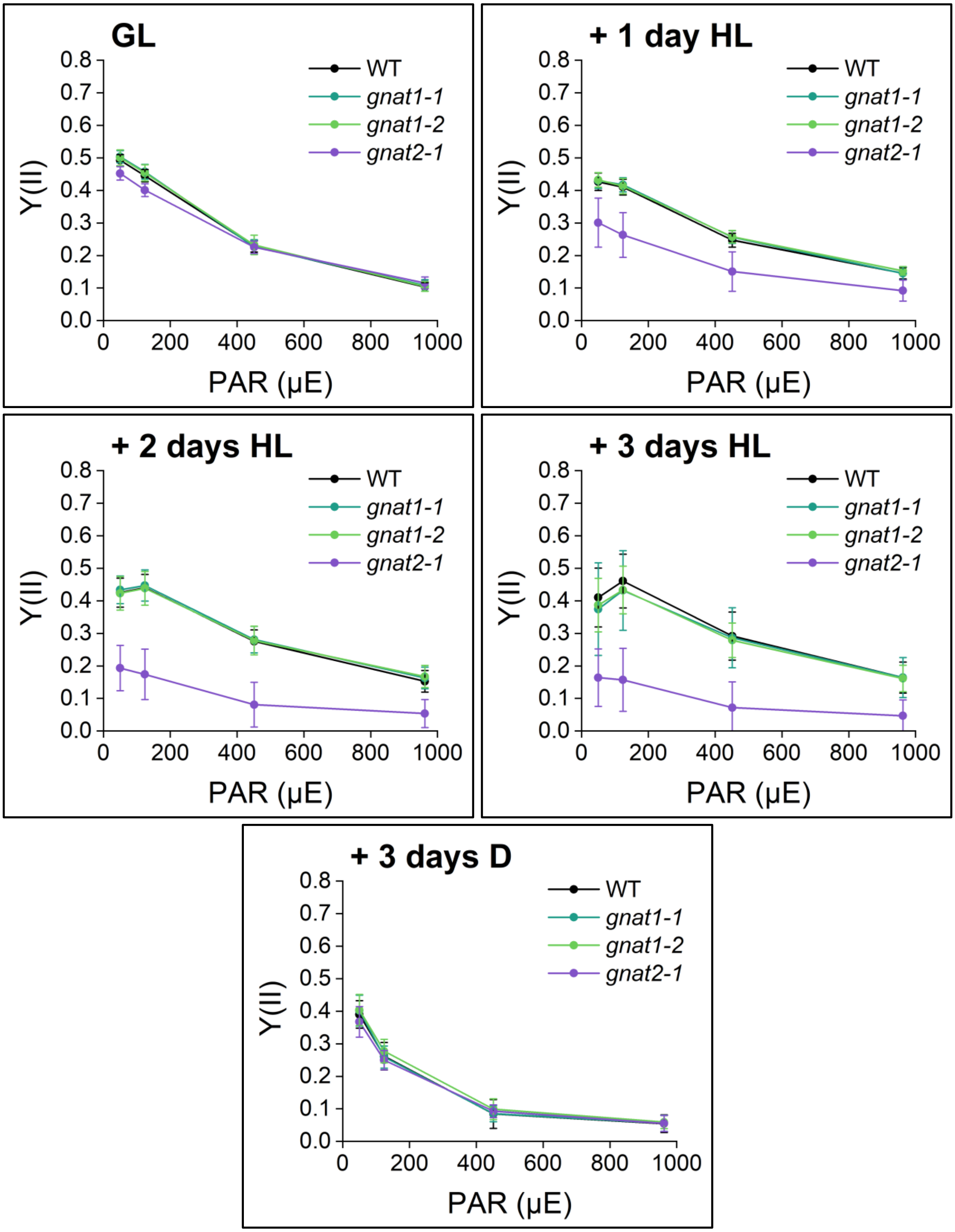

GL
+ 1 day HL
+ 2 days HL
+ 3 days HL
+ 3 days D
WT
gnat1-1
gnat1-2
gnat2-1
Y(II)
PAR (µE)
0.0
0.1
0.2
0.3
0.4
0.5
0.6
0.7
0.8
0
200
400
600
800
1000

Y(NPQ)

**GL**	WT		*gnat1-1*		*gnat1-2*		*gnat2-1*	
PAR (µE)	Average	St. Dev.	Average	St. Dev.	Average	St. Dev.	Average	St. Dev.
50	0.145	0.028	0.133	0.026	0.136	0.024	0.142	0.017
124	0.193	0.018	0.187	0.018	0.191	0.017	0.192	0.010
451	0.485	0.040	0.488	0.047	0.486	0.048	0.463	0.044
962	0.670	0.020	0.668	0.027	0.674	0.022	0.667	0.029

**+ 1 day HL**	WT		*gnat1-1*		*gnat1-2*		*gnat2-1*	
PAR (µE)	Average	St. Dev.	Average	St. Dev.	Average	St. Dev.	Average	St. Dev.
50	0.155	0.044	0.138	0.039	0.119	0.019	0.170	0.048
124	0.198	0.024	0.186	0.022	0.178	0.011	0.214	0.047
451	0.389	0.039	0.375	0.042	0.361	0.024	0.363	0.046
962	0.596	0.043	0.582	0.061	0.578	0.041	0.514	0.053

**+ 2 days HL**	WT		*gnat1-1*		*gnat1-2*		*gnat2-1*	
PAR (µE)	Average	St. Dev.	Average	St. Dev.	Average	St. Dev.	Average	St. Dev.
50	0.169	0.065	0.152	0.056	0.161	0.060	0.355	0.104
124	0.194	0.045	0.174	0.049	0.189	0.046	0.387	0.118
451	0.368	0.054	0.339	0.067	0.354	0.062	0.484	0.107
962	0.565	0.083	0.518	0.092	0.532	0.088	0.556	0.09

**+ 3 days HL**	WT		*gnat1-1*		*gnat1-2*		*gnat2-1*	
PAR (µE)	Average	St. Dev.	Average	St. Dev.	Average	St. Dev.	Average	St. Dev.
50	0.224	0.103	0.234	0.132	0.232	0.123	0.443	0.117
124	0.212	0.088	0.226	0.116	0.232	0.093	0.484	0.131
451	0.358	0.082	0.344	0.099	0.379	0.083	0.58	0.123
962	0.535	0.070	0.505	0.093	0.547	0.083	0.639	0.100

**+ 3 days D**	WT		*gnat1-1*		*gnat1-2*		*gnat2-1*	
PAR (µE)	Average	St. Dev.	Average	St. Dev.	Average	St. Dev.	Average	St. Dev.
50	0.180	0.031	0.173	0.041	0.173	0.032	0.158	0.020
124	0.275	0.042	0.271	0.044	0.273	0.038	0.254	0.036
451	0.480	0.050	0.476	0.043	0.478	0.038	0.452	0.032
962	0.582	0.035	0.576	0.032	0.578	0.032	0.557	0.028

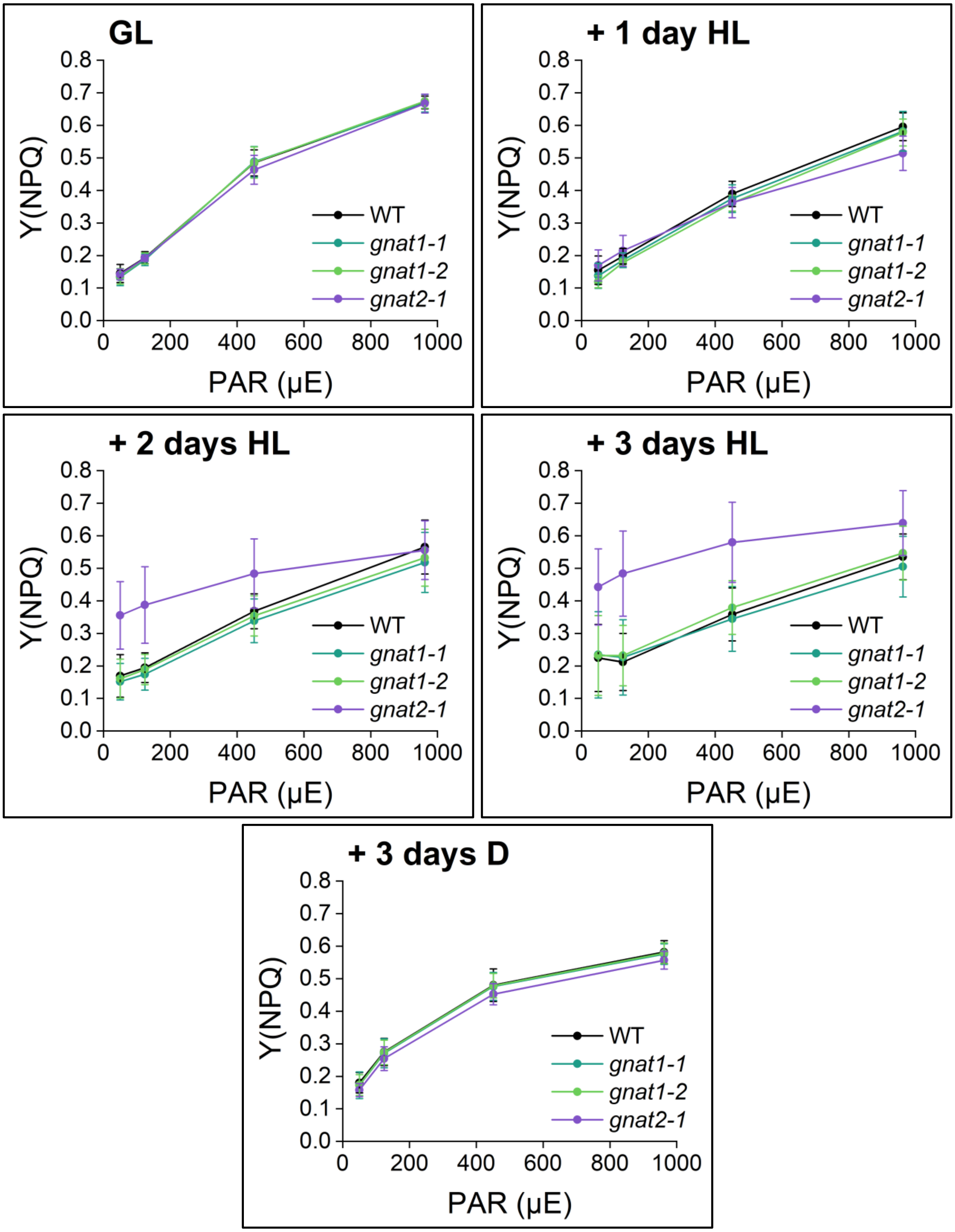

GL
+ 1 day HL
+ 2 days HL
+ 3 days HL
+ 3 days D
Y(NPQ)
PAR (µE)
0.0
0.1
0.2
0.3
0.4
0.5
0.6
0.7
0.8
0
200
400
600
800
1000
WT
gnat1-1
gnat1-2
gnat2-1

Y(NO)

**GL**	WT		*gnat1-1*		*gnat1-2*		*gnat2-1*	
PAR (µE)	Average	St. Dev.	Average	St. Dev.	Average	St. Dev.	Average	St. Dev.
50	0.362	0.027	0.363	0.024	0.363	0.028	0.406	0.027
124	0.361	0.024	0.356	0.021	0.355	0.025	0.407	0.023
451	0.287	0.032	0.285	0.032	0.282	0.029	0.311	0.036
962	0.226	0.017	0.224	0.015	0.221	0.015	0.218	0.019

**+ 1 day HL**	WT		*gnat1-1*		*gnat1-2*		*gnat2-1*	
PAR (µE)	Average	St. Dev.	Average	St. Dev.	Average	St. Dev.	Average	St. Dev.
50	0.418	0.034	0.432	0.050	0.449	0.026	0.529	0.058
124	0.392	0.025	0.397	0.030	0.409	0.022	0.522	0.049
451	0.364	0.034	0.368	0.042	0.381	0.022	0.487	0.057
962	0.259	0.037	0.273	0.051	0.269	0.034	0.394	0.064

**+ 2 days HL**	WT		*gnat1-1*		*gnat1-2*		*gnat2-1*	
PAR (µE)	Average	St. Dev.	Average	St. Dev.	Average	St. Dev.	Average	St. Dev.
50	0.406	0.053	0.415	0.048	0.416	0.057	0.451	0.075
124	0.366	0.037	0.379	0.046	0.373	0.046	0.439	0.082
451	0.356	0.059	0.381	0.067	0.368	0.061	0.436	0.082
962	0.282	0.070	0.319	0.084	0.300	0.075	0.391	0.078

**+ 3 days HL**	WT		*gnat1-1*		*gnat1-2*		*gnat2-1*	
PAR (µE)	Average	St. Dev.	Average	St. Dev.	Average	St. Dev.	Average	St. Dev.
50	0.366	0.059	0.391	0.069	0.381	0.066	0.393	0.061
124	0.327	0.045	0.342	0.056	0.335	0.038	0.359	0.071
451	0.350	0.052	0.369	0.057	0.342	0.050	0.349	0.084
962	0.301	0.047	0.331	0.059	0.292	0.055	0.314	0.080

**+ 3 days D**	WT		*gnat1-1*		*gnat1-2*		*gnat2-1*	
PAR (µE)	Average	St. Dev.	Average	St. Dev.	Average	St. Dev.	Average	St. Dev.
50	0.430	0.039	0.426	0.039	0.423	0.034	0.475	0.045
124	0.463	0.046	0.470	0.043	0.449	0.042	0.496	0.047
451	0.436	0.047	0.438	0.038	0.423	0.039	0.455	0.044
962	0.363	0.038	0.369	0.032	0.362	0.037	0.388	0.039

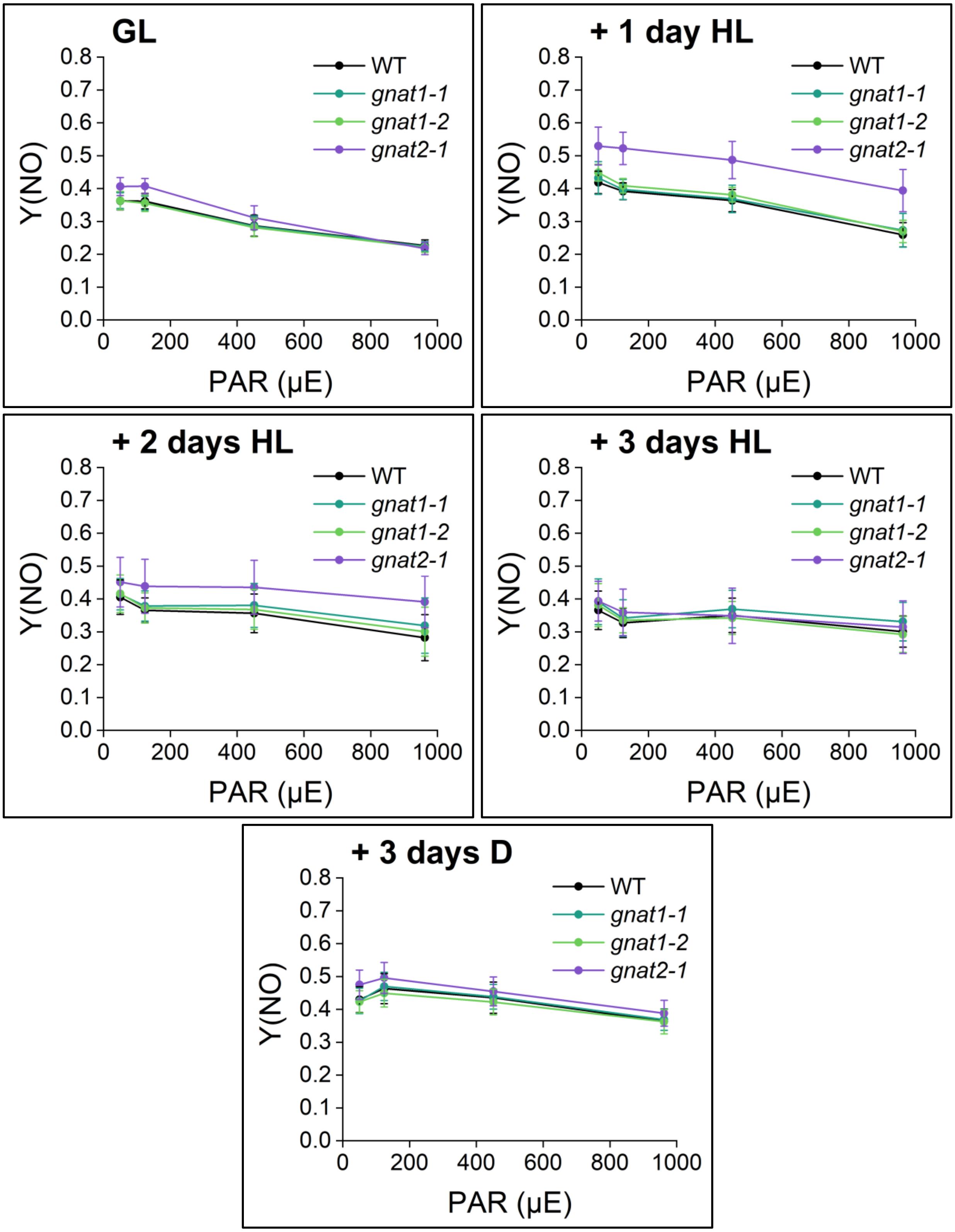

GL
+ 1 day HL
+ 2 days HL
+ 3 days HL
+ 3 days D
WT
gnat1-1
gnat1-2
gnat2-1
Y(NO)
PAR (μE)
0.0
0.1
0.2
0.3
0.4
0.5
0.6
0.7
0.8
0
200
400
600
800
1000

NPQ

GL	WT		*gnat1-1*		*gnat1-2*		*gnat2-1*	
PAR (µE)	Average	St. Dev.	Average	St. Dev.	Average	St. Dev.	Average	St. Dev.
50	0.102	0.026	0.093	0.023	0.095	0.021	0.088	0.015
124	0.135	0.019	0.132	0.017	0.135	0.018	0.118	0.011
451	0.430	0.075	0.436	0.082	0.439	0.084	0.381	0.079
962	0.744	0.068	0.750	0.071	0.769	0.072	0.776	0.093

+ 1 day HL	WT		*gnat1-1*		*gnat1-2*		*gnat2-1*	
PAR (µE)	Average	St. Dev.	Average	St. Dev.	Average	St. Dev.	Average	St. Dev.
50	0.095	0.036	0.083	0.034	0.067	0.013	0.081	0.025
124	0.127	0.022	0.118	0.023	0.109	0.010	0.103	0.024
451	0.272	0.052	0.261	0.060	0.238	0.029	0.190	0.037
962	0.591	0.119	0.561	0.162	0.550	0.115	0.341	0.086

+ 2 days HL	WT		*gnat1-1*		*gnat1-2*		*gnat2-1*	
PAR (µE)	Average	St. Dev.	Average	St. Dev.	Average	St. Dev.	Average	St. Dev.
50	0.110	0.059	0.095	0.045	0.102	0.05	0.208	0.078
124	0.136	0.043	0.118	0.041	0.129	0.039	0.236	0.098
451	0.269	0.076	0.236	0.086	0.251	0.079	0.299	0.103
962	0.544	0.184	0.458	0.200	0.492	0.195	0.390	0.124

+ 3 days HL	WT		*gnat1-1*		*gnat1-2*		*gnat2-1*	
PAR (µE)	Average	St. Dev.	Average	St. Dev.	Average	St. Dev.	Average	St. Dev.
50	0.163	0.09	0.155	0.095	0.168	0.117	0.298	0.109
124	0.168	0.085	0.173	0.103	0.18	0.091	0.371	0.156
451	0.266	0.095	0.245	0.1	0.29	0.106	0.46	0.177
962	0.466	0.131	0.411	0.156	0.508	0.182	0.566	0.198

+ 3 days D	WT		*gnat1-1*		*gnat1-2*		*gnat2-1*	
PAR (µE)	Average	St. Dev.	Average	St. Dev.	Average	St. Dev.	Average	St. Dev.
50	0.106	0.024	0.103	0.03	0.103	0.02	0.084	0.014
124	0.151	0.032	0.147	0.036	0.154	0.032	0.131	0.03
451	0.283	0.049	0.276	0.048	0.288	0.044	0.253	0.04
962	0.413	0.057	0.406	0.057	0.417	0.057	0.369	0.051

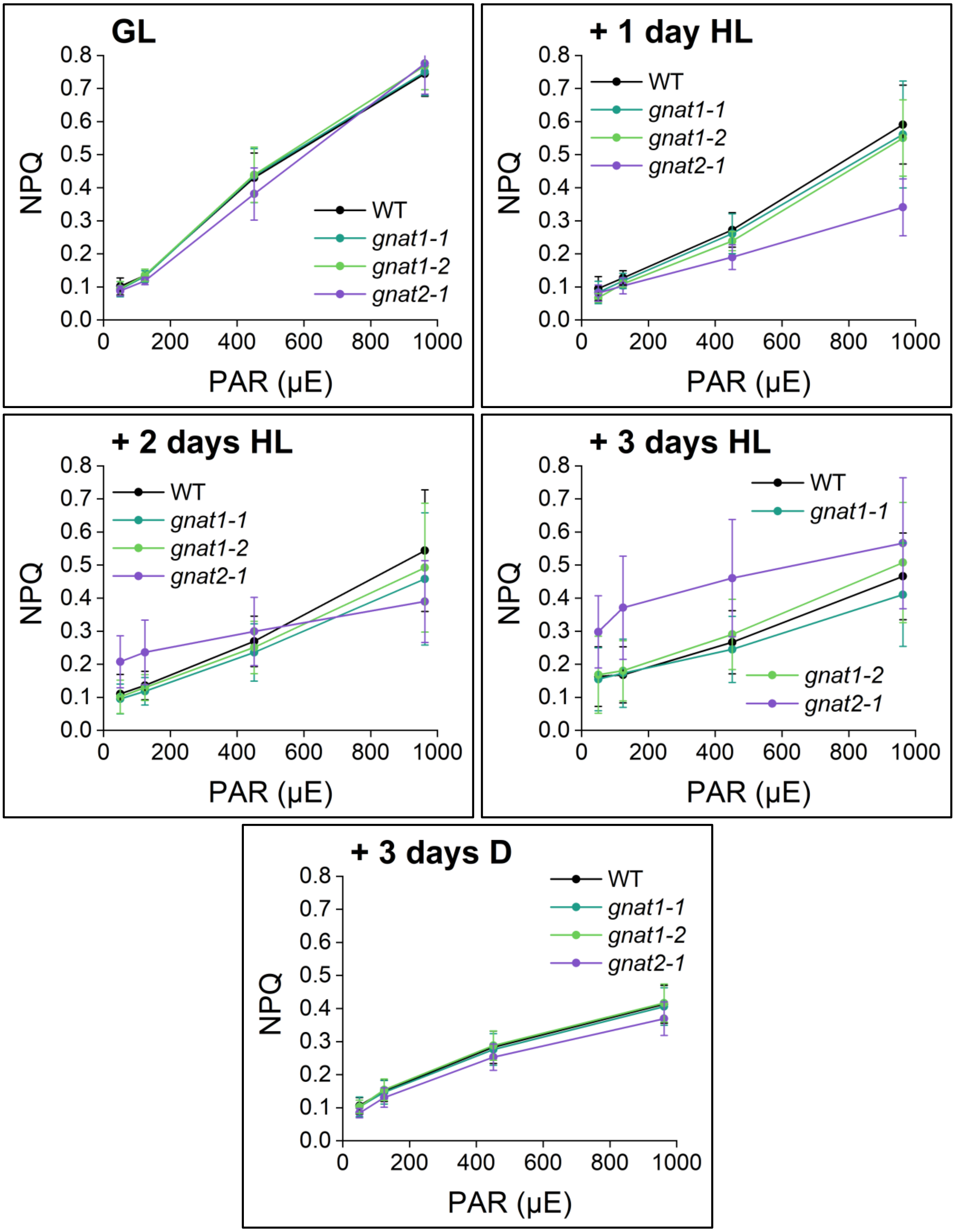

GL
NPQ
0.0
0.1
0.2
0.3
0.4
0.5
0.6
0.7
0.8
0
200
400
600
800
1000
PAR (µE)
WT
gnat1-1
gnat1-2
gnat2-1
+ 1 day HL
+ 2 days HL
+ 3 days HL
+ 3 days D

qN

**GL**	*WT*		*gnat1-1*		*gnat1-2*		*gnat2-1*	
PAR (µE)	Average	St. Dev.	Average	St. Dev.	Average	St. Dev.	Average	St. Dev.
50	0.353	0.058	0.333	0.055	0.338	0.053	0.323	0.041
124	0.430	0.035	0.425	0.033	0.431	0.036	0.399	0.029
451	0.742	0.048	0.745	0.052	0.747	0.046	0.713	0.057
962	0.859	0.019	0.860	0.020	0.864	0.018	0.865	0.025

**+ 1 day HL**	WT		*gnat1-1*		*gnat1-2*		*gnat2-1*	
PAR (µE)	Average	St. Dev.	Average	St. Dev.	Average	St. Dev.	Average	St. Dev.
50	0.347	0.086	0.316	0.092	0.274	0.042	0.364	0.096
124	0.431	0.047	0.412	0.052	0.392	0.024	0.432	0.091
451	0.643	0.052	0.629	0.065	0.609	0.034	0.604	0.073
962	0.827	0.043	0.810	0.062	0.812	0.040	0.757	0.057

**+ 2 days HL**	WT		*gnat1-1*		*gnat1-2*		*gnat2-1*	
PAR (µE)	Average	St. Dev.	Average	St. Dev.	Average	St. Dev.	Average	St. Dev.
50	0.382	0.125	0.350	0.113	0.362	0.121	0.687	0.160
124	0.450	0.086	0.411	0.094	0.435	0.089	0.712	0.163
451	0.639	0.091	0.596	0.107	0.616	0.095	0.779	0.121
962	0.799	0.091	0.753	0.109	0.772	0.099	0.838	0.088

**+ 3 days HL**	WT		*gnat1-1*		*gnat1-2*		*gnat2-1*	
PAR (µE)	Average	St. Dev.	Average	St. Dev.	Average	St. Dev.	Average	St. Dev.
50	0.483	0.158	0.472	0.182	0.473	0.194	0.805	0.150
124	0.501	0.129	0.505	0.163	0.517	0.143	0.835	0.141
451	0.639	0.098	0.619	0.125	0.659	0.108	0.876	0.104
962	0.782	0.073	0.750	0.100	0.791	0.088	0.910	0.073

**+ 3 days D**	WT		*gnat1-1*		*gnat1-2*		*gnat2-1*	
PAR (µE)	Average	St. Dev.	Average	St. Dev.	Average	St. Dev.	Average	St. Dev.
50	0.443	0.059	0.429	0.074	0.425	0.054	0.385	0.038
124	0.543	0.064	0.532	0.068	0.539	0.057	0.505	0.053
451	0.722	0.047	0.713	0.044	0.717	0.038	0.697	0.034
962	0.812	0.028	0.807	0.025	0.806	0.024	0.792	0.023

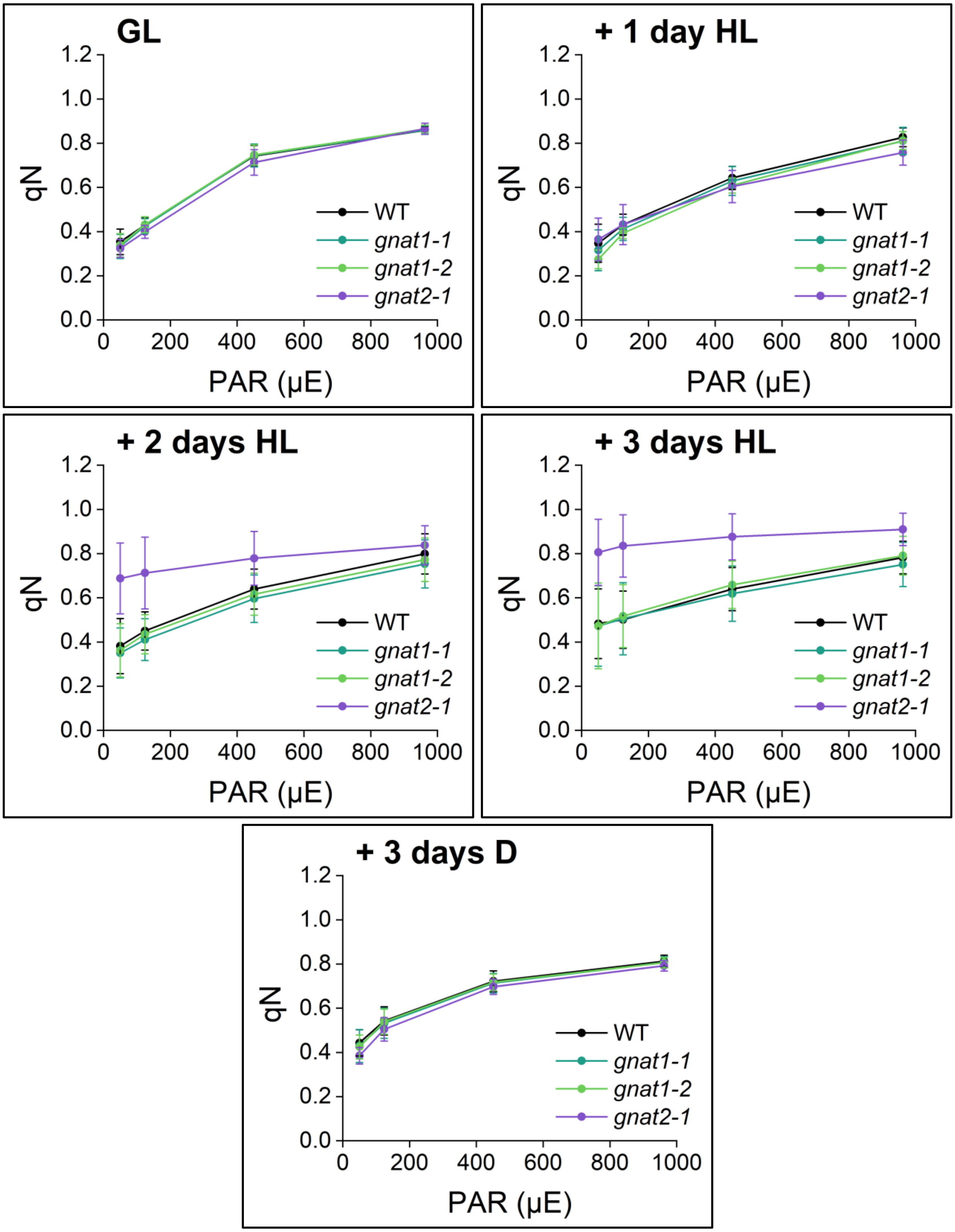
GL
+ 1 day HL
+ 2 days HL
+ 3 days HL
+ 3 days D
qN
PAR (µE)
WT
gnat1-1
gnat1-2
gnat2-1
0.0
0.2
0.4
0.6
0.8
1.0
1.2
0
200
400
600
800
1000

qP

GL	WT		*gnat1-1*		*gnat1-2*		*gnat2-1*	
PAR (µE)	Average	St. Dev.	Average	St. Dev.	Average	St. Dev.	Average	St. Dev.
50	0.678	0.035	0.689	0.035	0.686	0.044	0.621	0.040
124	0.629	0.038	0.645	0.044	0.642	0.049	0.564	0.043
451	0.395	0.037	0.394	0.042	0.402	0.039	0.381	0.027
962	0.213	0.024	0.222	0.029	0.214	0.041	0.240	0.047

+ 1 day HL	WT		*gnat1-1*		*gnat1-2*		*gnat2-1*	
PAR (µE)	Average	St. Dev.	Average	St. Dev.	Average	St. Dev.	Average	St. Dev.
50	0.623	0.035	0.624	0.054	0.614	0.033	0.529	0.075
124	0.617	0.036	0.624	0.044	0.610	0.034	0.476	0.071
451	0.418	0.036	0.433	0.040	0.424	0.028	0.291	0.106
962	0.307	0.033	0.301	0.040	0.313	0.024	0.203	0.082

+ 2 days HL	WT		*gnat1-1*		*gnat1-2*		*gnat2-1*	
PAR (µE)	Average	St. Dev.	Average	St. Dev.	Average	St. Dev.	Average	St. Dev.
50	0.646	0.065	0.652	0.059	0.636	0.077	0.587	0.094
124	0.685	0.055	0.685	0.054	0.675	0.064	0.539	0.112
451	0.483	0.072	0.478	0.067	0.477	0.071	0.196	0.189
962	0.320	0.072	0.312	0.091	0.320	0.098	0.102	0.139

+ 3 days HL	WT		*gnat1-1*		*gnat1-2*		*gnat2-1*	
PAR (µE)	Average	St. Dev.	Average	St. Dev.	Average	St. Dev.	Average	St. Dev.
50	0.668	0.105	0.606	0.155	0.632	0.073	0.637	0.236
124	0.757	0.078	0.711	0.140	0.720	0.050	0.538	0.312
451	0.513	0.122	0.497	0.154	0.511	0.071	0.189	0.235
962	0.324	0.109	0.304	0.133	0.309	0.122	0.111	0.163

+ 3 days D	WT		*gnat1-1*		*gnat1-2*		*gnat2-1*	
PAR (µE)	Average	St. Dev.	Average	St. Dev.	Average	St. Dev.	Average	St. Dev.
50	0.742	0.062	0.751	0.047	0.732	0.063	0.687	0.057
124	0.525	0.047	0.514	0.055	0.535	0.047	0.500	0.057
451	0.173	0.101	0.191	0.056	0.211	0.068	0.211	0.051
962	0.109	0.089	0.094	0.092	0.099	0.087	0.115	0.081

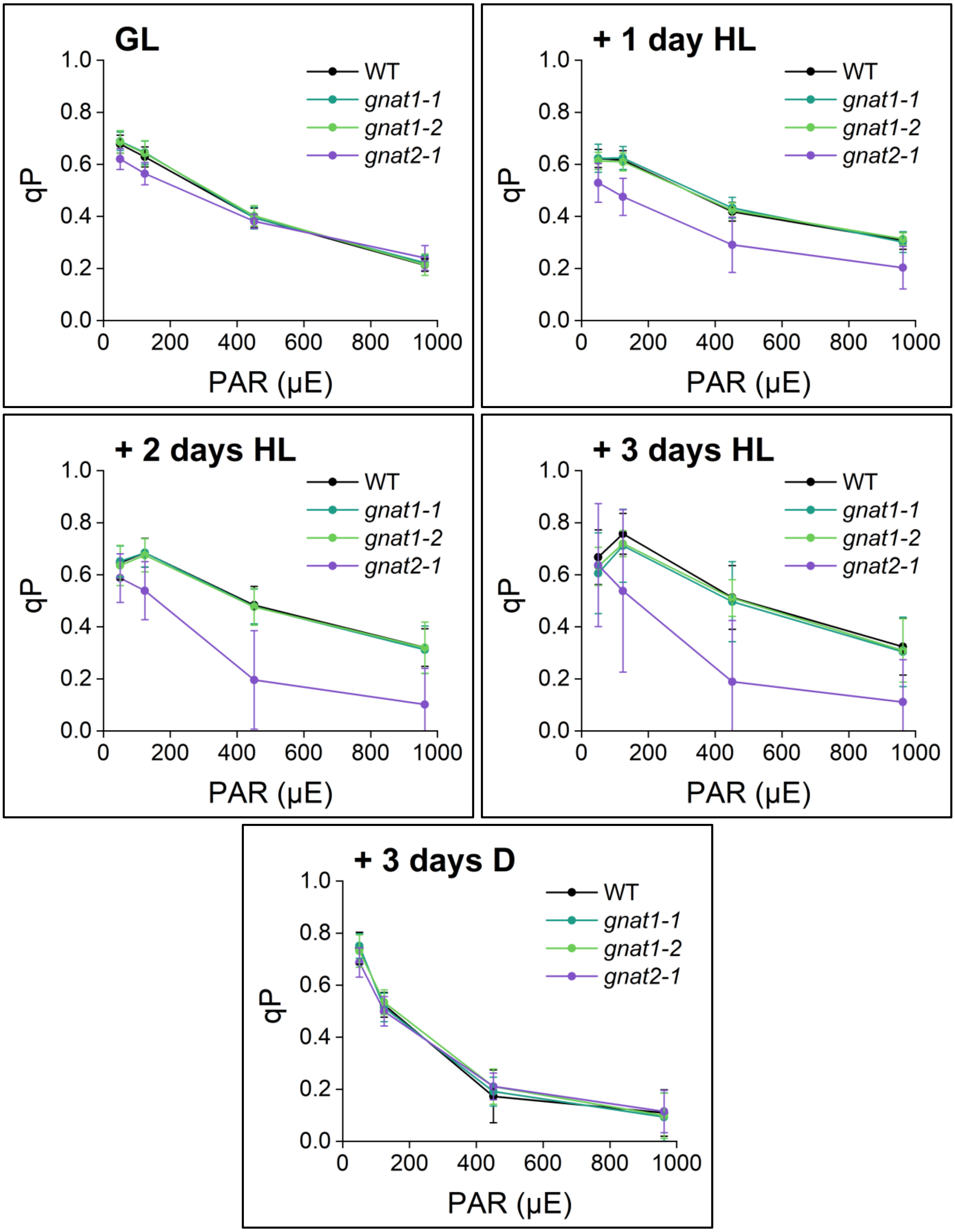

GL
+ 1 day HL
+ 2 days HL
+ 3 days HL
+ 3 days D
qP
PAR (µE)
WT
gnat1-1
gnat1-2
gnat2-1

qL

GL	WT		*gnat1-1*		*gnat1-2*		*gnat2-1*	
PAR (µE)	Average	St. Dev.	Average	St. Dev.	Average	St. Dev.	Average	St. Dev.
50	0.366	0.057	0.374	0.059	0.373	0.066	0.309	0.055
124	0.332	0.055	0.348	0.064	0.347	0.064	0.274	0.054
451	0.216	0.041	0.216	0.044	0.222	0.032	0.201	0.034
962	0.122	0.019	0.128	0.020	0.125	0.026	0.144	0.033

+ 1 day HL	WT		*gnat1-1*		*gnat1-2*		*gnat2-1*	
PAR (µE)	Average	St. Dev.	Average	St. Dev.	Average	St. Dev.	Average	St. Dev.
50	0.342	0.046	0.341	0.074	0.320	0.038	0.329	0.051
124	0.351	0.043	0.356	0.058	0.336	0.036	0.290	0.046
451	0.228	0.032	0.238	0.044	0.224	0.023	0.170	0.063
962	0.189	0.029	0.183	0.039	0.190	0.024	0.127	0.053

+ 2 days HL	WT		*gnat1-1*		*gnat1-2*		*gnat2-1*	
PAR (µE)	Average	St. Dev.	Average	St. Dev.	Average	St. Dev.	Average	St. Dev.
50	0.384	0.100	0.385	0.094	0.370	0.103	0.487	0.113
124	0.436	0.090	0.430	0.081	0.421	0.084	0.443	0.116
451	0.288	0.082	0.275	0.075	0.275	0.075	0.143	0.141
962	0.199	0.066	0.184	0.072	0.191	0.073	0.073	0.102

+ 3 days HL	WT		*gnat1-1*		*gnat1-2*		*gnat2-1*	
PAR (µE)	Average	St. Dev.	Average	St. Dev.	Average	St. Dev.	Average	St. Dev.
50	0.441	0.125	0.389	0.119	0.397	0.102	0.574	0.239
124	0.551	0.121	0.507	0.123	0.499	0.098	0.483	0.293
451	0.320	0.104	0.306	0.116	0.320	0.090	0.151	0.190
962	0.198	0.077	0.179	0.087	0.190	0.087	0.087	0.128

+ 3 days D	WT		*gnat1-1*		*gnat1-2*		*gnat2-1*	
PAR (µE)	Average	St. Dev.	Average	St. Dev.	Average	St. Dev.	Average	St. Dev.
50	0.578	0.088	0.584	0.064	0.552	0.083	0.508	0.066
124	0.356	0.045	0.345	0.061	0.357	0.050	0.334	0.061
451	0.105	0.060	0.118	0.037	0.128	0.040	0.132	0.036
962	0.071	0.058	0.059	0.059	0.062	0.054	0.074	0.053

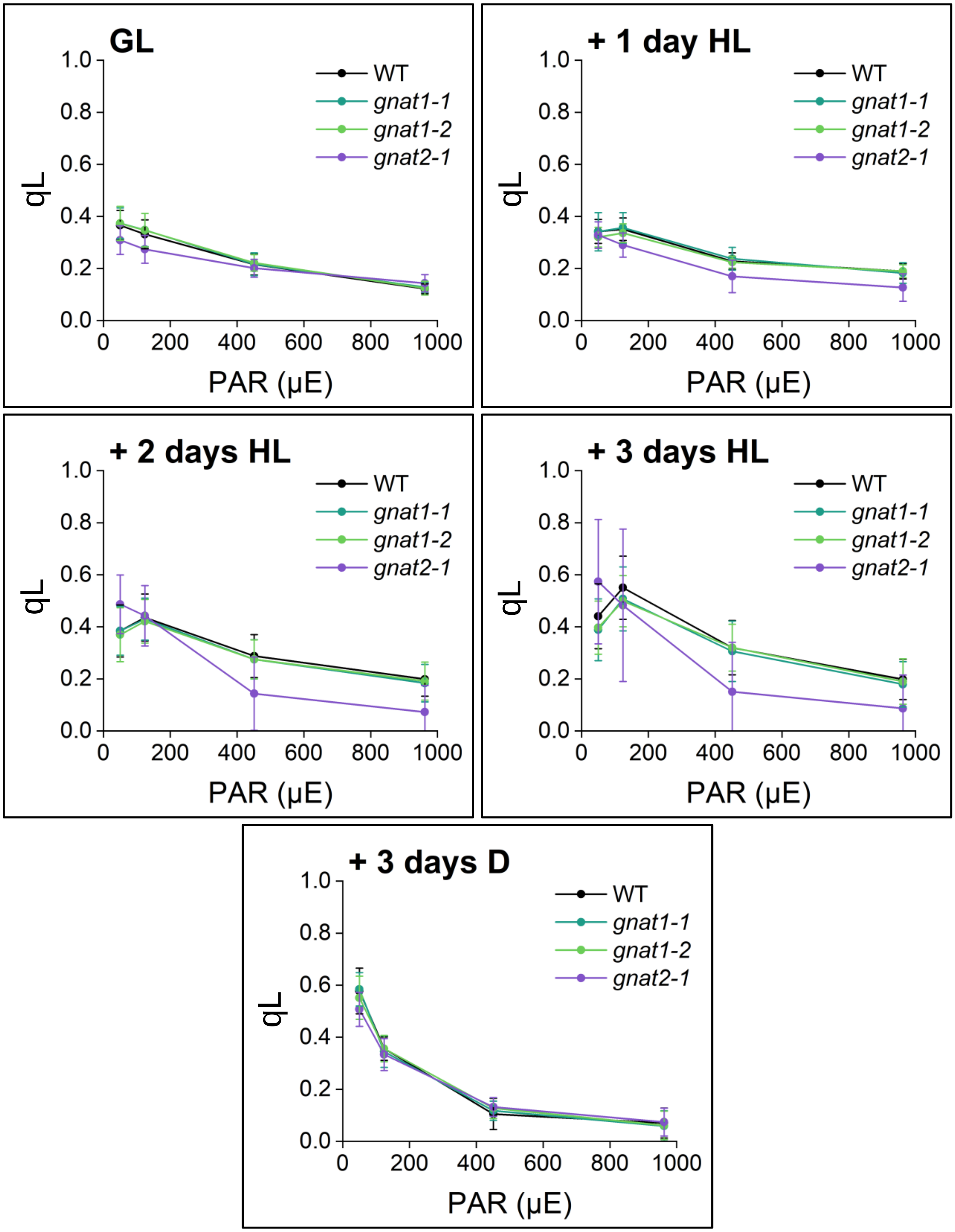

GL
+ 1 day HL
+ 2 days HL
+ 3 days HL
+ 3 days D
qL
PAR (µE)
WT
gnat1-1
gnat1-2
gnat2-1
0.0
0.2
0.4
0.6
0.8
1.0
0
200
400
600
800
1000

Fm, Fm’ (normalized to WT (Fm), PAR = 0 µE, respectively)

**GL**	WT		*gnat1-1*		*gnat1-2*		*gnat2-1*	
PAR (µE)	Average	St. Dev.	Average	St. Dev.	Average	St. Dev.	Average	St. Dev.
50	0.999	0.122	1.017	0.139	0.959	0.126	1.025	0.116
124	0.713	0.096	0.743	0.107	0.696	0.082	0.759	0.083
451	0.650	0.079	0.666	0.090	0.622	0.075	0.695	0.072
962	0.370	0.051	0.376	0.068	0.352	0.053	0.411	0.061

**+ 1 day HL**	WT		*gnat1-1*		*gnat1-2*		*gnat2-1*	
PAR (µE)	Average	St. Dev.	Average	St. Dev.	Average	St. Dev.	Average	St. Dev.
50	0.999	0.13	0.913	0.083	0.918	0.124	0.753	0.137
124	0.734	0.132	0.693	0.101	0.725	0.105	0.575	0.126
451	0.663	0.090	0.622	0.074	0.638	0.088	0.537	0.116
962	0.480	0.059	0.453	0.071	0.471	0.063	0.432	0.090

**+ 2 days HL**	WT		*gnat1-1*		*gnat1-2*		*gnat2-1*	
PAR (µE)	Average	St. Dev.	Average	St. Dev.	Average	St. Dev.	Average	St. Dev.
50	0.999	0.147	0.933	0.162	0.960	0.159	0.778	0.123
124	0.713	0.162	0.684	0.136	0.699	0.158	0.444	0.136
451	0.655	0.120	0.637	0.109	0.639	0.116	0.424	0.140
962	0.491	0.099	0.487	0.080	0.488	0.095	0.373	0.120

**+ 3 days HL**	WT		*gnat1-1*		*gnat1-2*		*gnat2-1*	
PAR (µE)	Average	St. Dev.	Average	St. Dev.	Average	St. Dev.	Average	St. Dev.
50	1.001	0.164	0.851	0.132	1.024	0.143	0.886	0.161
124	0.638	0.188	0.550	0.142	0.662	0.207	0.420	0.114
451	0.620	0.155	0.526	0.126	0.624	0.164	0.379	0.118
962	0.500	0.124	0.444	0.100	0.494	0.124	0.331	0.103

**+ 3 days D**	WT		*gnat1-1*		*gnat1-2*		*gnat2-1*	
PAR (µE)	Average	St. Dev.	Average	St. Dev.	Average	St. Dev.	Average	St. Dev.
50	1.000	0.116	0.980	0.162	0.983	0.152	0.963	0.171
124	0.703	0.079	0.692	0.085	0.696	0.097	0.721	0.126
451	0.627	0.085	0.619	0.101	0.610	0.097	0.633	0.105
962	0.472	0.068	0.467	0.074	0.458	0.071	0.480	0.080

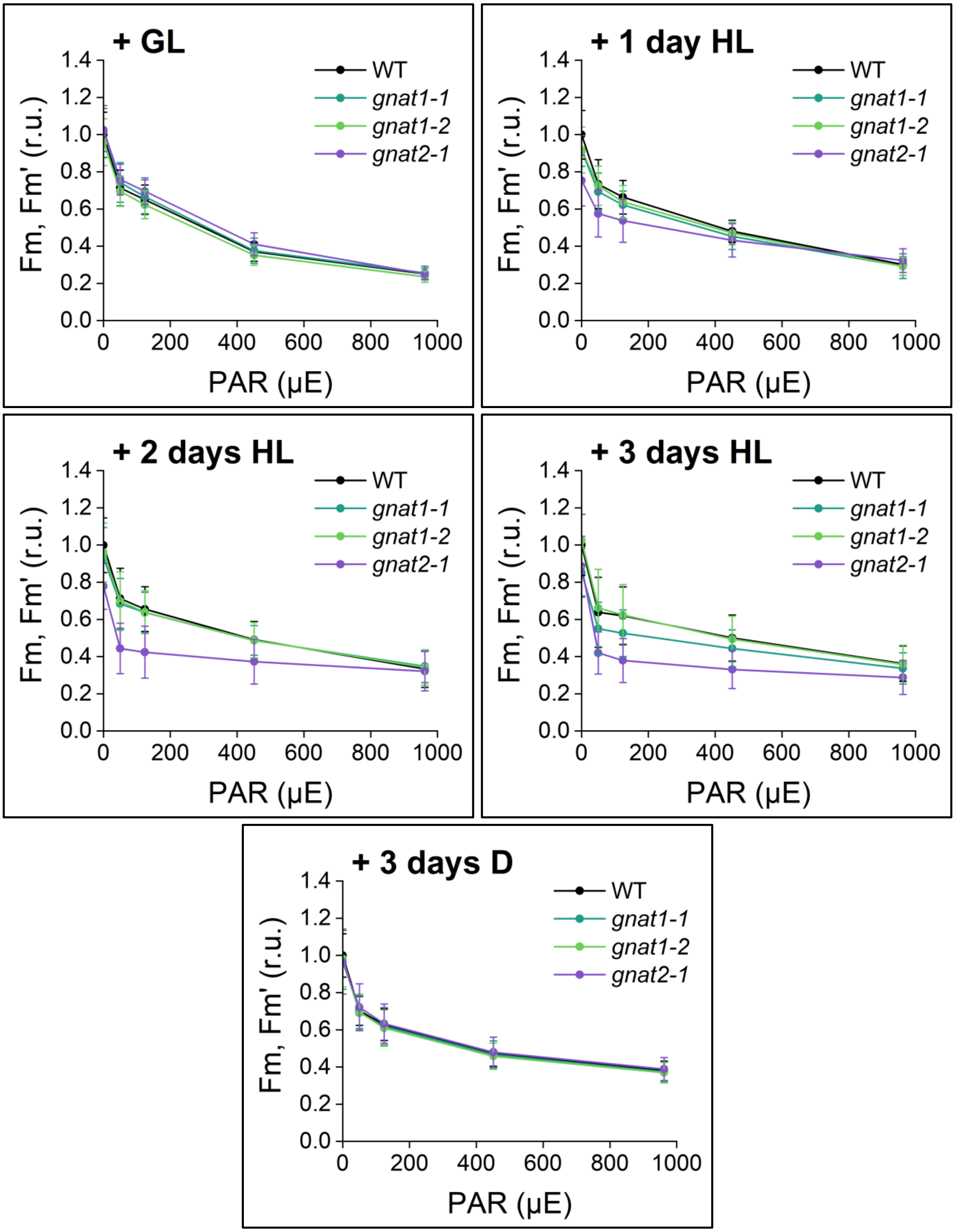
+ GL
+ 1 day HL
+ 2 days HL
+ 3 days HL
+ 3 days D
Fm, Fm' (r.u.)
PAR (μE)
WT
gnat1-1
gnat1-2
gnat2-1
1.4
1.2
1.0
0.8
0.6
0.4
0.2
0.0
0
200
400
600
800
1000

F_0, F_0’ (normalized to WT (F_0), PAR = 0 µE, respectively)

**GL**	WT		*gnat1-1*		*gnat1-2*		*gnat2-1*	
PAR (µE)	Average	St. Dev.	Average	St. Dev.	Average	St. Dev.	Average	St. Dev.
50	1.004	0.188	1.017	0.156	0.966	0.172	1.055	0.188
124	0.925	0.171	0.944	0.143	0.893	0.15	0.979	0.168
451	0.901	0.162	0.915	0.135	0.866	0.145	0.955	0.159
962	0.734	0.116	0.743	0.105	0.703	0.111	0.790	0.119

**+ 1 day HL**	WT		*gnat1-1*		*gnat1-2*		*gnat2-1*	
PAR (µE)	Average	St. Dev.	Average	St. Dev.	Average	St. Dev.	Average	St. Dev.
50	1.003	0.129	0.920	0.082	0.918	0.135	1.104	0.163
124	0.917	0.125	0.849	0.078	0.860	0.126	0.983	0.136
451	0.889	0.112	0.822	0.074	0.828	0.120	0.954	0.132
962	0.788	0.090	0.729	0.070	0.742	0.103	0.861	0.124

**+ 2 days HL**	WT		*gnat1-1*		*gnat1-2*		*gnat2-1*	
PAR (µE)	Average	St. Dev.	Average	St. Dev.	Average	St. Dev.	Average	St. Dev.
50	1.003	0.188	0.943	0.205	0.954	0.182	1.584	0.352
124	0.895	0.165	0.852	0.167	0.859	0.161	1.092	0.238
451	0.872	0.148	0.832	0.157	0.835	0.142	1.051	0.242
962	0.775	0.117	0.747	0.115	0.750	0.116	0.972	0.24

**+ 3 days HL**	WT		*gnat1-1*		*gnat1-2*		*gnat2-1*	
PAR (µE)	Average	St. Dev.	Average	St. Dev.	Average	St. Dev.	Average	St. Dev.
50	1.004	0.231	0.912	0.266	1.012	0.160	1.956	0.576
124	0.839	0.159	0.751	0.156	0.843	0.110	1.106	0.244
451	0.832	0.144	0.737	0.155	0.833	0.102	1.010	0.241
962	0.762	0.132	0.688	0.134	0.756	0.086	0.916	0.236

**+ 3 days D**	WT		*gnat1-1*		*gnat1-2*		*gnat2-1*	
PAR (µE)	Average	St. Dev.	Average	St. Dev.	Average	St. Dev.	Average	St. Dev.
50	1.000	0.163	0.963	0.177	0.92	0.138	0.972	0.142
124	0.858	0.128	0.829	0.126	0.799	0.105	0.860	0.128
451	0.811	0.129	0.786	0.139	0.752	0.112	0.808	0.117
962	0.696	0.110	0.677	0.114	0.649	0.093	0.697	0.102

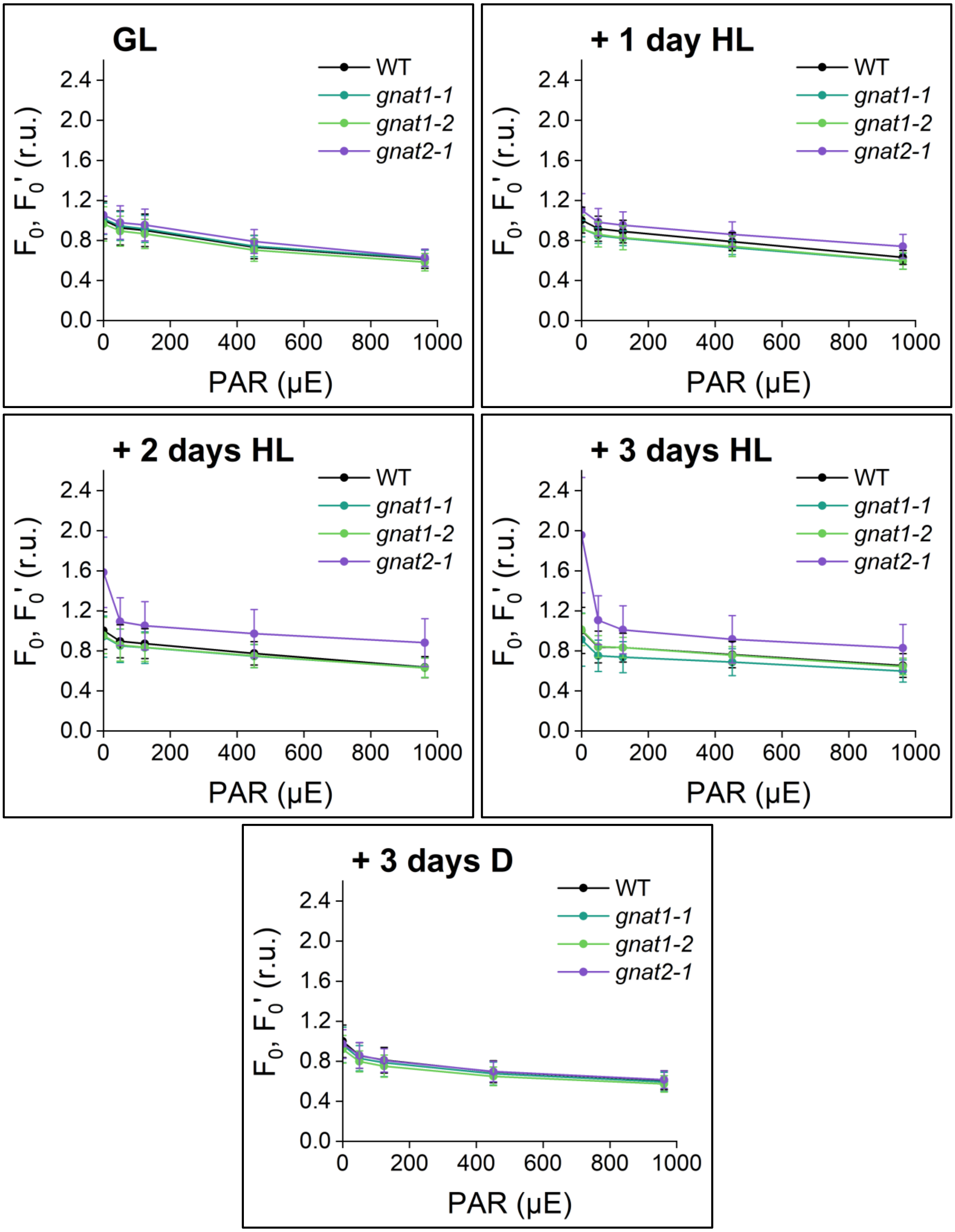
GL
+ 1 day HL
+ 2 days HL
+ 3 days HL
+ 3 days D
WT
gnat1-1
gnat1-2
gnat2-1
F0, F0' (r.u.)
PAR (μE)
0.0
0.4
0.8
1.2
1.6
2.0
2.4
0
200
400
600
800
1000
