# Supplementary material for: The Plastidial Protein Acetyltransferase GNAT1 Forms a Complex With GNAT2, yet Their Interaction Is Dispensable for State Transitions
Source: Mol Cell Proteomics. 2024 Sep 28;23(11):100850. doi: 10.1016/j.mcpro.2024.100850 (PMC11585782; doi:10.1016/j.mcpro.2024.100850)
Supplement: Suppl. Fig. 1 [file mmc11.pdf]

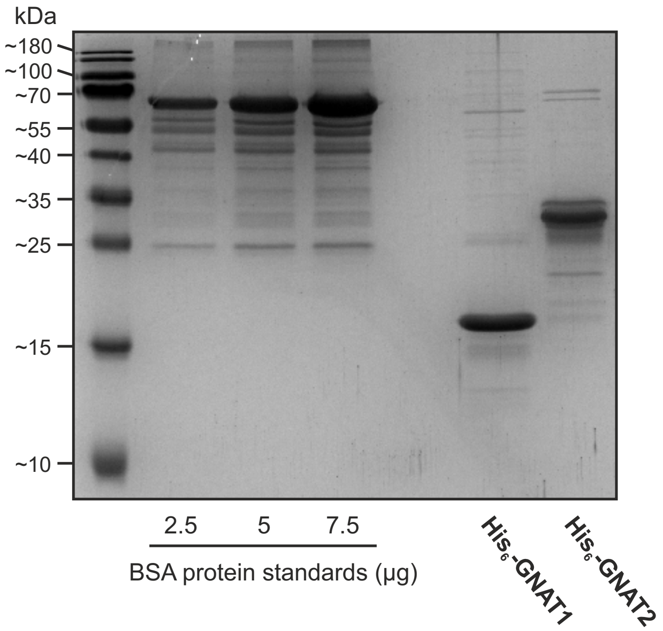

Supplemental Figure 1. Preparations of recombinant His$_6$-GNAT1 and His$_6$-GNAT2 separated by SDS gelelectrophoresis. Both acetyltransferases were heterologously expressed in *E. coli* and purified from the soluble phase by Ni-NTA affinity chromatography. Per preparation, 5 µg were loaded on a 15% [w/v] acrylamide-bisacrylamide SDS gel and separated by electrophoresis. Samples of a BSA protein standard were used as a reference.
