# Supplementary material for: The Plastidial Protein Acetyltransferase GNAT1 Forms a Complex With GNAT2, yet Their Interaction Is Dispensable for State Transitions
Source: Mol Cell Proteomics. 2024 Sep 28;23(11):100850. doi: 10.1016/j.mcpro.2024.100850 (PMC11585782; doi:10.1016/j.mcpro.2024.100850)
Supplement: Suppl. Fig. 2 [file mmc12.pdf]

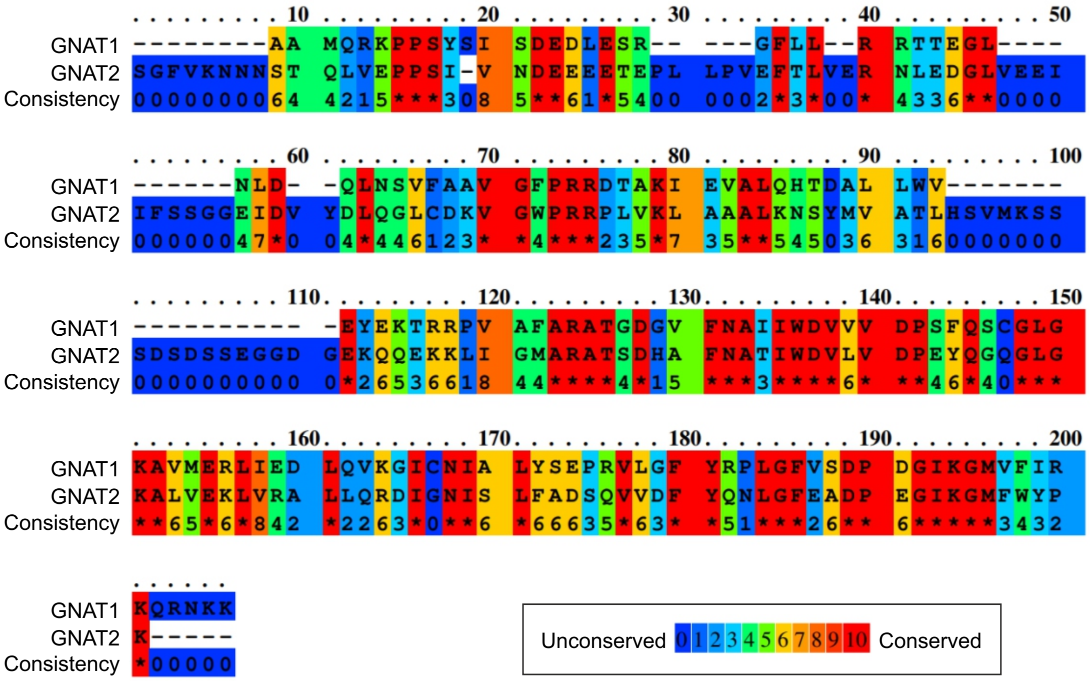

Supplemental Figure 2. Protein sequence alignment of GNAT1 and GNAT2. The full sequences trimmed by the predicted transit-peptide coding regions, respectively, were analyzed by using the PRALINE Multiple Sequence Alignment tool (44, 45). Each position of the alignment is highlighted by the shade of a color code, which represents the level of amino acid conservation and ranges from 0 (dark blue, least conserved) to 10 (dark red, most conserved). The details of the alignment results are as follows: alignment score, 1904.00; alignment score per aligned residue pair, 12.28; sequence identities, 64; sequence identity, 0.41; number of sequences, 2; alignment length, 206; number of residues, 361; number of gaps, 51.
