# Supplementary material for: The Plastidial Protein Acetyltransferase GNAT1 Forms a Complex With GNAT2, yet Their Interaction Is Dispensable for State Transitions
Source: Mol Cell Proteomics. 2024 Sep 28;23(11):100850. doi: 10.1016/j.mcpro.2024.100850 (PMC11585782; doi:10.1016/j.mcpro.2024.100850)
Supplement: Suppl. Fig. 3 [file mmc13.pdf]

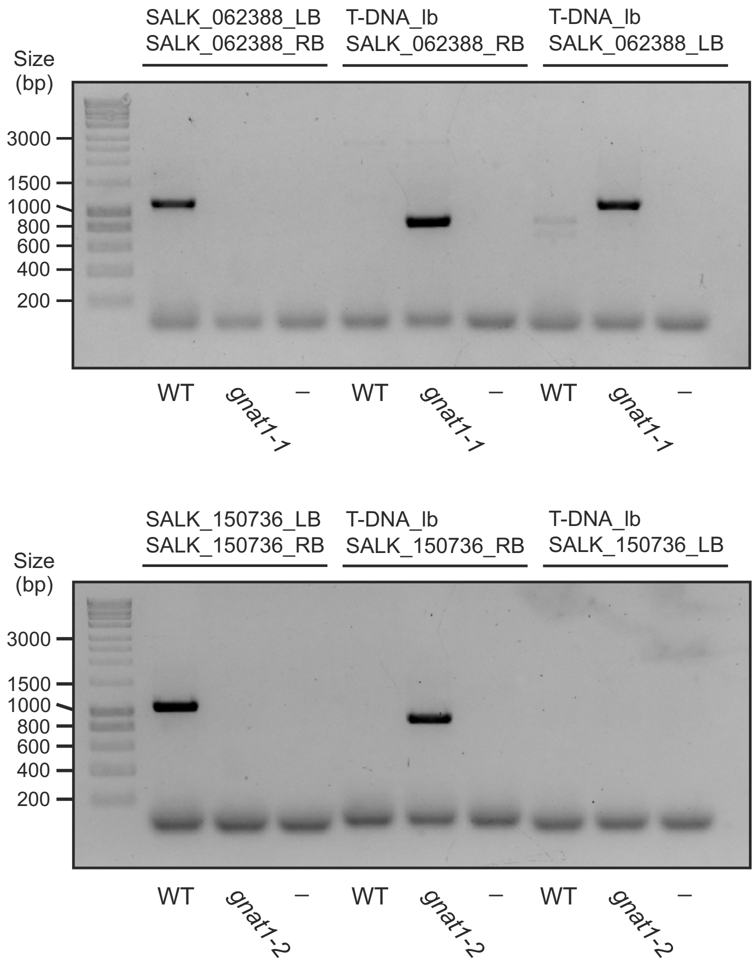

Supplemental Figure 3. Confirmation of genomic T-DNA insertion for the Arabidopsis mutant lines *gnat1-1* (SALK_062388) and *gnat1-2* (SALK_150736) via PCR analysis. Gene-specific LB and RB primer oligonucleotides were used to amplify a DNA fragment from an intact gene locus, whereas combinations of the T-DNA-specific primer T-DNA_lb and the primers LB or RB were applied to verify the integration of T-DNA as well as its orientation. Genomic DNA of wild type plants (WT) and PCR reactions without any DNA (-) were used as control. By PCR and subsequent sequencing of the PCR product, *gnat1-2* was confirmed to carry one T-DNA element inserted at the designated site, while *gnat1-1* was found to harbor two T-DNA elements integrated side-by-side at the designated locus.
