# Supplementary material for: The Plastidial Protein Acetyltransferase GNAT1 Forms a Complex With GNAT2, yet Their Interaction Is Dispensable for State Transitions
Source: Mol Cell Proteomics. 2024 Sep 28;23(11):100850. doi: 10.1016/j.mcpro.2024.100850 (PMC11585782; doi:10.1016/j.mcpro.2024.100850)
Supplement: Suppl. Fig. 4 [file mmc14.pdf]

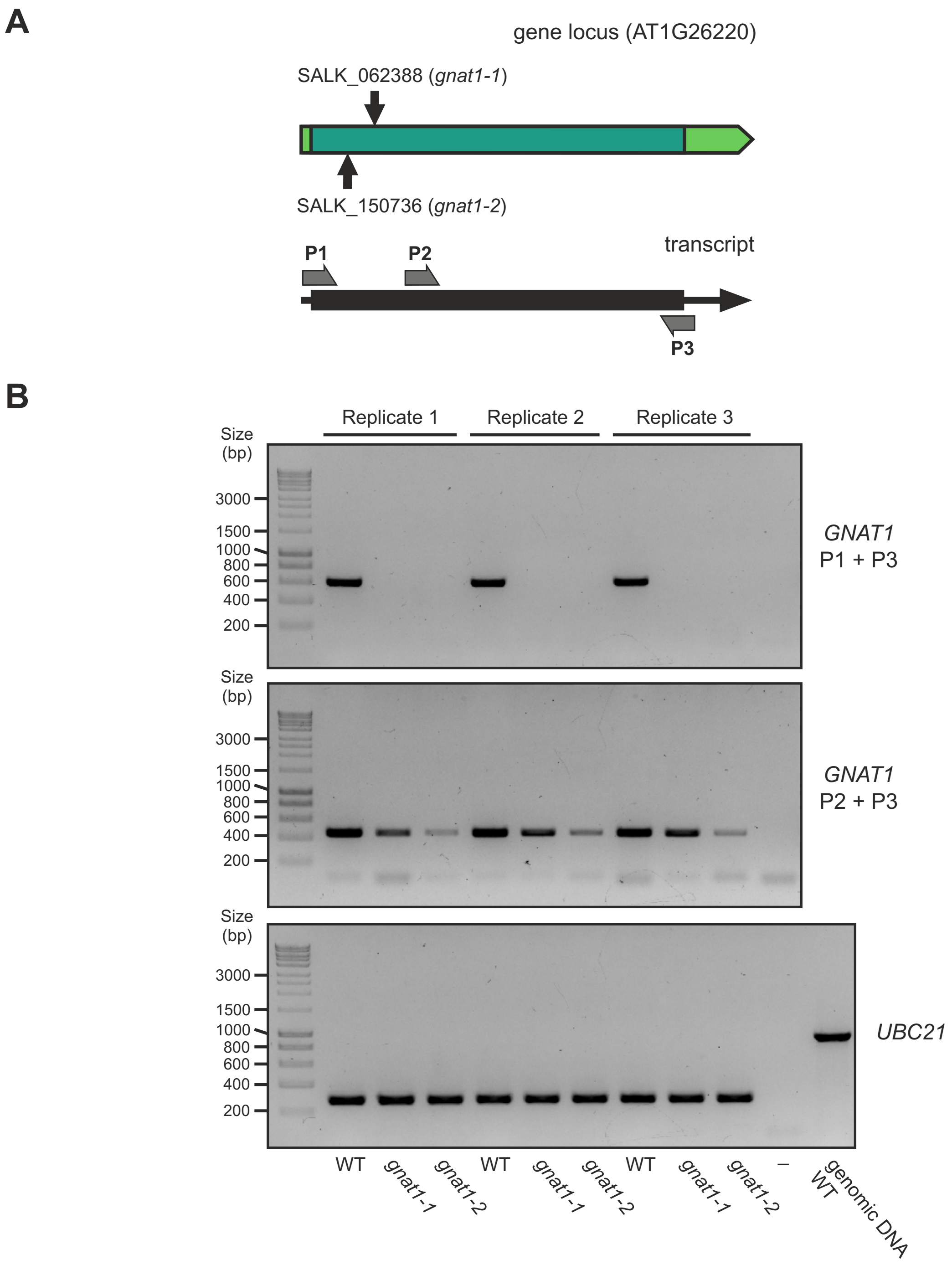

Supplemental Figure 4. Analysis of *GNAT1* transcript abundance in the Arabidopsis mutant lines *gnat1-1* and *gnat1-2* by end-point RT-PCR. (A) Gene and transcript models of GNAT1 based on the annotation by Araport11 (72). The protein coding region is marked by dark green color (gene) or a bold black line (transcript), whereas light green boxes or thin black lines represent untranslated regions (5'- and 3'-UTR). As illustrated, the gene locus AT1G26220 does not contain any introns. Black arrows indicate the sites of T-DNA insertion in the two knockout lines *gnat1-1* (SALK_062388) and *gnat1-2* (SALK_150736) as confirmed by PCR and sequencing of PCR products (Suppl. Fig. 3). Grey tapered-shape boxes display the binding regions that correspond to the primers P1, P2, and P3. The primer pair P1 and P3 enables the amplification of a DNA fragment containing the full GNAT1 coding sequence, while the combination of P2 and P3 results in a shorter amplicon covering the region downstream of the genomic T-DNA insertion sites in *gnat1-1* and *gnat1-2* **(B)**. For both lines, residual *GNAT1* mRNA could be detected as shown by the amplification of the P2 and P3 specific PCR product. The quality of the RNA and/or cDNA preparations was tested by amplifying a fragment of the *UBC21* transcript. Since the genomic DNA of the gnat1 locus does not contain any intron element, the *UBC21* primers were designed in such a way that amplification of genomic DNA would generate a fragment of larger size comprising several intragenic regions of ubc21.
