# Supplementary material for: The Plastidial Protein Acetyltransferase GNAT1 Forms a Complex With GNAT2, yet Their Interaction Is Dispensable for State Transitions
Source: Mol Cell Proteomics. 2024 Sep 28;23(11):100850. doi: 10.1016/j.mcpro.2024.100850 (PMC11585782; doi:10.1016/j.mcpro.2024.100850)
Supplement: Suppl. Fig. 5 [file mmc15.pdf]

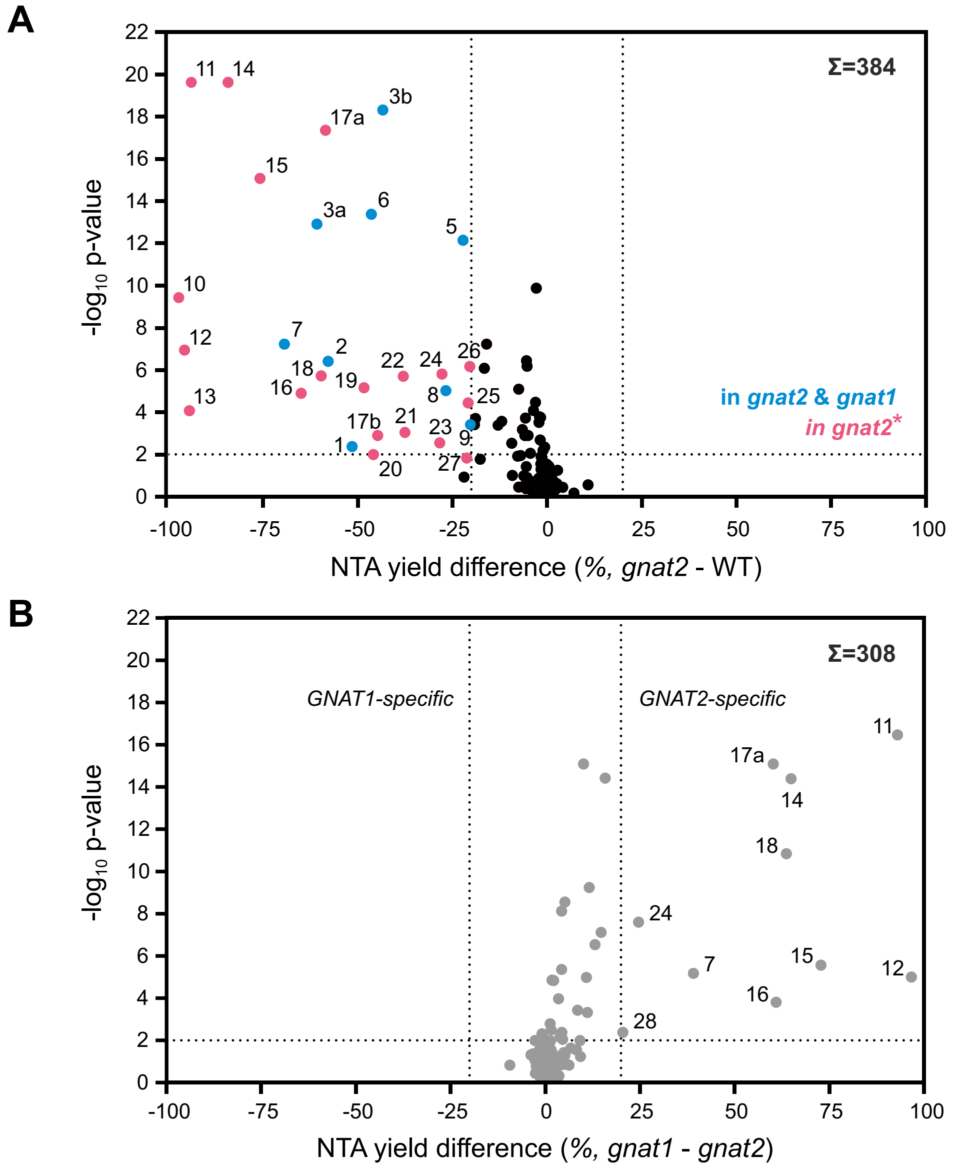

Supplemental Figure 5. NTA yield differences determined for plants of the *gnat2* background and offset of NTA yield profiles defined for GNAT1 and/or GNAT2 deficient plants. (A) comprises the differential NTA yield profile determined for *gnat2* mutant plants that was investigated by Bienvenut and coworkers and by Eirich and coworkers (26, 84). For the wild type (WT) profile, data obtained in this work and the previously published data sets were combined. Significantly up- or downregulated N-termini were marked by red or blue color according to their additional identification in the *gnat1* N-terminal acetylome. (*) The term ‚*in gnat2*' refers to NTA sites that were identified in *gnat2* and were either not detected in *gnat1*, not regarded as significant, or did not fulfill the criteria for quantification. The NTA differences between *gnat2* and wild type were analyzed with GraphPad 10. An unpaired t-test analysis with FDR 1% was performed and N-termini displaying >20% difference in their NTA yield were recognized as significant. An offsetting of NTA yields attributed to GNAT1 and GNAT2 is presented in **(B)**. The profile of NTA yield differences defined for *gnat1* mutant plants is shown in Fig. 3 D.

1 THIC (T55, AT2G29630)
2 SYNO (T60, AT4G17300)
3a F16P1 (V61, AT3G54050)
3b F16P1 (A60, AT3G54050)
4 ZHD10 (A72, AT2G21530)
5 ZEP (A61, AT5G67030)
6 PORC (T69, AT1G03630)
7 NAGK (T51, AT3G57560)
8 AT2G14880 (T46)
9 MORF9 (T61, AT1G11430)
10 CYSK4 (A57, AT3G03630)
11 TIC55 (A51, AT2G24820)
12 HC244 (S65, AT4G35250)
13 ATPB (R2, ATCG00480)
14 PORB (T68, AT4G27440)
15 PSNB2 (S19, AT1G64770)
16 LHCA5 (A33, AT1G45474)
17a AT1G16080 (A45)
17b AT1G16080 (A44)
18 ASSY (V75, AT4G24830)
19 BQMT (S51, AT3G63410)
20 FK172 (A29, AT1G18170)
21 IDI1 (S55, AT5G16440)
22 CLPP4 (S66, AT5G45390)
23 EXEC1 (S48, AT4G33630)
24 LPA3 (G25, AT1G73060)
25 FNRL2 (Q57, AT1G20020)
26 RK15 (T68, AT3G25920)
27 AT4G34290 (A50)
28 AT4G36530 (S53)
