# Supplementary material for: The Plastidial Protein Acetyltransferase GNAT1 Forms a Complex With GNAT2, yet Their Interaction Is Dispensable for State Transitions
Source: Mol Cell Proteomics. 2024 Sep 28;23(11):100850. doi: 10.1016/j.mcpro.2024.100850 (PMC11585782; doi:10.1016/j.mcpro.2024.100850)
Supplement: Suppl. Fig. 6 [file mmc16.pdf]

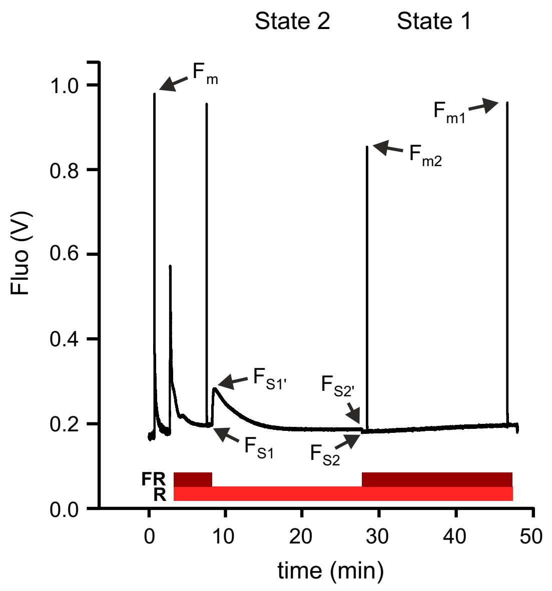

Supplemental Figure 6. Principle of state transition analysis by qT determination. The representative graph shows the chlorophyll *a* fluorescence signal, which was recorded during the experiment by using a Dual-PAM pulse amplitude modulation fluorometer (Walz). Here, the leaf of a wild type plant was subjected to different light conditions followed by the application of a saturating pulse, respectively. After an initial dark incubation period of 20 min, F_0- and F_m- parameters were determined, followed by a 5 min light phase of red and far red light to activate photosynthesis. State 2 conditions were obtained by applying red light only, whereas state 1 conditions comprised the combination of red and far red light (60).
