# Supplementary material for: The Plastidial Protein Acetyltransferase GNAT1 Forms a Complex With GNAT2, yet Their Interaction Is Dispensable for State Transitions
Source: Mol Cell Proteomics. 2024 Sep 28;23(11):100850. doi: 10.1016/j.mcpro.2024.100850 (PMC11585782; doi:10.1016/j.mcpro.2024.100850)
Supplement: Suppl. Fig. 7 [file mmc17.pdf]

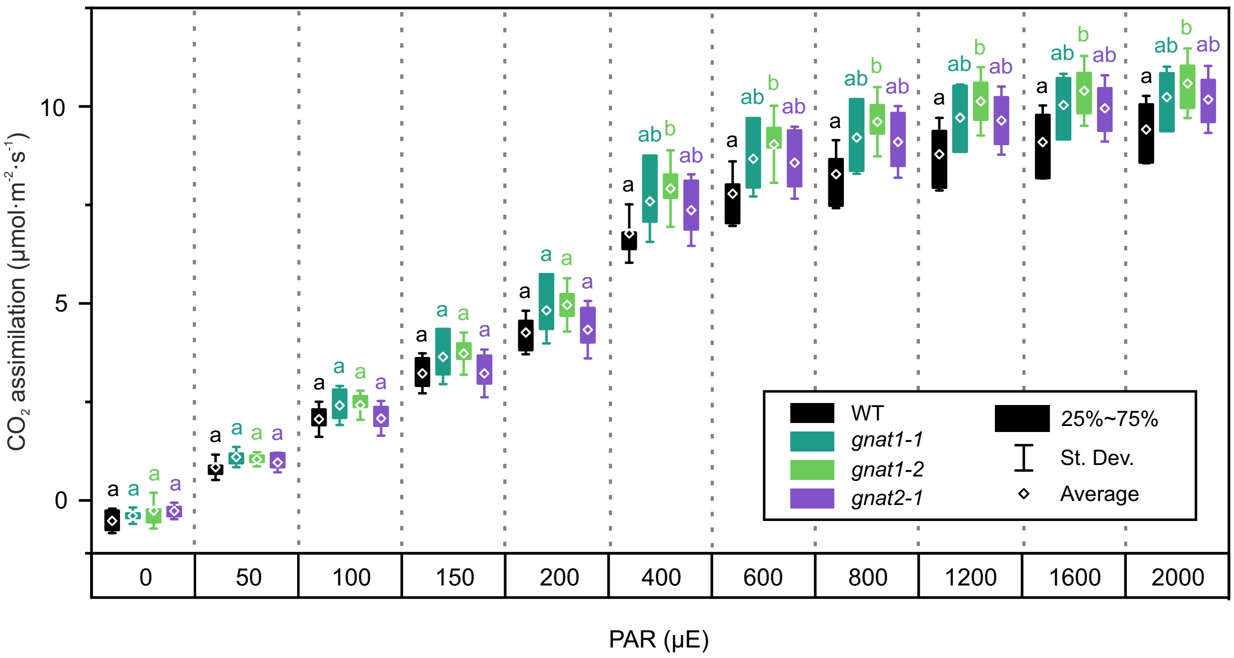

Supplemental Figure 7. Rates of CO_2 assimilation measured for *gnat1-1*, *gnat1-2*, *gnat2-1* and wild type plants (WT). Six weeks old plants adapted to GL (PPFD = 100 µmol · m^{-2} · s^{-1}) were placed into the measuring chamber of the LI-6800 (LI-COR) gas exchange system and incubated in darkness and with a constant CO_2 concentration of 400 ppm. As soon as a stable CO_2 level was established, the actual assimilation rate was recorded and averaged over a time period of 20-30 sec. In a stepwise approach, the light intensity was increased and the CO_2 assimilation rate determined in a similar manner. The leaf area of each plant was calculated by dissecting the corresponding rosette and determining its overall surface with the help of an image analysis software (ImageJ). For further normalization, each plant pot containing only the leftover soil was measured in the same experimental setup for its background level of fluctuation in CO_2 concentration. Six plants per genotype were analyzed and differences between the plant lines tested for significance by a One-Way ANOVA approach and a Fisher LSD test with a significance level of $p \leq 0.05$.
