# Supplementary material for: The Plastidial Protein Acetyltransferase GNAT1 Forms a Complex With GNAT2, yet Their Interaction Is Dispensable for State Transitions
Source: Mol Cell Proteomics. 2024 Sep 28;23(11):100850. doi: 10.1016/j.mcpro.2024.100850 (PMC11585782; doi:10.1016/j.mcpro.2024.100850)
Supplement: Suppl. Fig. 8 [file mmc18.pdf]

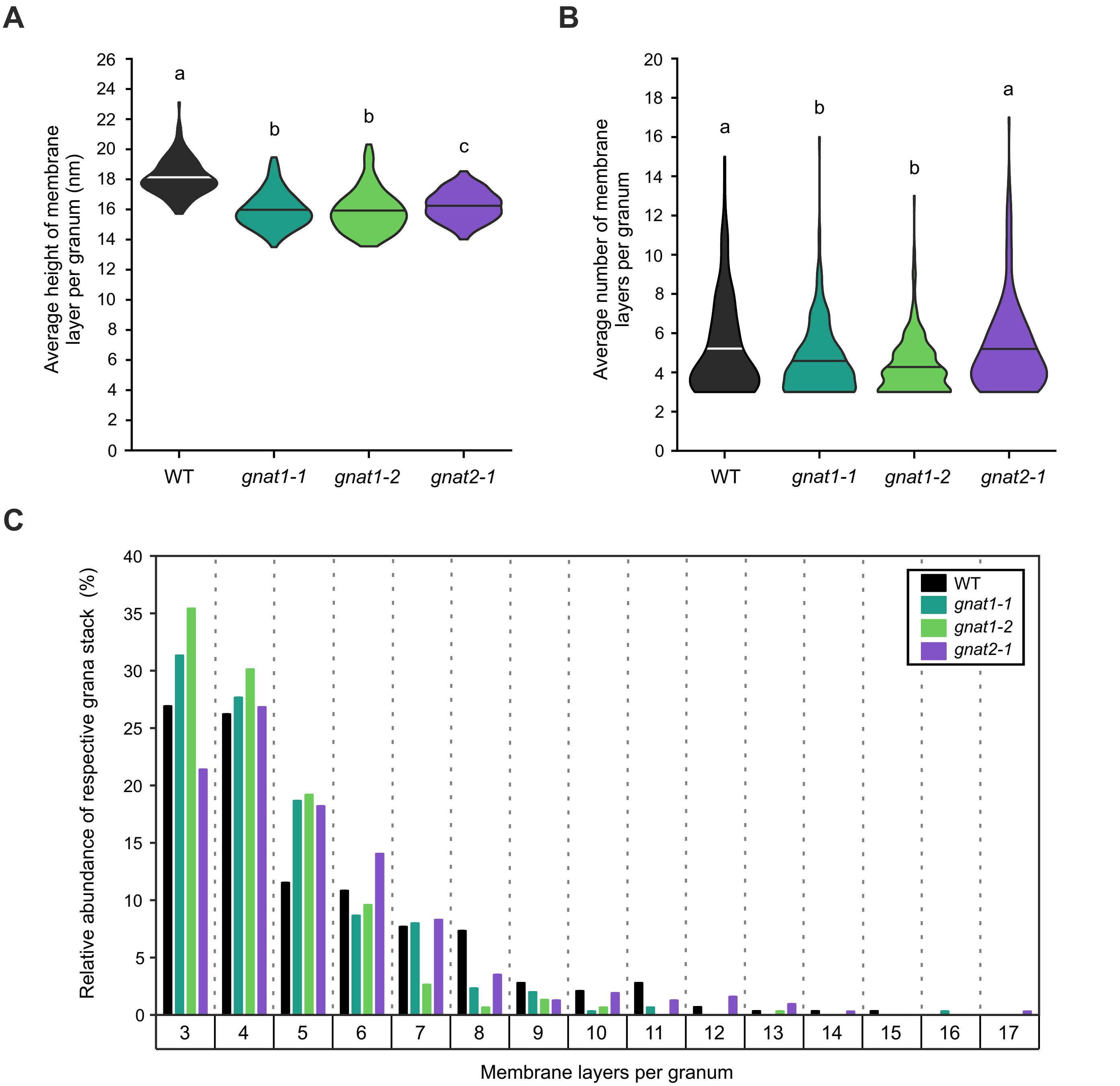

Supplemental Figure 8. Ultrastructural organization of thylakoid membranes in wild type, *gnat1-1*, *gnat1-2* and *gnat2-1* chloroplasts revealed by Transmission Electron Microscopy (TEM). (A) Average membrane layer heights per granum measured for both *gnat1* lines in comparison to wild type and the *gnat2-1* knockout mutant. Per genotype, 160 grana stacks displayed in 16 TEM pictures of at least five biological replicates were analyzed and represented as violin plot illustrating the distribution of data points and showing average value as continuous lines. Differences between genotypes were tested for significance by a One-Way ANOVA approach applying a Fisher LSD test with a significance level of $p \leq 0.05$. The average heights of membrane layers per granum are as follows (average ± standard deviation): WT, 18.14 ± 1.18 nm; *gnat1-1*, 15.98 ± 1.19 nm; *gnat1-2*, 15.93 ± 1.41 nm; *gnat2-1*, 16.25 ± 0.98 nm. **(B)** Average numbers of thylakoid membrane layers per granum determined for wild type, *gnat1-1*, *gnat1-2* and *gnat2-1*. Ten TEM pictures per genotype representing at least five biological replicates were analyzed, whereby all grana stacks of a specific, comparable region of interest were evaluated resulting in final counts of 286 (WT), 300 (*gnat1-1*), 302 (*gnat1-2*), and 313 (*gnat2-1*) analyzed grana. The results were displayed as violin plots illustrating the distribution of data points and showing average values as continuous lines. Differences between genotypes were tested for significance by a One-Way ANOVA approach applying a Fisher LSD test with a significance level of $p \leq 0.05$. The average numbers of membrane layers per granum are as follows (average ± standard

deviation): WT, 5.21 ± 2.36; *gnat1-1*, 4.59 ± 1.74; *gnat1-2*, 4.27 ± 1.43; *gnat2-1*, 5.19 ± 2.27. **(C)** Relative abundances of grana stacks in relation to the corresponding number of thylakoid membrane layers determined for both *gnat1* lines in comparison to wild type and the *gnat2-1* knock-out mutant. The bar plot is based on the dataset represented in (B). Grana with at least three membrane layers were considered for analysis.
