# Supplementary material for: The Plastidial Protein Acetyltransferase GNAT1 Forms a Complex With GNAT2, yet Their Interaction Is Dispensable for State Transitions
Source: Mol Cell Proteomics. 2024 Sep 28;23(11):100850. doi: 10.1016/j.mcpro.2024.100850 (PMC11585782; doi:10.1016/j.mcpro.2024.100850)
Supplement: Suppl. Fig. 9 [file mmc19.pdf]

***A. thaliana* Col-0 wild type**

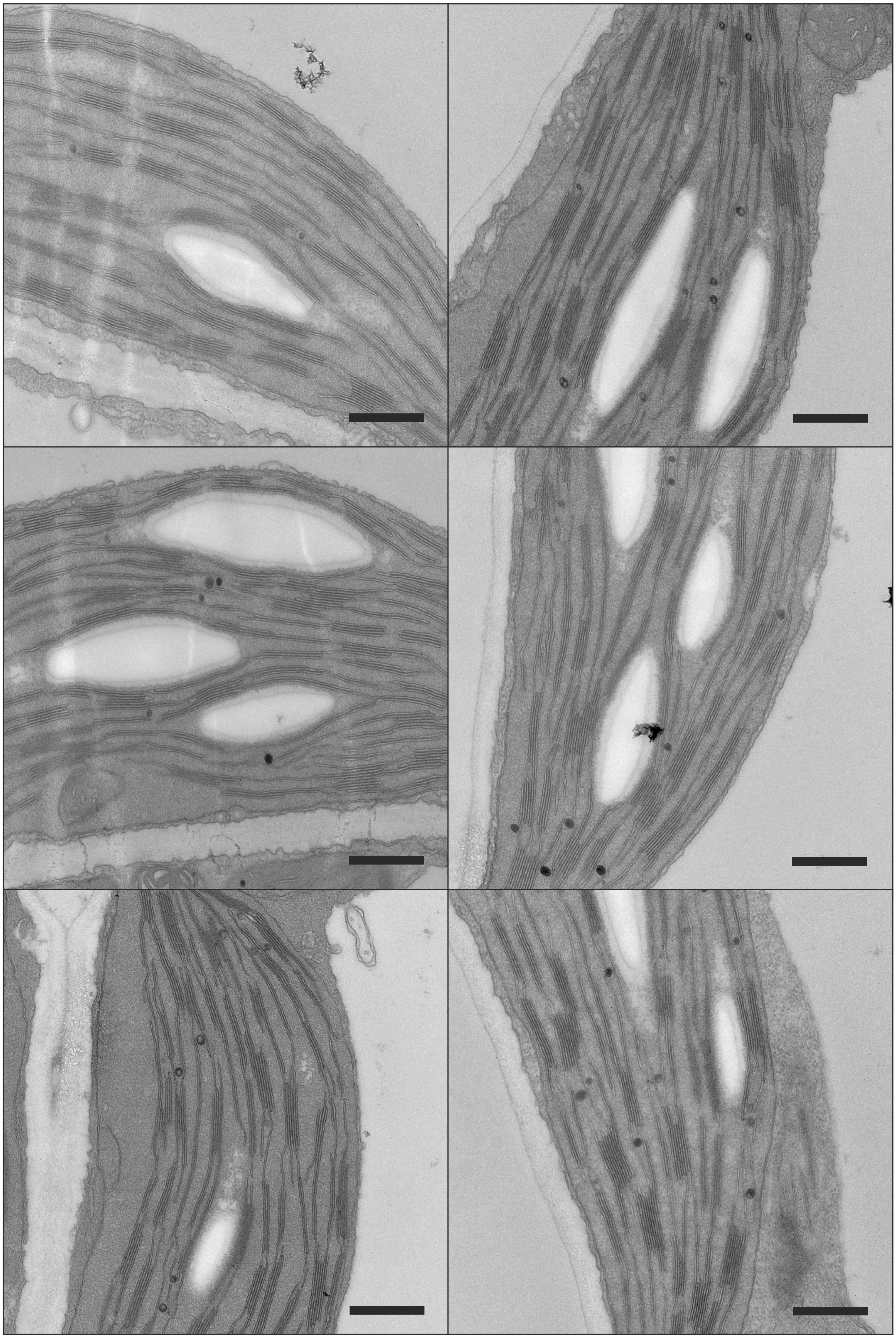

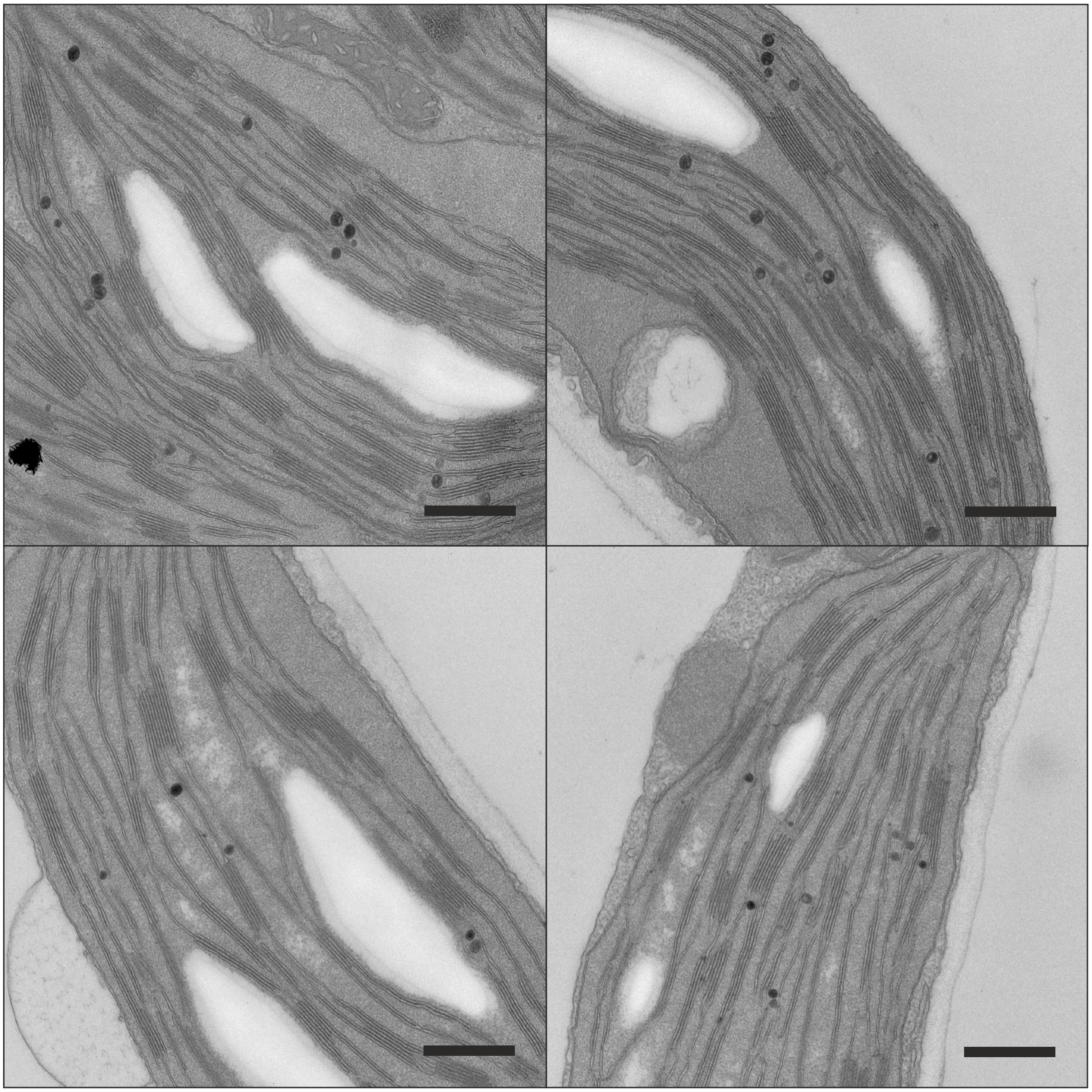

***A. thaliana gnat1-1*** **(SALK_062388)**

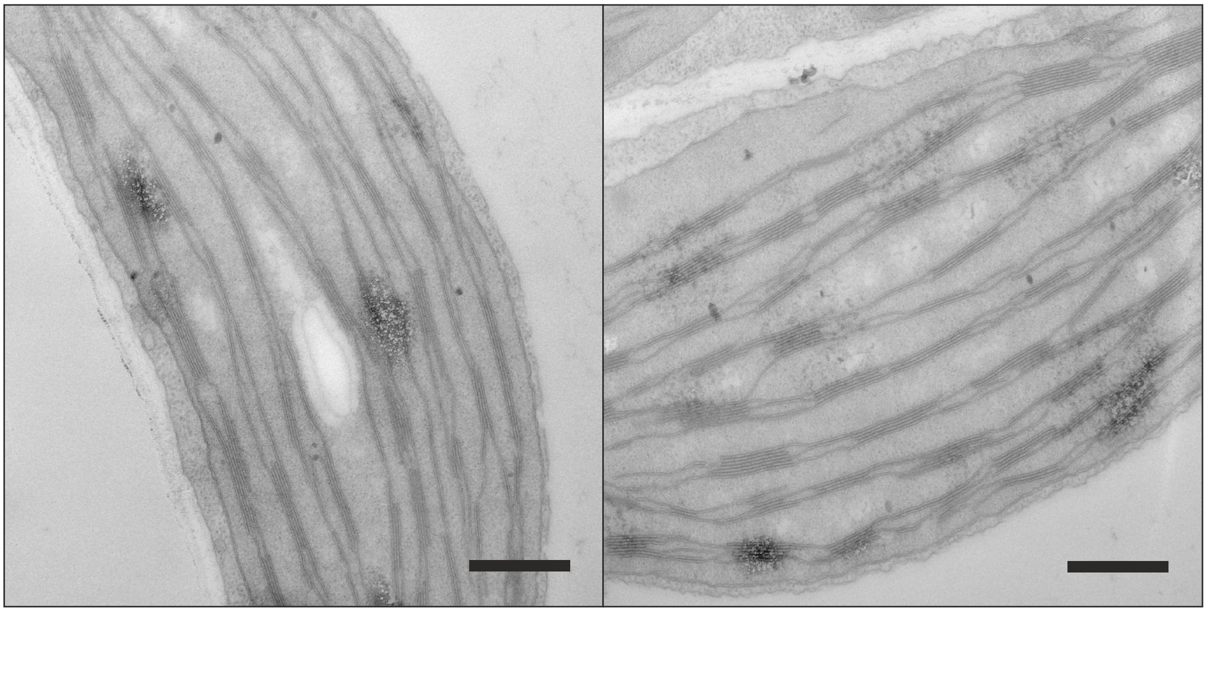

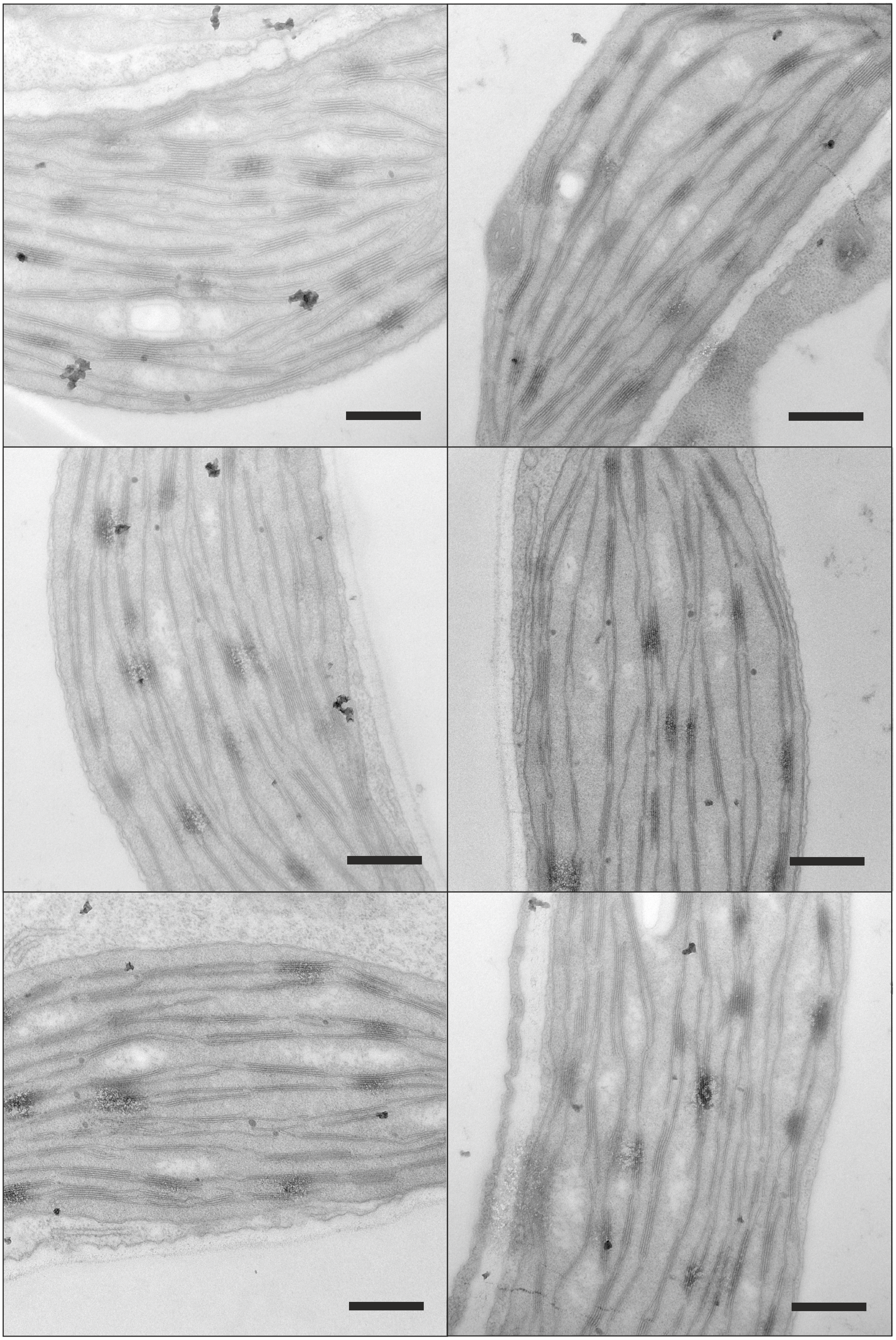

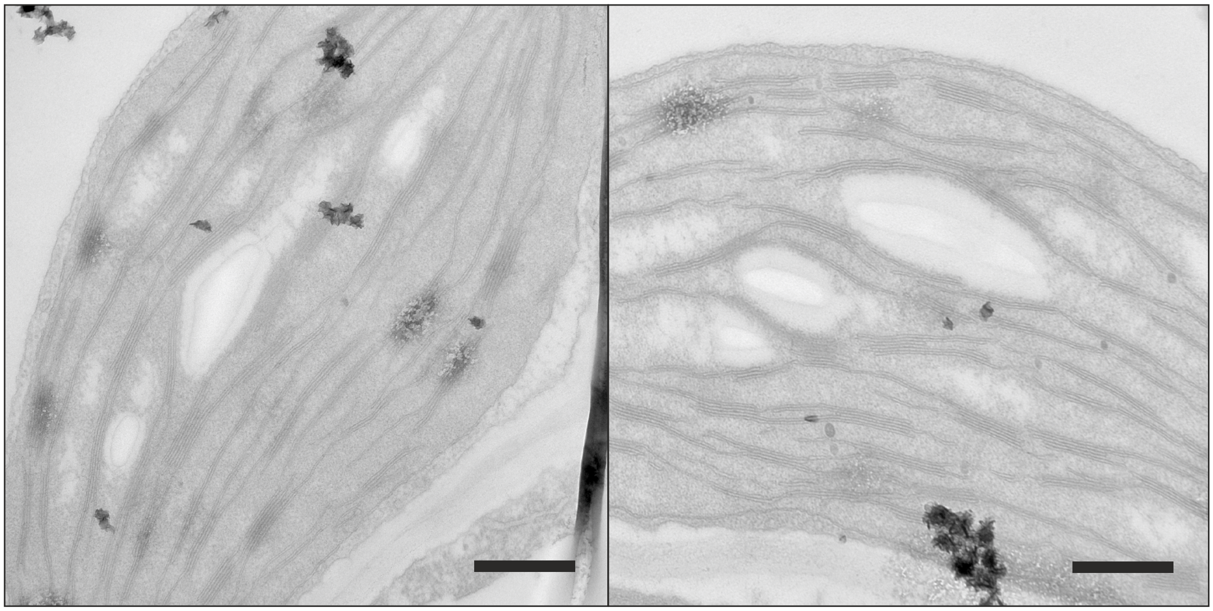

***A. thaliana gnat1-2*** **(SALK_150736)**

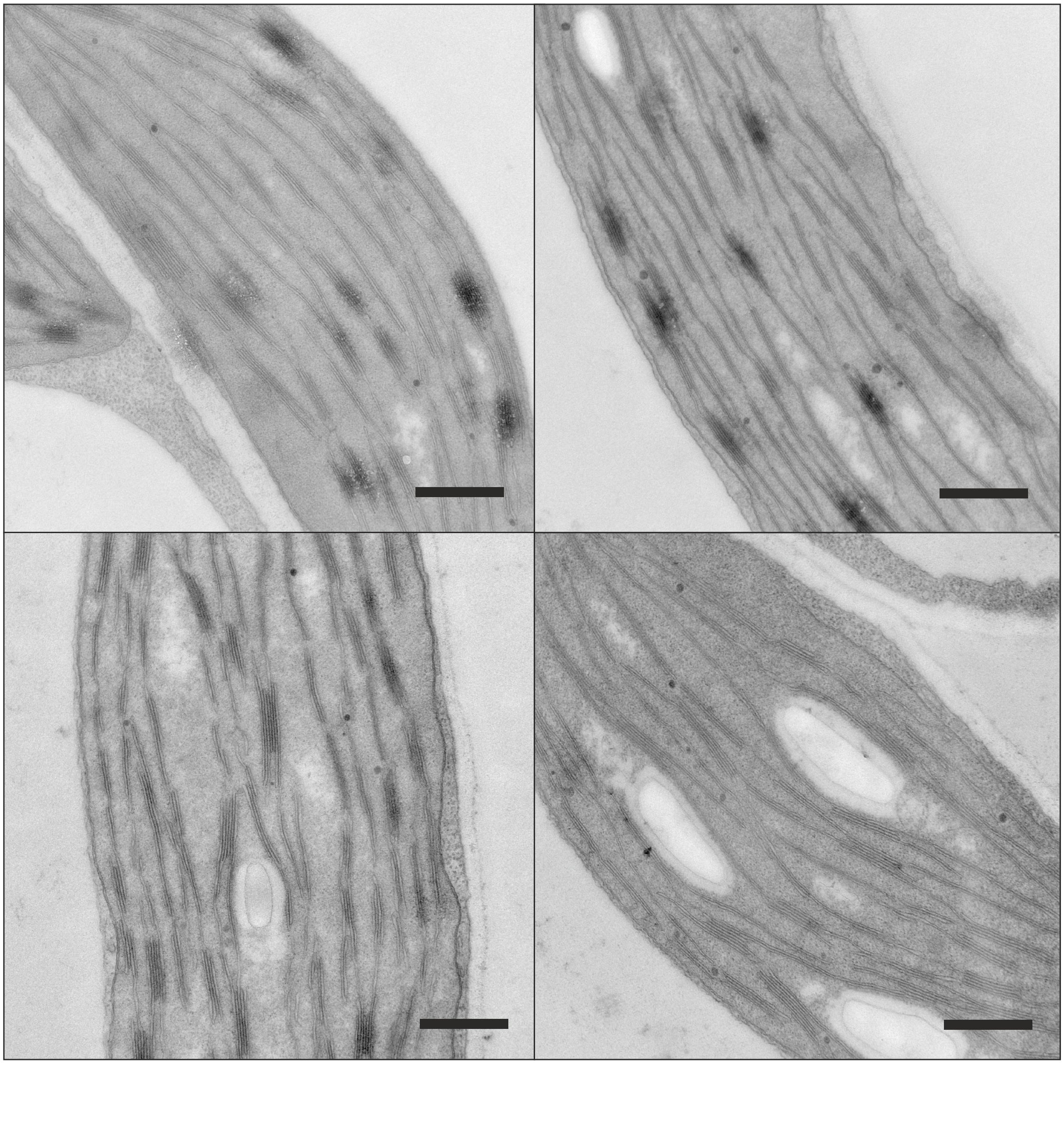

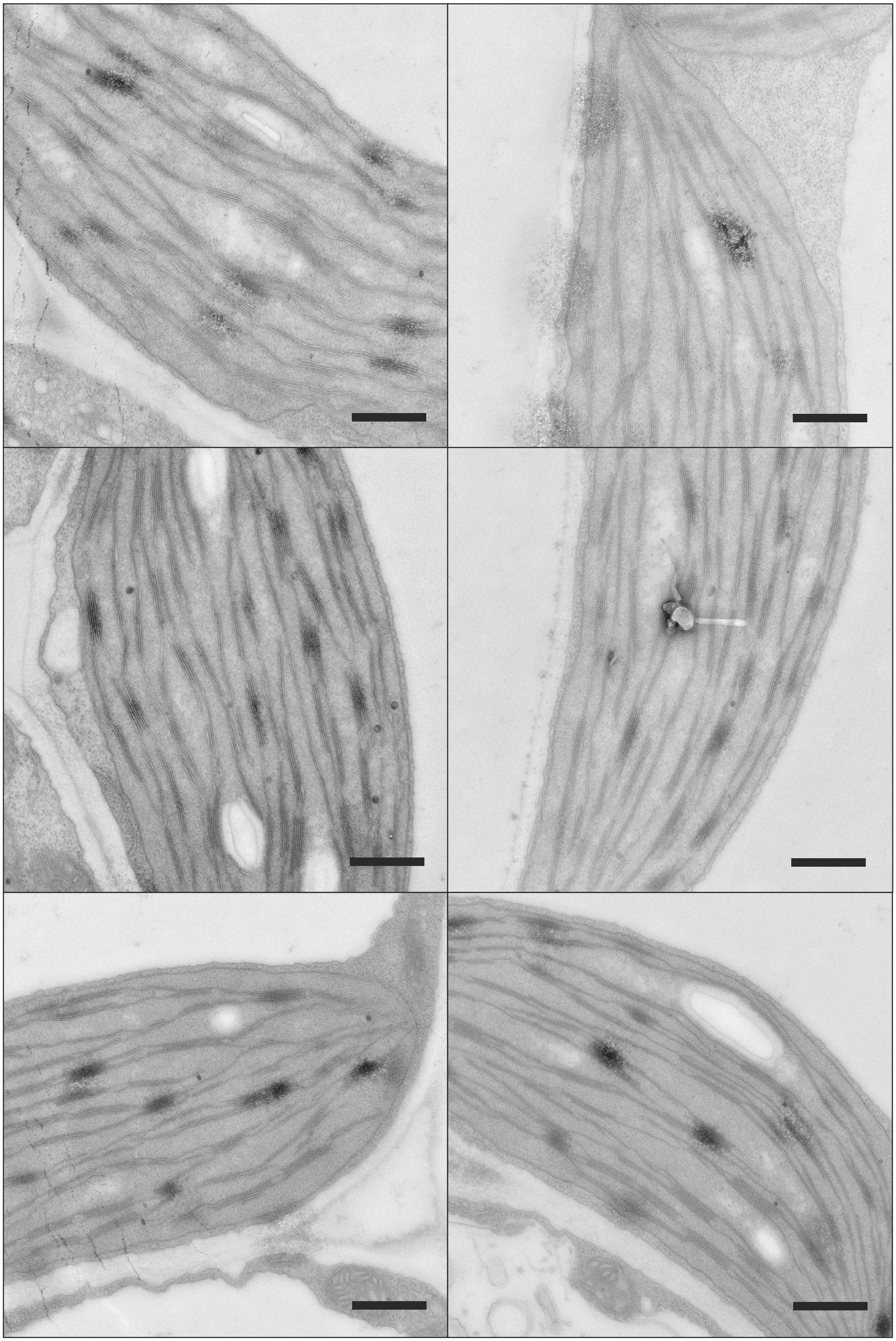

***A. thaliana gnat2-1*** **(SALK_033944)**

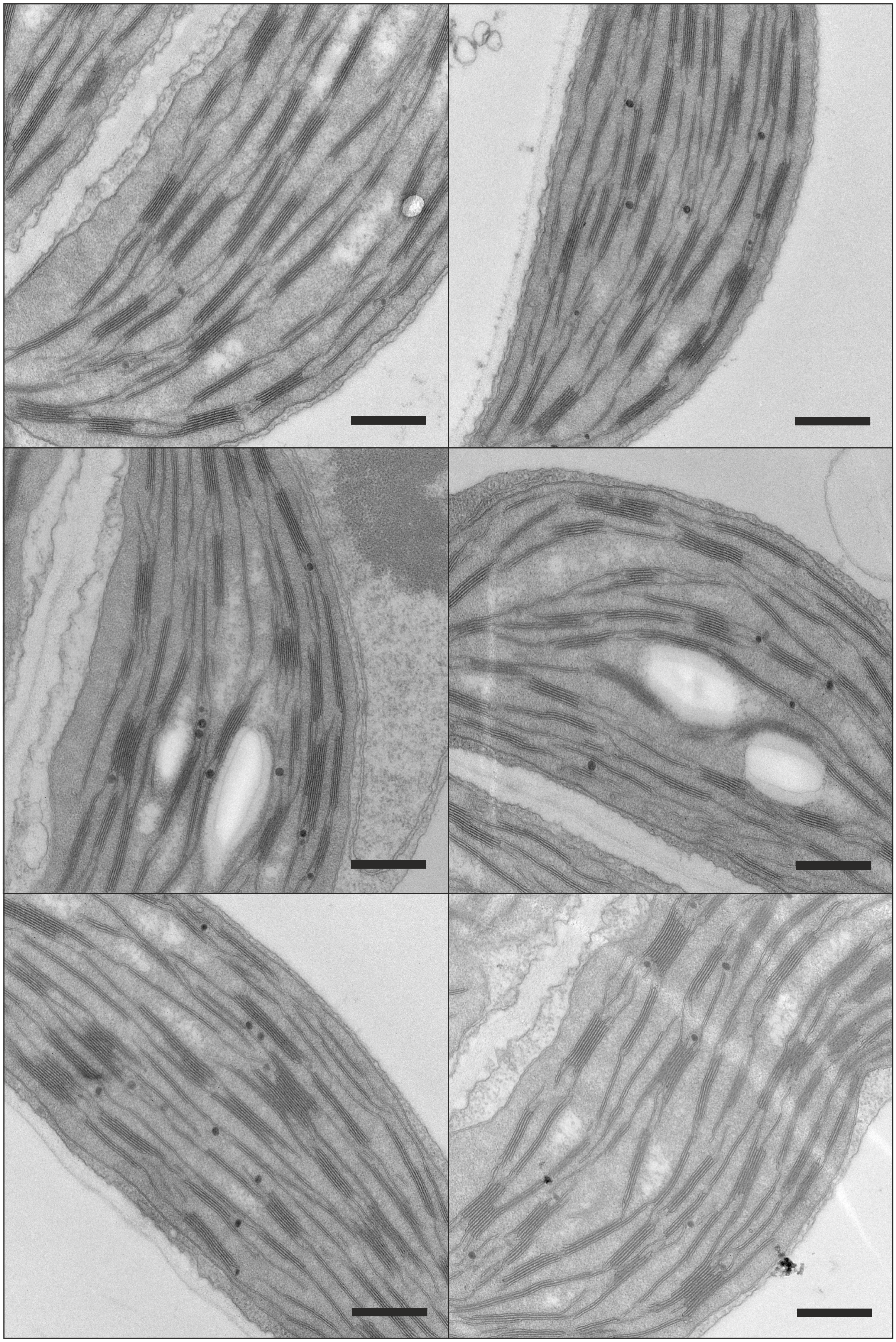

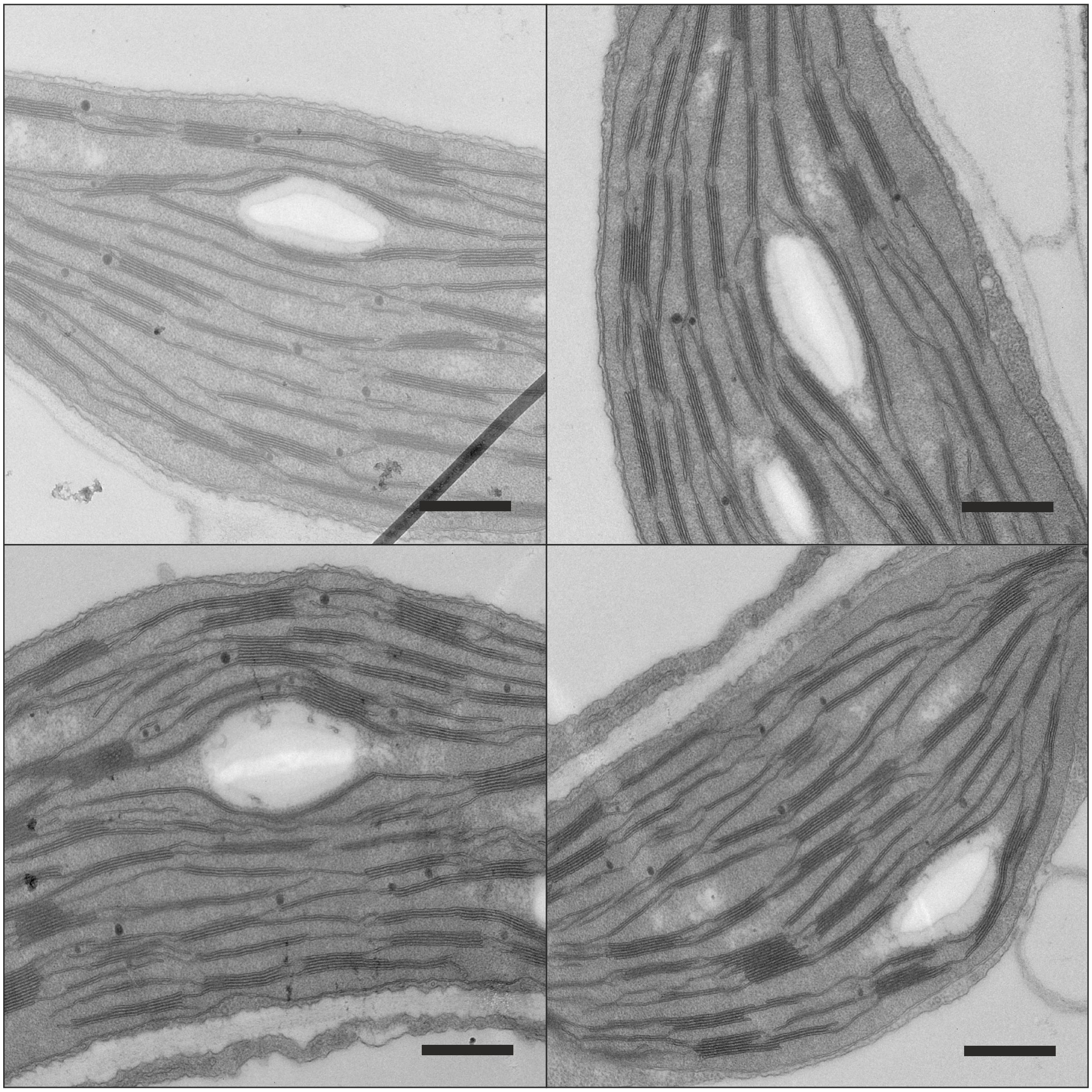

**Supplemental Figure 9. Transmission Electron Microscopy (TEM) images of wild type, *gnat1-1*, *gnat1-2* and *gnat2-1* chloroplasts.** Cells of the palisade mesophyll were selected for imaging and chloroplasts were centered applying a magnification of 10,000x. The scale bar in each panel corresponds to a distance of 500 nm.
