# Supplementary material for: The Plastidial Protein Acetyltransferase GNAT1 Forms a Complex With GNAT2, yet Their Interaction Is Dispensable for State Transitions
Source: Mol Cell Proteomics. 2024 Sep 28;23(11):100850. doi: 10.1016/j.mcpro.2024.100850 (PMC11585782; doi:10.1016/j.mcpro.2024.100850)
Supplement: Suppl. Fig. 10 [file mmc20.pdf]

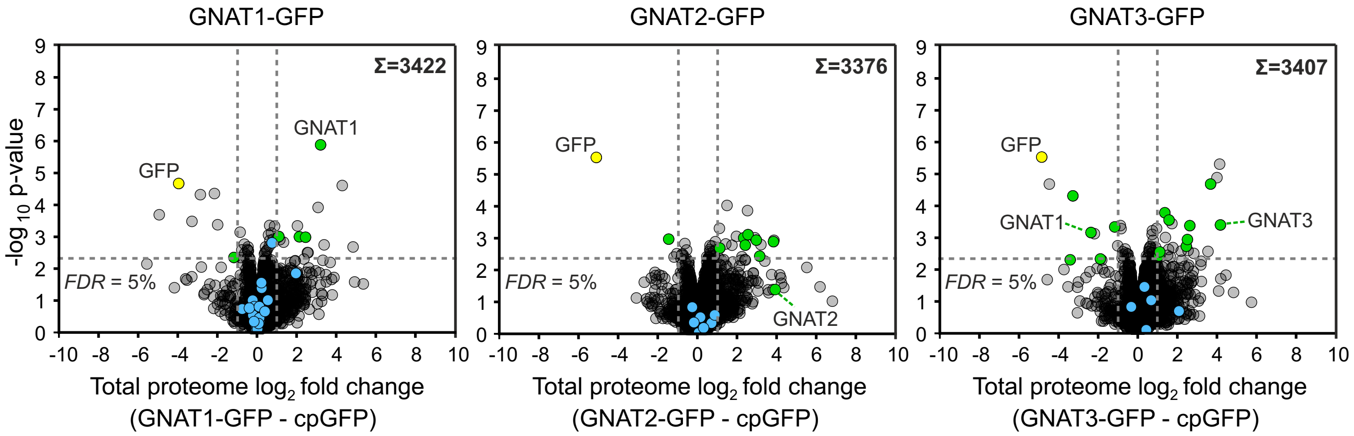

Supplemental Figure 10. Protein abundance profiles of plants oxerexpressing GNAT1-, GNAT2-, or GNAT3-GFP. Sums represent the total number of quantified protein groups per experiment, whereby quantification was achieved by applying the Label-Free-Quantification (LFQ) implementation throughout the data processing procedure via MaxQuant. For each protein group, the $\log_2$ fold change was calculated by comparing LFQ intensities of the respective GNAT-GFP experiment with those of the control experiment comprising the co-immunoprecipitation of chloroplast-targeted GFP (cpGFP). Valid quantifications in at least three of four biological replicates per experiment were required and in addition, significantly up- or downregulated protein groups had to possess a $\log_2$ fold change $\leq$-1 or $\geq$ 1 and a false discovery rate-corrected p-value $\leq$ 0.05 (LIMMA). Green circles highlight protein groups with assigned chloroplast localization and blue circles represent protein groups that were significantly enriched in the corresponding co-immunoprecipitation assays (Fig. 6A, Suppl. Data 6).
