# Supplementary material for: The Plastidial Protein Acetyltransferase GNAT1 Forms a Complex With GNAT2, yet Their Interaction Is Dispensable for State Transitions
Source: Mol Cell Proteomics. 2024 Sep 28;23(11):100850. doi: 10.1016/j.mcpro.2024.100850 (PMC11585782; doi:10.1016/j.mcpro.2024.100850)
Supplement: Suppl. Fig. 11 [file mmc21.pdf]

A

B

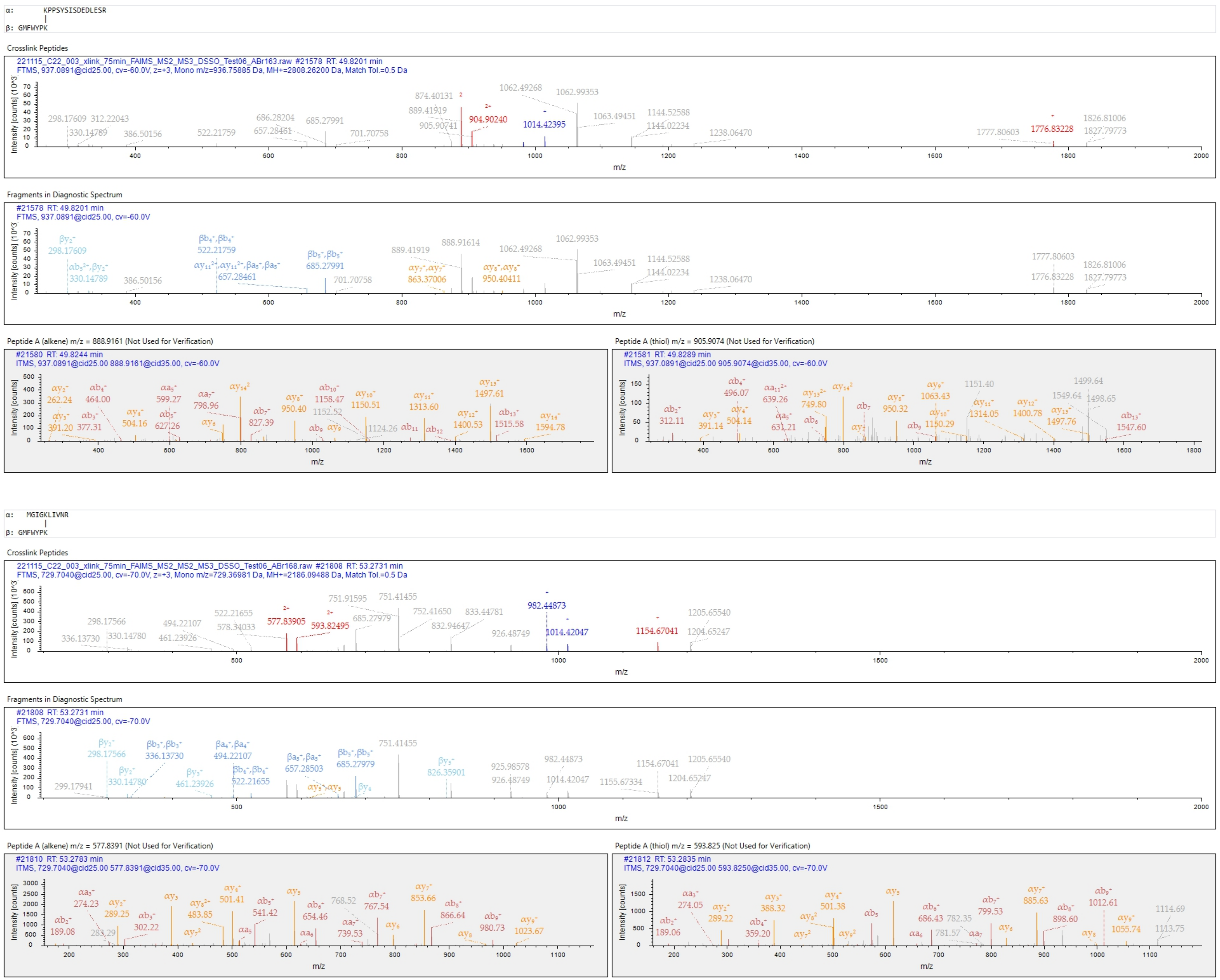
α: KPPSYSISDEDLESR
β: GMFWYPK
Crosslink Peptides
221115_C22_003_xlink_75min_FAIMS_MS2_MS3_DSSO_Test06_ABr163.raw #21578 RT: 49.8201 min
FTMS, 937.0891@cid25.00, cv=-60.0V, z=+3, Mono m/z=936.75885 Da, MH+=2808.26200 Da, Match Tol.=0.5 Da
Fragments in Diagnostic Spectrum
#21578 RT: 49.8201 min
FTMS, 937.0891@cid25.00, cv=-60.0V
Peptide A (alkene) m/z = 888.9161 (Not Used for Verification)
#21580 RT: 49.8244 min
ITMS, 937.0891@cid25.00 888.9161@cid35.00, cv=-60.0V
Peptide A (thiol) m/z = 905.9074 (Not Used for Verification)
#21581 RT: 49.8289 min
ITMS, 937.0891@cid25.00 905.9074@cid35.00, cv=-60.0V
α: MGIGKLIVNR
β: GMFWYPK
Crosslink Peptides
221115_C22_003_xlink_75min_FAIMS_MS2_MS2_MS3_DSSO_Test06_ABr168.raw #21808 RT: 53.2731 min
FTMS, 729.7040@cid25.00, cv=-70.0V, z=+3, Mono m/z=729.36981 Da, MH+=2186.09488 Da, Match Tol.=0.5 Da
Fragments in Diagnostic Spectrum
#21808 RT: 53.2731 min
FTMS, 729.7040@cid25.00, cv=-70.0V
Peptide A (alkene) m/z = 577.8391 (Not Used for Verification)
#21810 RT: 53.2783 min
ITMS, 729.7040@cid25.00 577.8391@cid35.00, cv=-70.0V
Peptide A (thiol) m/z = 593.825 (Not Used for Verification)
#21812 RT: 53.2835 min
ITMS, 729.7040@cid25.00 593.8250@cid35.00, cv=-70.0V
Intensity [counts] (10^3)
Intensity [counts]
m/z

Supplemental Figure 11. Mass spectrometric properties of the peptides that were found to be chemically linked between GNAT1 and GNAT2 (A) or GNAT2 and GNAT3 (B). The chemical crosslinker disuccinimidyl sulfoxide (DSSO) was introduced throughout the co-immunoprecipitation of GNAT1-, GNAT2-, and GNAT3-GFP from leaf material of corresponding overexpressing plant lines. Cross-linked peptides were measured twice using both the “XLMS Cleavable MS2 MS3” and “XLMS Cleavable MS2 MS2 MS3” method templates.
