# Supplementary material for: The Plastidial Protein Acetyltransferase GNAT1 Forms a Complex With GNAT2, yet Their Interaction Is Dispensable for State Transitions
Source: Mol Cell Proteomics. 2024 Sep 28;23(11):100850. doi: 10.1016/j.mcpro.2024.100850 (PMC11585782; doi:10.1016/j.mcpro.2024.100850)
Supplement: Suppl. Fig. 12 [file mmc22.pdf]

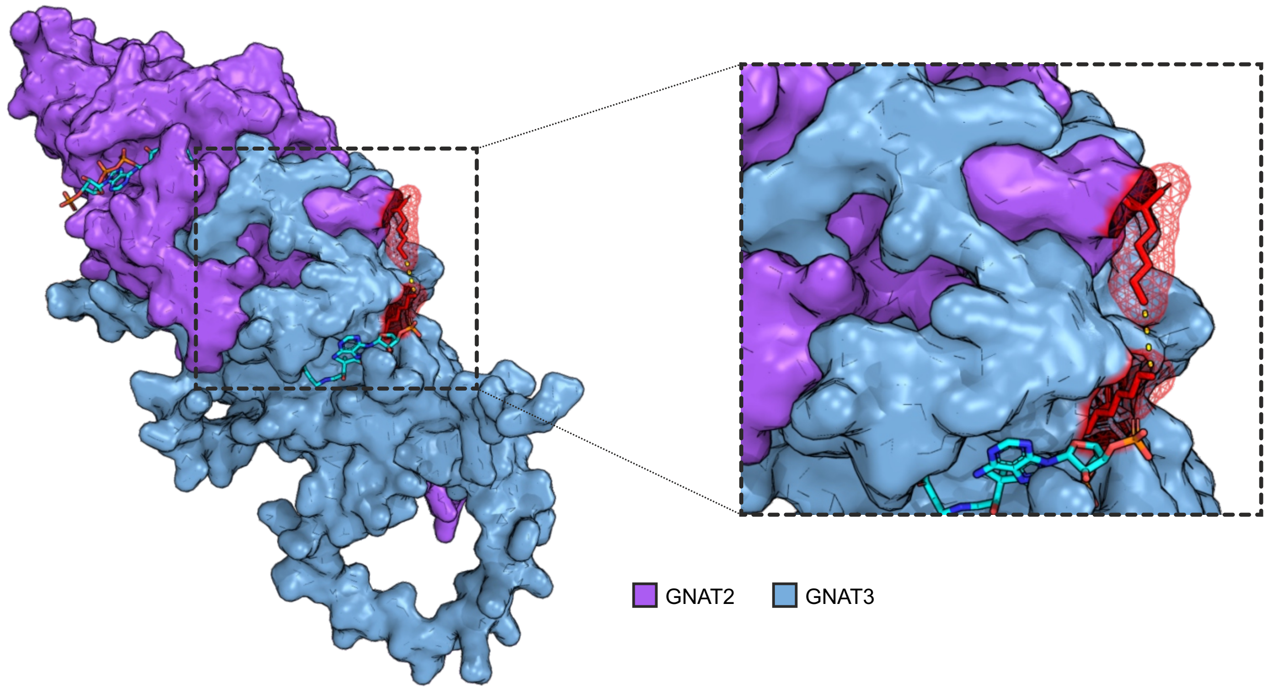

Supplemental Figure 12. Structure prediction for the heterodimer GNAT2-GNAT3. The structure model was derived by using AlphaFold 2 Multimer (43). Sequence data were obtained from the Araport 11 database and N-terminally truncated by the amino acid sequence corresponding to predicted chloroplast transit peptides. The lysine residues involved in the formation of the identified DSSO protein crosslink were shown as red stick models covered by the red, partially transparent mesh structure of the computed protein surface. The dotted yellow line marks the shortest distance between the two amino groups of the lysine pair, whereby a value of 5.1 Å was calculated. The positions of the CoA molecules presented as stick models were derived from structure predictions provided by the AlphaFill database (41).
