# Supplementary material for: The Plastidial Protein Acetyltransferase GNAT1 Forms a Complex With GNAT2, yet Their Interaction Is Dispensable for State Transitions
Source: Mol Cell Proteomics. 2024 Sep 28;23(11):100850. doi: 10.1016/j.mcpro.2024.100850 (PMC11585782; doi:10.1016/j.mcpro.2024.100850)
Supplement: Suppl. Fig. 13 [file mmc23.pdf]

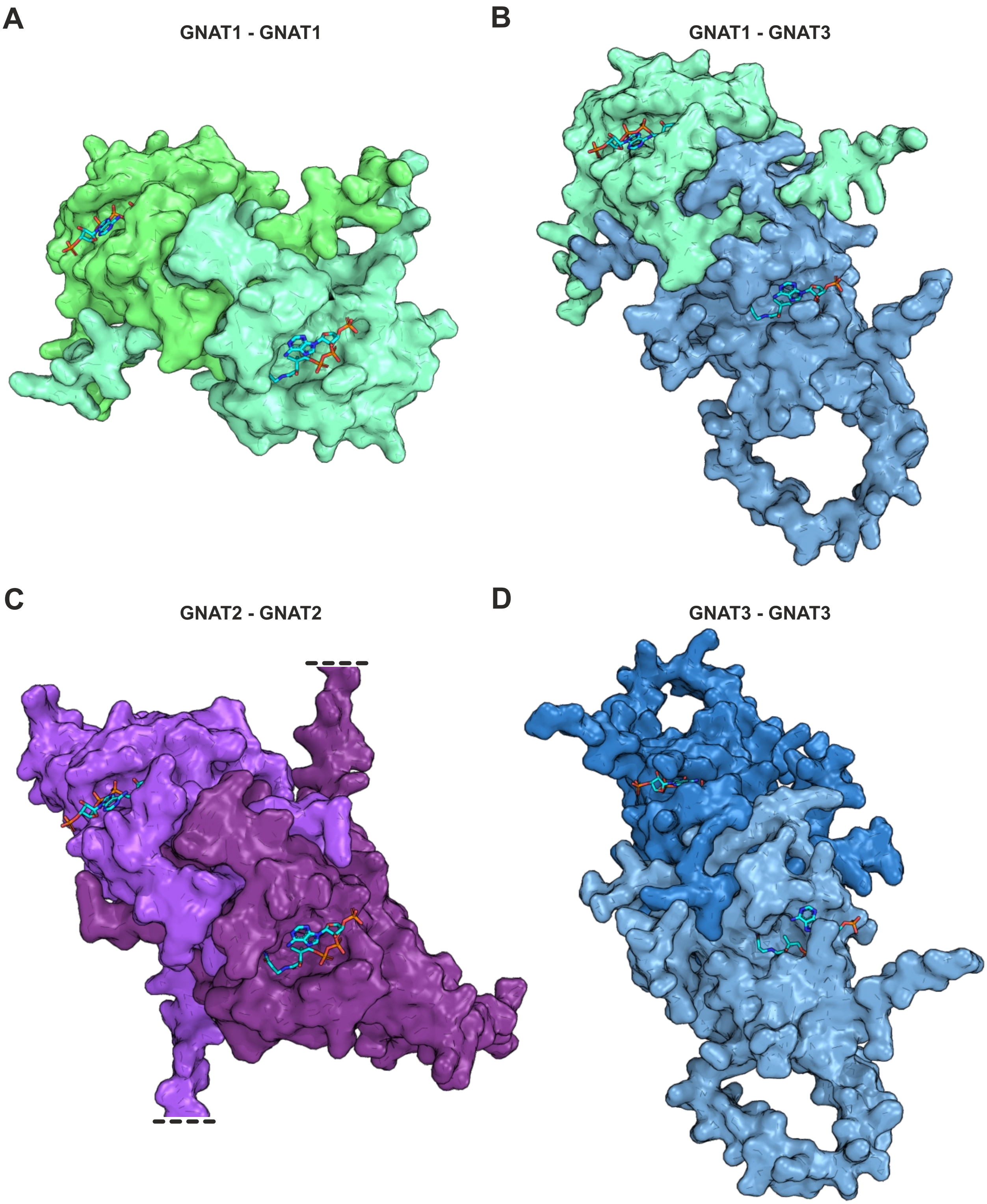

Supplemental Figure 13. Structure predictions for experimentally unconfirmed GNAT homo- and heterodimers. Structure models of the dimer constellations GNAT1-GNAT1 **(A)**, GNAT1-GNAT3 **(B)**, GNAT2-GNAT2 **(C)** and GNAT3-GNAT3 **(D)** predicted by AlphaFold 2 Multimer and visualized via PyMOL (43). Sequence data were obtained from the Araport 11 database and N-terminally truncated by the amino acid sequences corresponding to the respectively predicted chloroplast transit peptide. Positions of the CoA molecules presented as stick models were derived from structure predictions provided by the AlphaFill database (41). All obtained structures reveal a high all-over confidence of more than 0.8 as indicated by intrinsic modeling scores evaluated by AlphaFold 2 Multimer (Table 3, Suppl. Table 4).
