# Supplementary material for: The Plastidial Protein Acetyltransferase GNAT1 Forms a Complex With GNAT2, yet Their Interaction Is Dispensable for State Transitions
Source: Mol Cell Proteomics. 2024 Sep 28;23(11):100850. doi: 10.1016/j.mcpro.2024.100850 (PMC11585782; doi:10.1016/j.mcpro.2024.100850)
Supplement: Suppl. Fig. 14 [file mmc24.pdf]

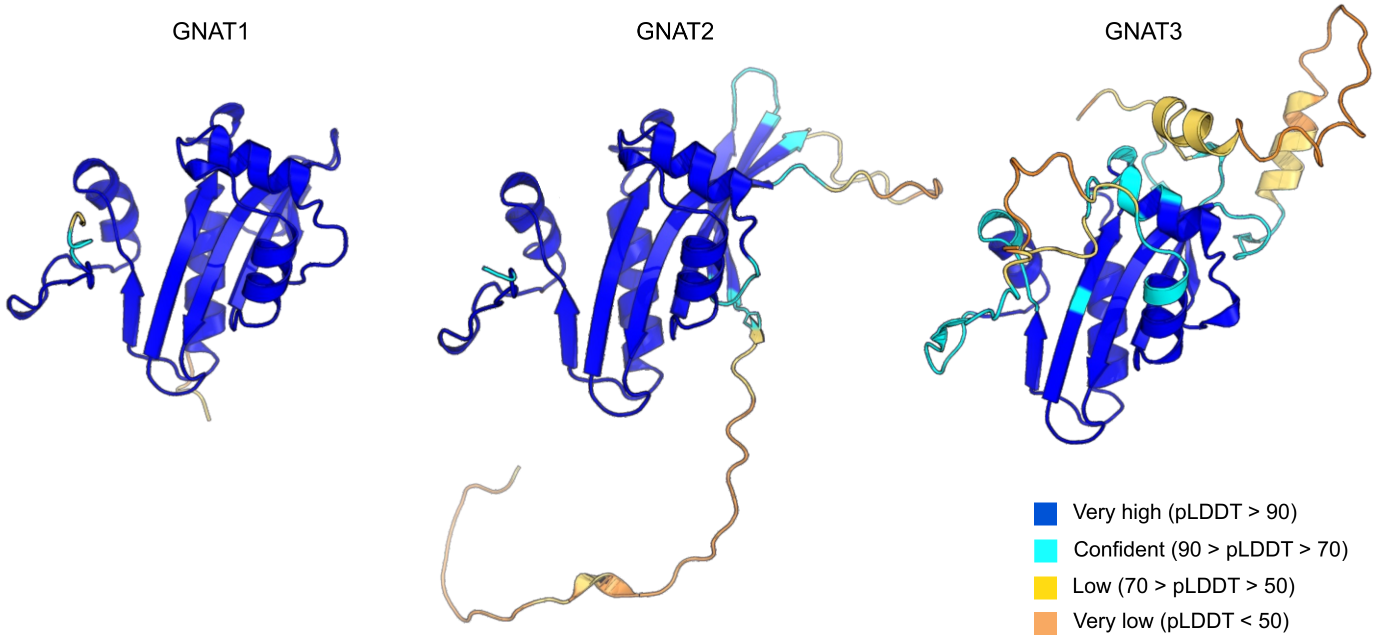

Supplemental Figure 14: AlphaFold 2 generated structure models of monomeric GNAT1, GNAT2, and GNAT3 colored according to the corresponding levels of per-residue accuracy. The color code indicates the ranges of per-residue accuracy calculated in the form of a predicted local-distance difference test (pLDDT) throughout the modeling procedure (42). Each GNAT model was trimmed by the N-terminal region representing the plastid transit peptide.
