# Supplementary material for: The Plastidial Protein Acetyltransferase GNAT1 Forms a Complex With GNAT2, yet Their Interaction Is Dispensable for State Transitions
Source: Mol Cell Proteomics. 2024 Sep 28;23(11):100850. doi: 10.1016/j.mcpro.2024.100850 (PMC11585782; doi:10.1016/j.mcpro.2024.100850)
Supplement: Suppl. Fig. 15 [file mmc25.pdf]

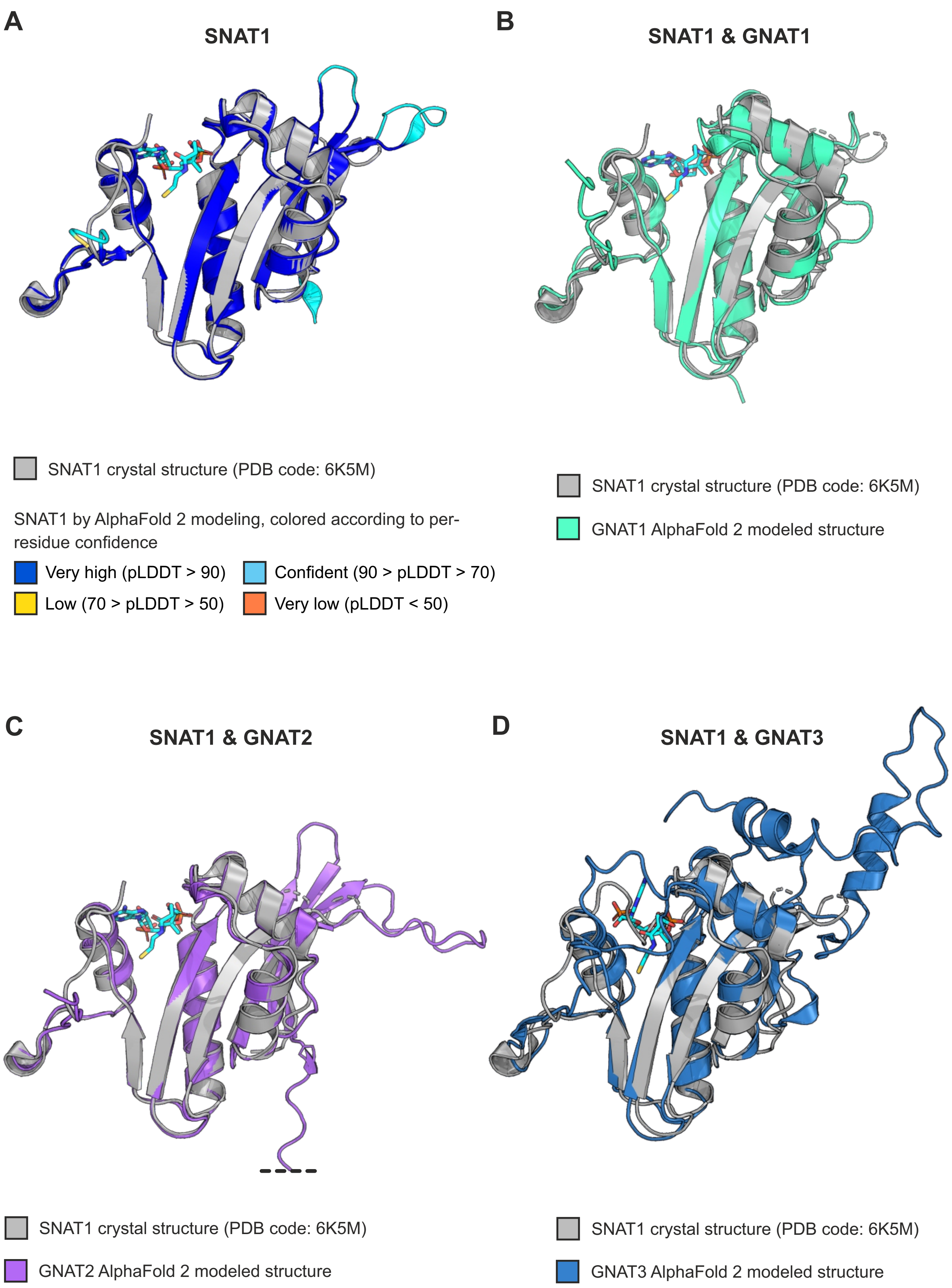
A
SNAT1
B
SNAT1 & GNAT1
SNAT1 crystal structure (PDB code: 6K5M)
SNAT1 by AlphaFold 2 modeling, colored according to per-residue confidence
Very high (pLDDT > 90)
Confident (90 > pLDDT > 70)
Low (70 > pLDDT > 50)
Very low (pLDDT < 50)
SNAT1 crystal structure (PDB code: 6K5M)
GNAT1 AlphaFold 2 modeled structure
C
SNAT1 & GNAT2
D
SNAT1 & GNAT3
SNAT1 crystal structure (PDB code: 6K5M)
GNAT2 AlphaFold 2 modeled structure
SNAT1 crystal structure (PDB code: 6K5M)
GNAT3 AlphaFold 2 modeled structure

E

SNAT1 homodimer & GNAT2 homodimer

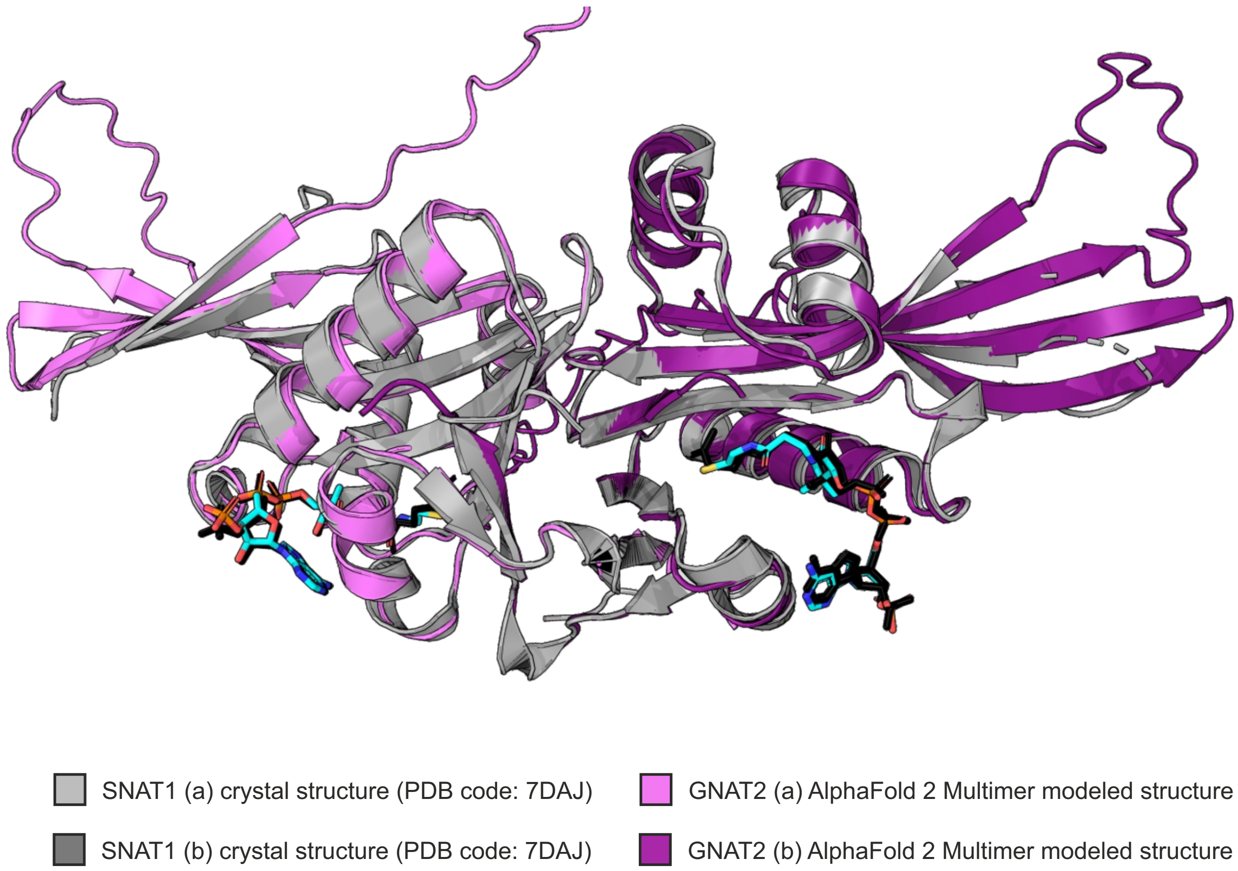

Supplemental Figure 15: Superimpositions of experimentally resolved crystal structures of the acetyltransferase SNAT1 from *Oryza sativa* with structure models of SNAT1 as well as of GNAT1, GNAT2, and GNAT3, which were generated by AlphaFold 2 or AlphaFold 2 Multimer (41-43, 89). Crystal structure data were obtained from the RCSB Protein Data Bank (PDB code: 6K5M, SNAT1 monomer; 7DAJ, SNAT1 homodimer with co-substrate ac-CoA; [89]). Monomer superimpositions of modeled SNAT1 **(A)**, GNAT1 **(B)**, GNAT2 **(C)**, or GNAT3 **(D)** with the experimentally derived SNAT1 structure were displayed, whereby the color-code of the modelled SNAT1 illustrates the per-residue confidence level of orientation and local distance (pLDDT; [42]). Furthermore, panel **(E)** shows an overlay of the crystal structure of a SNAT1 homodimer equipped with its cofactor ac-CoA and the modelled GNAT2 homodimer. The cofactor CoA was integrated into the GNAT models via the AlphaFill tool and presented as colored stick model (41), whereas the ac-CoA molecule of the SNAT1 homodimer structure is depicted in black color. Superimposition and visualisation of structures were performed using PyMOL (Version 4.5 Schrödinger, LLC).
