# Supplementary material for: The Plastidial Protein Acetyltransferase GNAT1 Forms a Complex With GNAT2, yet Their Interaction Is Dispensable for State Transitions
Source: Mol Cell Proteomics. 2024 Sep 28;23(11):100850. doi: 10.1016/j.mcpro.2024.100850 (PMC11585782; doi:10.1016/j.mcpro.2024.100850)
Supplement: Suppl. Fig. 16 [file mmc26.pdf]

```
AtGNAT3_trunc   ---------------------------MGLVGCV---------------GKSSLVSMEL-   17
AtGNAT1         ---------------------------MFLGGTI----------------STPPASLRLR   17
AtGNAT2         MLLIPISSSSSSSIPPPNSYPSNHH---SL--------FFSNLTFPIQHGSRKLKTLRLR   49
OsSNAT1         --MASAASASASAVVTPSSFRCVPTASCGLGARGKAPAPRRLLHDHAQGKKRAAATWSLK   58

AtGNAT3_trunc   --RWARRRKS----D----------------------------NAASALPRSIPIY----   39
AtGNAT1         S--------TLNPQNAVTQSSSQATFPAAMQRKPPSYSISDE----------------DL   53
AtGNAT2         ANFWESIRSGFVKNNNST-----------QLVEPPSIVNDEEEETEPLLPVEFTLVERNL   98
OsSNAT1         AGLWDSLRSGFLKSNNST-----------ETVEPPSAPIEEE----EPLPEELVLLERTL   103

                      β1             α1                             α2
AtGNAT3_trunc   --------ISTLKKDINLEELRNLYSLCNHSCNRLSEKDSNVEKIVDMKKLRRAISRSDV   91
AtGNAT1         ES--RGFLLRRTTEGLNLDQLNS---VFAA----VGFPRR------DTAKIEVALQHTDA   98
AtGNAT2         EDGLVEEIIFSSGGEIDVYDLQG---LCDK----VGWPRR------PLVKLAAALKNSYM   145
OsSNAT1         ADGSTEQIIFSSAGDVNVYDLQA---LCDK----VGWPRR------PLTKIAASLRNSYL   150

                  β2                            β3            β4
AtGNAT3_trunc   IVSVFC--KPQ-------H/VPLTPSNGQLVGFGRAYSDYGLTASIHDLMVLPSLQRMGI   182
AtGNAT1         LLW------------------VEYEKTRRPVAFARATGDGVFNAIIWDVVVDPSFQSCGL   140
AtGNAT2         VATLHSVMKSSSDSDSSEGGDGEKQQEKKLIGMARATSDHAFNATIWDVLVDPEYQGQGL   205
OsSNAT1         VATLHSVTTPSK---------AEGEERKQLIGMARATSDHAFNATIWDVLVDPSYQGQGL   201

                     α3              β5        α4
AtGNAT3_trunc   GKLIVNRIVRLLTSRDIYDIAALCFEDERPFFKACGFGDDRMGSTTMMFTKSLEA--   237
AtGNAT1         GKAVMERLIEDLQVKGICNIALYSEPRVLGFYRPLGFVSDPDGIKGMVFIRKQRNKK   197
AtGNAT2         GKALVEKLVRALLQRDIGNISLFADSQVVDFYQNLGFEADPEGIKGMFWYPK-----   257
OsSNAT1         GKALMEKVIRTLLQRDISNITLFADNKVVDFYKNLGFEADPQGIKGMFWYPRF----   254
```

Supplemental Figure 16: Alignment of the amino acid sequences of GNAT1, GNAT2 and GNAT3 from *Arabidopsis thaliana* and SNAT1 from *Oryza sativa*. In case of GNAT1, 2 and 3, sequences including the predicted transit peptide regions were obtained from the Araport 11 database and refer to spliceform 1, respectively. For SNAT1, the amino acid sequence described in the study of Liao and coworkers was used (89). Red boxes represent α-helix structures, whereas blue boxes indicate the formation of β-sheets. Predicted chloroplast transit peptides were marked in grey. Since GNAT3 exhibits an extensive additional loop between β2 and β3, the corresponding amino acid sequence was removed prior to the alignment as indicated by a slash highlighted in green (the full GNAT3 sequence can be found in Suppl. Fig. 17).
