# Supplementary material for: The Plastidial Protein Acetyltransferase GNAT1 Forms a Complex With GNAT2, yet Their Interaction Is Dispensable for State Transitions
Source: Mol Cell Proteomics. 2024 Sep 28;23(11):100850. doi: 10.1016/j.mcpro.2024.100850 (PMC11585782; doi:10.1016/j.mcpro.2024.100850)
Supplement: Suppl. Fig. 17 [file mmc27.pdf]

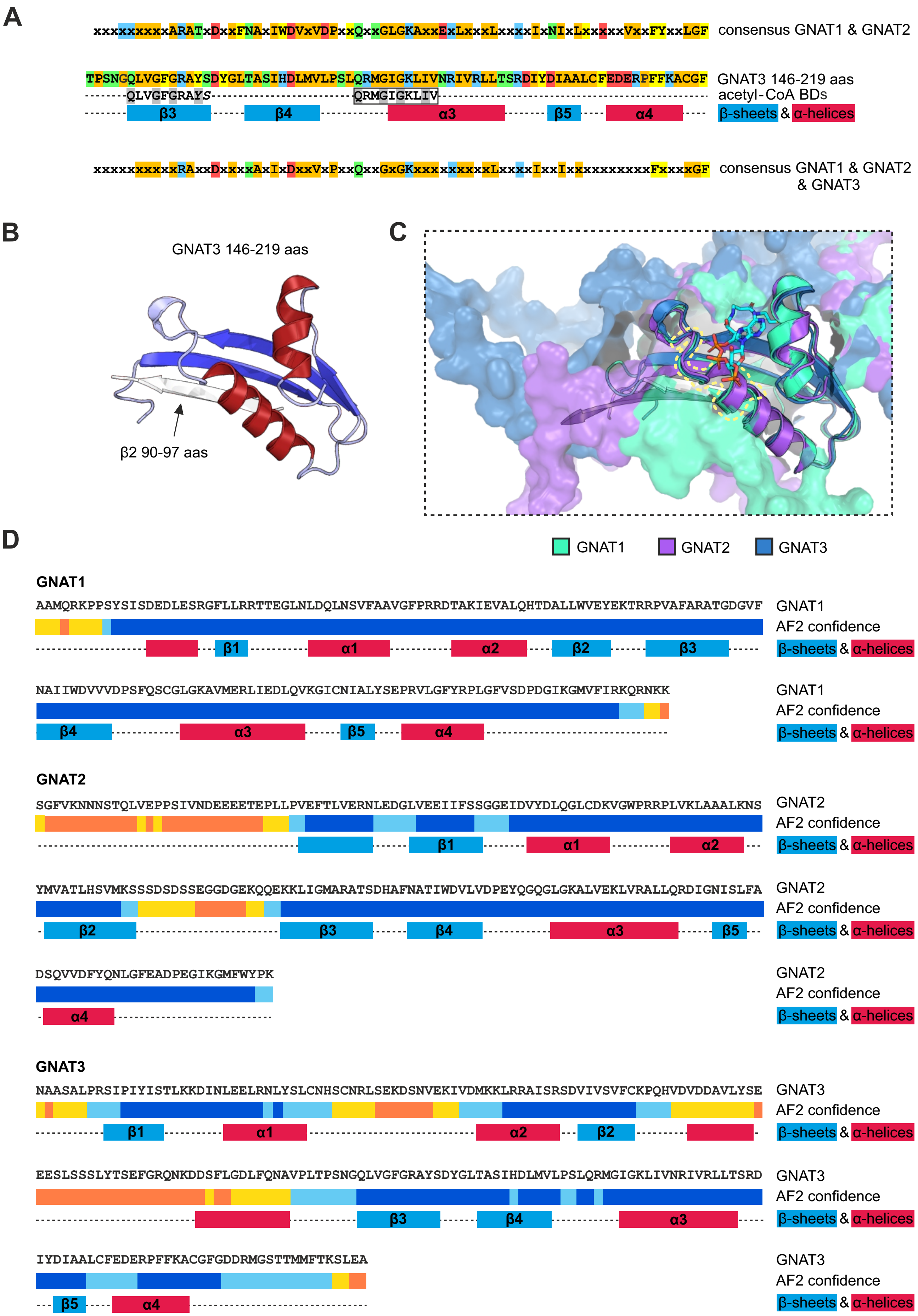

A
xxxxxxxxxARATxDxxFNAxIWDVxVDPxxQxxGLGKAxxExLxxxLxxxxIxNIxLxxxxxxVxxFYxxLGF
consensus GNAT1 & GNAT2
TPSNGQLVGFGRAYSDYGLTASIHDLMVLPSLQRMGIGKLIVNRIVRLLTSRDIYDIAALCFEDERPFFKACGF
GNAT3 146-219 aas
QLVGFGRAYS
QRMGIGKLIV
acetyl-CoA BDs
β3
β4
α3
β5
α4
β-sheets & α-helices
xxxxxxxxxxRAxxDxxxxAxIxDxxVxPxxQxxGxGKxxxxxxxxxLxxxxIxxIxxxxxxxxxxFxxxxGF
consensus GNAT1 & GNAT2 & GNAT3
B
GNAT3 146-219 aas
β2 90-97 aas
C
GNAT1
GNAT2
GNAT3
D
GNAT1
AAMQRKPPSYSISDEDLESRGFLLRRTTEGLNLDQLNSVFAAVGFPRRDTAKIEVALQHTDALLWVEYEKTRRPVAFARATGDGVF
GNAT1
AF2 confidence
β1
α1
α2
β2
β3
β-sheets & α-helices
NAIIWDVVVDPSFQSCGLGKAVMERLIEDLQVKGICNIALYSEPRVLGFYRPLGFVSDPDGIKGMVFIRKQRNKK
GNAT1
AF2 confidence
β4
α3
β5
α4
β-sheets & α-helices
GNAT2
SGFVKNNNSTQLVEPPSIVNDEEEETEPLLPVEFTLVERNLEDGLVEEIIFSSGGEIDVYDLQGLCDKVGWPRRPLVKLAAALKNS
GNAT2
AF2 confidence
β1
α1
α2
β-sheets & α-helices
YMVATLHSVMKSSSDSDSSEGGDGEKQQEKKLIGMARATSDHAFNATIWDVLVDPEYQGQGLGKALVEKLVRALLQRDIGNISLFA
GNAT2
AF2 confidence
β2
β3
β4
α3
β5
β-sheets & α-helices
DSQVVDFYQNLGFEADPEGIKGMFWYPK
GNAT2
AF2 confidence
α4
β-sheets & α-helices
GNAT3
NAASALPRSIPIYISTLKKDINLEELRNLYSLCNHSCNRLSEKDSNVEKIVDMKKLRRAISRSDVIVSVFCKPQHVDVDDAVLYSE
GNAT3
AF2 confidence
β1
α1
α2
β2
β-sheets & α-helices
EESLSSSLYTSEFGRQNKDDSFLGDLFQNAVPLTPSNGQLVGFGRAYSDYGLTASIHDLMVLPSLQRMGIGKLIVNRIVRLLTSRD
GNAT3
AF2 confidence
β3
β4
α3
β-sheets & α-helices
IYDIAALCFEDERPFFKACGFGDDRMGSTTMMFTKSLEA
GNAT3
AF2 confidence
β5
α4
β-sheets & α-helices

Supplemental Figure 17. Structural organization of the acetyltransferase domain of GNAT3 and schematic representation of secondary structure elements. Per-residue confidences were calculated by AlphaFold 2 in the process of structure prediction (42). **(A)** Overview of the primary and secondary structure of the Pfam acetyltransferase domain of GNAT3 (PF00583, aligned region; [80]) in comparison to the consensus sequence derived from the GNAT1 and GNAT2 analysis (Fig. 2 A). In the third row, an additional consensus sequence is displayed indicating the amino acids that are identical (in one-letter-code) or different (x) between the GNAT3 sequence and the GNAT1-GNAT2 consensus sequence. Furthermore, colors highlighting the different letters classify the amino acid side chains as follows: orange, non-polar; yellow, aromatic; green, polar (neutral); blue, positively charged, and red, negatively charged. The region of acetyl-CoA binding in GNAT3 is displayed below the primary amino acid sequence with the boxed region representing the main binding site (26). **(B)** Structure model of the GNAT3 acetyltransferase domain obtained from the AlphaFold 2 database and visualized via PyMOL. The arrow marks β-sheet 2, which is not annotated as part of the Pfam acetyltransferase domain, but contributes to the formation of the GNAT specific, V-shaped core element. **(C)** Superimposition of the modeled acetyltransferase domains of GNAT1, GNAT2 and GNAT3. The structure models were extended by a stick model of the cofactor CoA with the help of the AlphaFill repository (41). Moreover, the surrounding protein surface of GNAT1, 2 and 3 were shown as slightly transparent areas. The dashed yellow line highlights the main acetyl-CoA binding site of all three GNATs. **(D)** Structural organization of monomeric GNAT1, 2 and 3 as predicted by AlphaFold 2 (excluding predicted transit peptide sequences). The first rows show the corresponding amino acid sequences, whereas the second rows indicate the per-residue accuracy for the derived structure models. The AlphaFold 2 per-residue confidence is given in the ranges “Very high” (pLDDT > 90), dark blue; “Confident” (90 > pLDDT > 70), light blue; “Low” (70 > pLDDT >50), yellow; “Very low” (pLDDT < 50), orange (42). The third rows highlight the residues, which are proposed to form α-helix (red) and β-sheet (blue) structural elements.
