# Supplementary material for: The Plastidial Protein Acetyltransferase GNAT1 Forms a Complex With GNAT2, yet Their Interaction Is Dispensable for State Transitions
Source: Mol Cell Proteomics. 2024 Sep 28;23(11):100850. doi: 10.1016/j.mcpro.2024.100850 (PMC11585782; doi:10.1016/j.mcpro.2024.100850)
Supplement: Suppl. Fig. 18 [file mmc28.pdf]

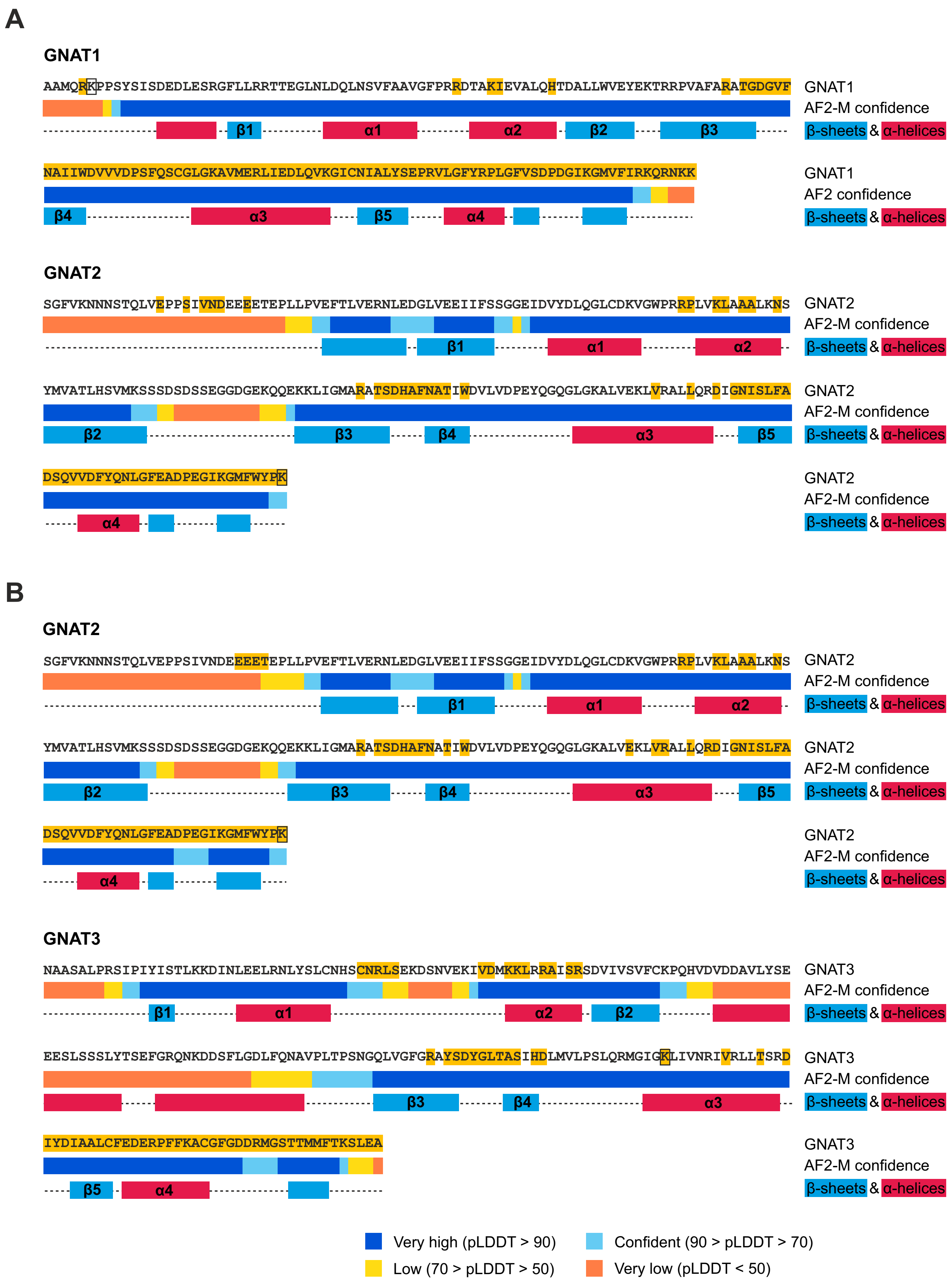
A
GNAT1
AAMQRKPPSYSISDEDLESRGFLLRRTTEGLNLDQLNSVFAAVGFPRRDTAKIEVALQHTDALLWVEYEKTRRPVAFARATGDGVF
GNAT1
AF2-M confidence
β1 α1 α2 β2 β3
β-sheets & α-helices
NAIIWDVVVDPSFQSCGLGKAVMERLIEDLQVKGICNIALYSEPRVLGFYRPLGFVSDPDGIKGMVFIRKQRNKK
GNAT1
AF2 confidence
β4 α3 β5 α4
β-sheets & α-helices
GNAT2
SGFVKNNNSTQLVEPPSIVNDEEEETEPLLPVEFTLVERNLEDGLVEEIIFSSGGEIDVYDLQGLCDKVGWPRRPLVKLAAALKNS
GNAT2
AF2-M confidence
β1 α1 α2
β-sheets & α-helices
YMVATLHSVMKSSSDSDSSEGGDGEKQQEKKLIGMARATSDHAFNATIWDVLVDPEYQGQGLGKALVEKLVRALLQRDIGNISLFA
GNAT2
AF2-M confidence
β2 β3 β4 α3 β5
β-sheets & α-helices
DSQVVDFYQNLGFEADPEGIKGMFWYPK
GNAT2
AF2-M confidence
α4
β-sheets & α-helices
B
GNAT2
SGFVKNNNSTQLVEPPSIVNDEEEETEPLLPVEFTLVERNLEDGLVEEIIFSSGGEIDVYDLQGLCDKVGWPRRPLVKLAAALKNS
GNAT2
AF2-M confidence
β1 α1 α2
β-sheets & α-helices
YMVATLHSVMKSSSDSDSSEGGDGEKQQEKKLIGMARATSDHAFNATIWDVLVDPEYQGQGLGKALVEKLVRALLQRDIGNISLFA
GNAT2
AF2-M confidence
β2 β3 β4 α3 β5
β-sheets & α-helices
DSQVVDFYQNLGFEADPEGIKGMFWYPK
GNAT2
AF2-M confidence
α4
β-sheets & α-helices
GNAT3
NAASALPRSIPIYISTLKKDINLEELRNLYSLCNHSCNRLSEKDSNVEKIVDMKKLRRAISRSDVIVSVFCKPQHVDVDDAVLYSE
GNAT3
AF2-M confidence
β1 α1 α2 β2
β-sheets & α-helices
EESLSSSLYTSEFGRQNKDDSFLGDLFQNAVPLTPSNGQLVGFGRAYSDYGLTASIHDLMVLPSLQRMGIGKLIVNRIVRLLTSRD
GNAT3
AF2-M confidence
β3 β4 α3
β-sheets & α-helices
IYDIAALCFEDERPFFKACGFGDDRMGSTTMMFTKSLEA
GNAT3
AF2-M confidence
β5 α4
β-sheets & α-helices
Very high (pLDDT > 90)
Confident (90 > pLDDT > 70)
Low (70 > pLDDT > 50)
Very low (pLDDT < 50)

Supplemental Figure 18. Structural organization of GNAT1, 2 and 3 predicted as part of the heterodimer constellations GNAT1-GNAT2 (A) and GNAT2-GNAT3 (B), respectively. Heterodimer formation was modeled by using AlphaFold 2 Multimer, whereby predicted transit peptide sequences were excluded from the procedure (43). The first rows show the amino acid sequence with orange color highlighting the residues that form the interaction surface, as indicated by the AlphaFold 2 Multimer modeling and visualized by PyMOL (Version 4.5 Schrödinger, LLC). In the second rows, the per-residue prediction accuracy is given for each position ranking from „Very high“ to „Very low“, as illustrated by the color code (42). The third rows highlight the residues, which are proposed to form α-helix (red) and β-sheet (blue) structural elements. Black boxes within the amino acid sequences mark the lysine residues involved in the crosslinking of GNAT1-GNAT2 and GNAT2-GNAT3, respectively.
